# Description of Microbial Communities of Phosphate Mine Wastes in Morocco, a Semi-Arid Climate, Using High-Throughput Sequencing and Functional Prediction

**DOI:** 10.3389/fmicb.2021.666936

**Published:** 2021-07-08

**Authors:** Najoua Mghazli, Laila Sbabou, Rachid Hakkou, Ahmed Ouhammou, Mariam El Adnani, Odile Bruneel

**Affiliations:** ^1^Center of Research Plants and Microbial Biotechnologies, Biodiversity and Environment, Team of Microbiology and Molecular Biology, Faculty of Sciences, Mohammed V University in Rabat, Rabat, Morocco; ^2^HydroSciences Montpellier, University of Montpellier, CNRS, IRD, Montpellier, France; ^3^IMED_Laboratory, Faculty of Science and Technology, Cadi Ayyad University (UCA), Marrakech, Morocco; ^4^Mining Environment and Circular Economy Program, Mohammed VI Polytechnic University (UM6P), Benguerir, Morocco; ^5^Laboratory of Microbial Biotechnologies, Agrosciences and Environment (BioMAgE), Team of Agrosciences, PhytoBiodiversity and Environment, Regional Herbarium ‘MARK’, Faculty of Sciences Semlalia, Cadi Ayyad University, Marrakech, Morocco; ^6^Resources Valorisation, Environment and Sustainable Development Laboratory, National School of Mines of Rabat, Mohammed V University in Rabat, Rabat, Morocco

**Keywords:** phosphate mine wastes, microbial communities, PICRUSt prediction, metabarcoding, biodiversity

## Abstract

Soil microbiota are vital for successful revegetation, as they play a critical role in nutrient cycles, soil functions, and plant growth and health. A rehabilitation scenario of the abandoned Kettara mine (Morocco) includes covering acidic tailings with alkaline phosphate mine wastes to limit water infiltration and hence acid mine drainage. Revegetation of phosphate wastes is the final step to this rehabilitation plan. However, revegetation is hard on this type of waste in semi-arid areas and only a few plants managed to grow naturally after 5 years on the store-and-release cover. As we know that belowground biodiversity is a key component for aboveground functioning, we sought to know if any structural problem in phosphate waste communities could explain the almost absence of plants. To test this hypothesis, bacterial and archaeal communities present in these wastes were assessed by 16S rRNA metabarcoding. Exploration of taxonomic composition revealed a quite diversified community assigned to 19 Bacterial and two Archaeal phyla, similar to other studies, that do not appear to raise any particular issues of structural problems. The dominant sequences belonged to *Proteobacteria*, *Bacteroidetes*, *Actinobacteria*, and *Gemmatimonadetes* and to the genera *Massilia*, *Sphingomonas*, and *Adhaeribacter*. LEfSe analysis identified 19 key genera, and metagenomic functional prediction revealed a broader phylogenetic range of taxa than expected, with all identified genera possessing at least one plant growth-promoting trait. Around 47% of the sequences were also related to genera possessing strains that facilitate plant development under biotic and environmental stress conditions, such as drought and heat.

## Introduction

Mining is vital for the global economy, but the extraction of valuable elements also produces large quantities of solid wastes corresponding to uneconomic materials such as waste rock, refuse material, tailings, sediment, roasted ore, and processing chemicals (Hudson-Edwards, 2016).

The mining sector, and particularly phosphate exploitation, is one of the pillars of the Moroccan economy. Among many closed mines in the country, Kettara mine located in a semi-arid region that was exploited for pyrrhotite, is particularly problematic (Hakkou et al., 2008a,b). Mining produced more than 5.2 million metric tons of pyrrhotite concentrate, producing acid mine drainage following rainfall events leading to groundwater contamination (Lghoul et al., 2014). Dominant winds also transport toxic dust and sulfur emissions, from the mine wastes to the village of Kettara (2,000 inhabitants, located in the immediate vicinity of the mine), polluting homes, cattle, and farmland, putting the population under significant environmental and health risks (Khalil et al., 2013; Babi et al., 2016). Morocco holds around three-quarters of the world’s phosphate reserves and is the leading exporter of phosphate and its derivatives (Edixhoven et al., 2013; Hakkou et al., 2016). According to a United States Geological Survey^1^ in 2019, Morocco produced around 36 million tons of phosphate rocks representing an economical currency inflow. However, open-pit mining and beneficiation processes including crushing, screening, washing, flotation, or chemical attack generate million tons of tailings called phosphate sludge every year, which is stockpiled over a huge area (Hakkou et al., 2016; Jalali et al., 2019; Elfadil et al., 2020; Trifi et al., 2020). In semi-arid areas, the poor quality of these phosphate mine wastes, containing small quantities of nutrients and contaminated by metals and fluoride, prevents good plant growth (Cross and Lambers, 2017; Jalali et al., 2019; Elfadil et al., 2020). In Morocco, huge piles of phosphate waste rock and tailing ponds that cover thousands of hectares, with very limited vegetation and subjected to wind and water erosion, not only spoil the landscape but are also a serious source of pollution (Hakkou et al., 2016). Revegetation of phosphate mine wastes has therefore become a national priority for Morocco.

The rehabilitation scheme for Kettara mine consists of depositing a layer of alkaline phosphate waste on top of the acidic tailings to limit infiltration of water and to control acid mine drainage generation (Ouakibi et al., 2013; Bossé et al., 2015). In semi-arid climates characterized by low annual precipitation and high evaporation rates, store-and-release covers can prevent percolation of water during wet periods and allow evaporation (or evapotranspiration) during dry periods thus preventing water from penetrating acidic wastes (Bossé et al., 2013). Lab tests (using columns) and field tests (with experimental cells constructed on-site) showed very good results, proving that water infiltration can be controlled, even during extreme rainfall events (Ouakibi et al., 2014; Bossé et al., 2015). The final step in this scheme is the revegetation of the phosphate cover to limit the dispersion of fine dust, but revegetation is highly challenging for naturally growing biota.

Microbial communities are essential for soil functioning and for the development and maintenance of plant diversity (Hassani et al., 2018; van Bruggen et al., 2019). Soil microorganisms perform essential roles in the development and maintenance of healthy soil structure, including the decomposition of biomass (cellulose degradation, etc.), the transformation or biomineralization of biogenic element, increasing the quantity and availability of nutrients, degrading or biotransforming pollutants, mobilizing/immobilizing metals, etc. (Schulz et al., 2013; Thavamani et al., 2017). Microorganisms can also influence the development of plants *via* nitrogen fixation, phosphorus solubilization, sequestration of iron by siderophores or phytohormones production, etc. (Goswami et al., 2016; Kong and Glick, 2017; Olanrewaju et al., 2017). They can also limit biotic (pathogenic) stresses and abiotic environmental stresses for plants, including drought, salinity, heat, or toxic metals, which are particularly common in alkaline mine tailings in semi-arid areas (Armada et al., 2014; Fahad et al., 2015; Kumar and Verma, 2018; Gupta and Pandey, 2019). Studies dedicated to plant–microorganism associations are currently a very active area of research, and the interest of using microbial communities to stimulate plant growth in damaged and polluted soils, in semi-arid areas has been demonstrated in many countries (Mendez and Maier, 2008; Nirola et al., 2016; Bruneel et al., 2019). Different methods are currently used to inoculate plants with microorganisms. Ju et al. (2020) demonstrated that plant root inoculation with plant growth-promoting (PGP) rhizobia increased the phytostabilization by improving alfalfa growth by decreasing Cu accumulation in shoots in a Cu-contaminated site. Seeds can also be soaked in a bacterial inoculum before germination, as shown by Ke et al. (2021) where PGP bacteria enhanced ryegrass growth and phytostabilization in Cu–Cd-polluted soil. Indeed, pot experiment revealed that PGP bacteria addition increased shoot and root biomasses and limited stress of ryegrass by decreasing metal uptake and translocation. Direct application of bacterial inocula in contaminated soils has also shown positive results for the phytoextraction of excess phosphorus in soils (Ye et al., 2020; Guo et al., 2021). Guo et al. (2021) showed, for example, in pot experiments that two native phosphate-solubilizing microorganisms increased phosphate availability by reducing soil pH. Inoculation of these strains in soil also significantly increased the total biomass of two native plants, like their root and shoot length, and chlorophyll content.

As only a few plants managed to grow naturally after 5 years on the store-and-release cover, we sought to know if any structural problem in phosphate waste communities could explain the almost absence of plants. The aims of the present work were thus to (i) investigate the structure of the microbial communities (using 16S rRNA metabarcoding approach) and (ii) identify their potential metabolic functions in phosphate mine wastes using PICRUSt2 prediction, to test this hypothesis. To our knowledge, this is the first study to describe the microbial communities present in alkaline phosphate mine wastes in Morocco using high-throughput sequencing and the first study on overburden phosphate waste rocks and phosphate sludge issuing from processing. This phylogenetic classification of microbial communities of phosphate mine wastes and *in silico* identification of their potential plant growth-promoting diversity will provide useful information to prepare media dedicated to the isolation of the most interesting plant growth-promoting microorganisms identified in this study, helping revegetation in a semi-arid climate.

## Materials and Methods

### Description of Study Site and Experimental Cells

To evaluate the feasibility and effectiveness of using alkaline phosphate mine wastes as a store-and-release layer to limit infiltration of water in acidogenic tailing mine, field investigations were conducted at Kettara mine (31°52′15″N–8°10′31″W). Instrumented experimental cells (10 m × 10 m), with different configurations of store-and-release layer, and comprising two types of mine wastes, phosphate waste rock (overburden waste, removed to reach the phosphate ore) and phosphate sludge (produced during the concentration processes) were constructed in 2011 (Bossé et al., 2015; Table 1 and Supplementary Figure 1). The final step in the scheme of rehabilitation is the revegetation of the phosphate cover to limit the dispersion of fine dust, but after 5 years, only a few plants naturally grew on the experimental cells. Like belowground biodiversity is a key component for aboveground functioning, we conducted an *in situ* investigation, to identify bacterial and archaeal communities present in these experimental cells. To see if the presence of some annual plants, naturally growing, modified the structure of microbial communities, the microbial communities in soils sampled close to plants and in bare soils were also compared for each cell. Due to lack of plants naturally growing on these cells, three different species were chosen, *Spergularia rubra* (L.) for cell 1, *Atriplex semibaccata* for cell 3, and *Echium vulgare* (L.) for cell 4. *Echium vulgare* (L.) is a metallicolous plant that grows naturally in mine wastes and highly tolerant to Cd soil contamination (Dresler et al., 2015; Kompala-Baba et al., 2019). *Atriplex semibaccata*, found in abandoned mine, hyperaccumulate Cu and Cd and accumulate Fe (Baycu et al., 2015). *Atriplex* is also highly salt tolerant and has already been used as a pioneer species for revegetation in semi-arid areas (Nirola et al., 2016). *Spergularia rubra* (L) J.Presl and C.Presl has been found as a pioneer plant in mine tailings (Garcia-Carmona et al., 2019) and is known to accumulate Cu in shoots (Bech et al., 2017).

**TABLE 1:** Description of experiment cells (10 m × 10 m), constructed in 2011 to test their capacities to limit infiltration of water and comprising two types of phosphate wastes used as store and release cover (Bossé et al., 2015).

Type of material	Sample name	Thickness of cover	Fraction	Name of plants sampled	Sample code	Main composition*	Electrical conductivity**
Phosphate waste rock, overburden wastes, removed to reach the phosphate ore	Cell 1	100 cm	Raw material	*Spergularia rubra* (L.)	C1VS1 C1VS2 C1VS3	Calcite (40.7 wt%) Apatite (25.9 wt%) Dolomite (25.2 wt%) Quartz (8.2 wt%) Chromium (Cr, 137 mg/kg) Strontium (Sr, 769 mg/kg) Fluor (F, 1.3 wt%)	147 to 496 μS cm^–1^
				Bare soil	C1BS1 C1BS2 C1BS3		
	Cell 4	100 cm	< 1 mm	*Echium vulgare* (L.)	C4VS1 C4VS2 C4VS3		
				Bare soil	C4BS1 C4BS2 C4BS3		

Phosphate sludge, produced during the concentration processes	Cell 3	100 cm	Raw material	*Atriplex semibaccata*	C3VS1 C3VS2 C3VS3	Fluorapatite (44 wt%) Quartz (17 wt%) Calcite (15 wt%) Dolomite (7 wt%) Cadmium (Cd, 131 mg/kg) Chromium (Cr, 275 mg/kg) Strontium (Sr, 927 mg/kg) Fluor (F, 2.6 wt%).	178.6 to 691 μS cm^–1^
				Bare soil	C3BS1 C3BS2 C3BS3		

*According to Hakkou et al., 2009, 2016.

**Personnal communication from Rachid Hakkou.

### Sample Collection

In this study, three experimental cells already present were investigated (cells 1, 3, and 4, Bossé et al., 2015). For each cell, soil samples were collected in triplicate at the surface (0 to 5 cm) close to the roots of each plant (some centimeters from the roots) or in bulk soils located a few meters away from the plants to avoid the influence of plants. To obtain more homogenous representative samples, each of the three topsoil replicates was formed by mixing five soils, collected in 1 m^2^ subsamples of bare soil and in close proximity of the roots of five annual plants. These five samples were thoroughly homogenized to form three composite samples. Samples were named as follows: C1BS and C1VS for cell 1 with “BS” for bare soils and “VS” for soils with plants and with 1, 2, and 3 as replicates; C3BS and C3VS for cell 3, and C4BS and C4VS for cell 4. All 18 samples were immediately placed in Falcon tubes and stored in the field in coolers containing blocks of ice and transported to laboratory where they were stored at −20°C until DNA extraction. One tube from each site was stored at 4°C for physicochemical analyses.

### Physicochemical Characterization

The chemical composition of the samples was determined using X-ray fluorescence (XRF, Epsilon 4 Model, Malvern Panalytical). The device contains a kV tube with a silver (Ag) anode that produces X-rays. Its detector energy resolution was 4,000–50,000 kV. The samples were ground to a powder to obtain a particle size close to that of standards. The quality of the data was assessed using duplicate sample analyses, and measurement accuracy was estimated at ± 5% for all the elements analyzed. The total organic carbon was determined by potassium bichromate digester (Behrotest Heiz block K-40). The total nitrogen was measured using sulfuric acid–salicylic acid digestion following Kjeldahl method adjusted (Bremner, 1960) and determined by Kjeldahl K-375 BUCHI Kjel Master analyzer. The pH was measured by WTW/INOLAB pH7310 following the pH-meter instructions. Hundred milliliters of soil suspension was prepared in distilled water with a ratio of 1:5, then pH was measured using 1 mol/L of KCl.

### DNA Extraction and Amplification Using Illumina MiSeq Sequencing

Soil DNA was extracted in triplicates using the DNeasy^®^ PowerMax^®^ Soil kit Qiagen following the manufacturer’s instructions. All extracted genomic DNA samples were stored at –20°C until further analysis. Amplicon libraries were constructed according to ADNid^2^ dual indexing strategy. The universal primer set 519F (S-D-Arch-0519-a-S-15) (5′-CAGCMGCCGCGGTAA-3′) and 805R (S-D-Bact-0785-a-A-21) (5′-GACTACHVGGGTATCTAATCC-3′) designed to target bacterial and archaeal taxa (Klindworth et al., 2013) was used to amplify a 286-bp region targeting the V4 region of 16S rRNA genes. Amplicons were generated using Hot start DNA polymerase (QIAGEN). First, PCR (PCR1) was performed on 10 ng of template DNA in 15 μl total mix, submitted to an initial denaturation of 5 min at 95°C, followed by 35 cycles of 30 s at 95°C, 1 min at 60°C, and 30 s at 72°C. A final extension of 30 min at 60°C completed the reaction. Amplicons were checked on 1.5% agarose gel and purified using AMPure beads. A second PCR step (PCR2) was performed using a specific Illumina adapter, the index and the Illumina overhang adapter primers in 2 × KAPA Hifi Hotstart Ready Mix (Roche, Switzerland). A previously prepared 5-μl volume of PCR products was used to obtain a final volume of PCR mix of 50 μl. A 3-min denaturation step at 95°C was performed followed by 12 cycles of 30 s at 95°C, 30 s at 55°C, 30 s at 72°C, and a final 5-min extension step at 72°C. PCR2 products were purified (AMPure Beads), quantified (Nanodrop, Spark), normalized at 15 ng/μl, and pooled in a final library. The library was then purified using AMPure Beads, and its quality was checked using a fragment analyzer. Final quantification was performed by qPCR (KAPA library quantification Kits, Roche, Switzerland). Sequencing runs, generating 2 bp × 250 bp, reads were performed on an Illumina MiSeq using V2 chemistry.

### Sequence Analysis of Metabarcoding Datasets

The sequencing data analysis was conducted using FROGS v3.1.0 (Find Rapidly OTUs with Galaxy Solution, Escudié et al., 2018). Briefly, paired reads were merged using FLASH (v1.2.11, Magoc and Salzberg, 2011) with a minimum length of 240 bp and an overlap length of 270 bp. After denoising and removal of primer/adapters with cutadapt (v1.18, Martin, 2011), *de novo* clustering was implemented with SWARM (v2.2.2), which uses a local clustering threshold (Mahé et al., 2015), with an aggregation distance of *d* = 3. Chimeric sequences were removed using VSEARCH (v2.91, Rognes et al., 2016). The dataset was filtered, and sequences present in less than two samples and sequences representing less than 0.005% of the total set of sequences were discarded, as recommended by Bokulich et al. (2013). The taxonomic affiliation of operational taxonomic units (OTUs) was performed using Blastn^+^ against the Silva database (release 132, December 2017) for 16S rRNA gene amplicons using an innovative multi-affiliation output to identify conflicting sequences in the database and uncertainties (Escudié et al., 2018).

### Statistical Analysis and Predictive Metagenome Analysis

All statistical analyses were performed using R software^3^ unless otherwise stated. The alpha diversity statistics were calculated for each sample, and the rarefaction curves and the diversity indexes (Chao1, Shannon, and Simpson) were generated. The variation of beta diversity was graphically illustrated using non-metric multidimensional scaling (nMDS) analysis. Environmental variables were vector fitted to the nMDS ordination plots using the function “envfit” in the R “vegan” package to assess possible explanatory variables. Analysis of variance (ANOVA) was used at 95% confidence level and was completed by Tukey’s *post hoc* test to analyze significant distribution of the chemical parameters. Pearson correlations were used to assess the relationships between environmental factors, and the microbial community diversity. A linear discriminant analysis effect size (LEfSe) method (Segata et al., 2011) was also used to identify statistically significant microbial indicators associated with each cell and to identify the most discriminating clades. This analysis was done through the online interface at https://huttenhower.sph.harvard.edu/galaxy. Network analysis was used to reveal the relationship between genera present in bare soils and those present in soils sampled near plants. The preparation of data for network analysis was carried out using “psych” package as described by Batushansky et al. (2016). The “*r* coefficient” and “*p*-value” tables were generated, and only significant interactions were selected and visualized using Cytoscape V3.8.0 with the organic layout. The nodes represent the genera that differed significantly between bare soil and soil near the plant roots (*p* < 0.05) based on the results of ANOVA, and the edges represent the correlation between these genera.

Finally, PICRUSt2 (phylogenetic investigation of communities by reconstruction of unobserved states, Douglas et al., 2020) analysis was used to explore the possible functional profiles of the microbial communities. This was done following the pipeline command «picrust2_pipeline.py» using the “-stratified” flag as described in the PICRUSt2 GitHub^4^. The weighted Nearest Sequences Taxon Index (NSTI) was calculated to assess the accuracy of the PICRUSt2 analysis (Langille et al., 2013). The EC table resulting from PICRUSt2 was filtered, and only enzymes that contribute to pathways of interest in the present study were retained, i.e., phosphate solubilization, nitrogen metabolism, thiosulfate oxidation, cellulase degradation, and indole 3-acetic acid (IAA) and siderophore production. Phosphate solubilization in soil can be catalyzed by bacteria that produce enzymes including phosphatases, phosphonatases, phytases, and C-P lyase (Billah et al., 2019; Prabhu et al., 2019). For all other metabolisms, our study was based on the pathways published by the MetaCyc database^5^. To determine the presence of a function in one of the genera detected, all the enzymes involved in the given function’s pathway must be present in the same genus. The number of enzymes/functions was converted to “1” if present and to “0” if absent, and a presence/absence heatmap was then constructed using the “ggplot2” package.

## Results

### Physicochemical Characteristics of Phosphate Mine Wastes in Morocco

**FIGURE 1:**
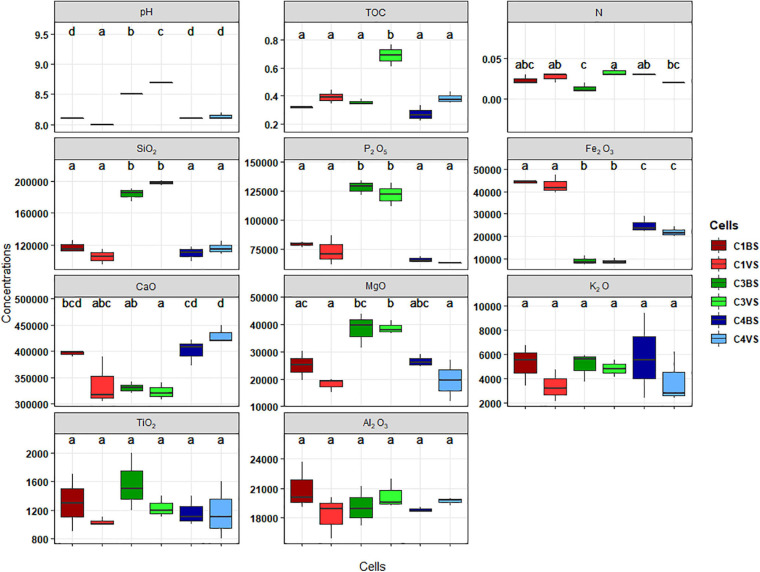
Boxplot displaying the concentrations of different chemical parameters. Organic carbon (OC) and nitrogen (N) are presented by percent. Major oxides such as silica (SiO_2_), phosphorus pentoxide (P_2_O_5_), ferric oxide (Fe_2_O_3_), lime (CaO), magnesia (MgO), potash (K_2_O), titanium oxide (TiO_2_), and alumina (Al_2_O_3_) are in milligrams per kilogram. The letters present the compact display letters (CDL). Error bars represent standard deviation of the mean value (analyses performed in triplicates). Values in each column followed by the same letter are not significantly different at 0.05% (Tukey’s).

The environmental characteristics of soil samples in the three cells are presented in Figure 1. ANOVA was used to test differences in geochemical factors among the samples and was completed with Tukey’s *post hoc* test at a confidence interval of 95%. Phosphate mining wastes were highly alkaline (pH 8–9) with low nutrient contents [total organic carbon (TOC) between 0.3 and 0.4%, and total nitrogen (N) < 0.034%] but very high level of P_2_O_5_ (> 75 g kg^–1^). Significant differences were observed between five parameters, pH, SiO_2_, P_2_O_5_, Fe_2_O_3_, and to a lesser extent in MgO between the two types of phosphate mine wastes, phosphate waste rocks (cells 1 and 4), and phosphate sludge (cell 3). pH, SiO_2_, P_2_O_5_, and MgO concentrations were statistically higher in phosphate sludge than in waste rocks. Inversely, concentrations of Fe_2_O_3_ were higher in waste rocks. No clear differences were found in many elements, including TOC, N, or CaO, between the three cells thus between the two types of phosphate wastes. No significant differences were found inside cells, between soils sampled near plants, and bare soils, except for TOC and N in cell 3, where the highest percentage was found in C3VS. No differences in K_2_O, TiO_2_, and Al_2_O_3_ were observed between samples.

### Diversity and Taxonomic Assignment of Microbial Communities in Phosphate Wastes

A total of 8,507,878 sequences paired-end reads were obtained from the samples collected in 18 phosphate mine wastes. After cleaning and processing, 7,633,309 high-quality sequences were obtained with an average of 428,414 sequences per sample. The number of sequences per sample was then made equal by random resampling (191,792 sequences per sample, based on the smaller number of sequences) giving 3,452,256 good-quality reads. At 97% similarity, all effective normalized sequences were distributed among 869 microbial OTUs (Supplementary Table 1). Rarefaction curves achieved an asymptote for all samples, suggesting that almost all OTUs were detected (Supplementary Figure 2). The number of OTUs estimated by Chao1 was also very similar to the richness (observed number of OTUs), ranging, respectively, from 650 to 800 for Chao1 and from 640 to 800 for richness, confirming that virtually all the microbial diversity was explored (Supplementary Figure 3). No statistical differences were observed between different cells but statistical differences were found between bare soils (C1BS, C3BS, and C4BS) and soils located near plants (C1VS, C3VS, and C4VS), with systematically higher richness and Chao1 in soils near plants than in bare soils. No statistical differences in Shannon and Simpson indices were found in the three cells or between bare soils and soils near plants, except for C4BS, which had lower Shannon and Simpson values.

**FIGURE 2:**
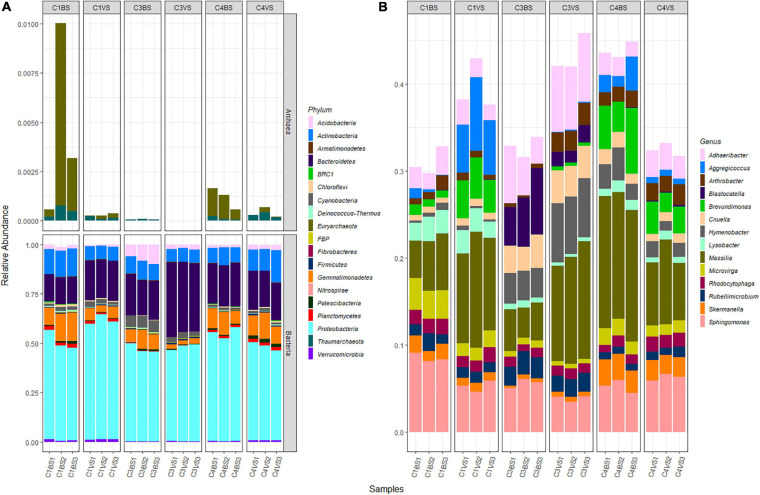
Microbial diversity present in the alkaline phosphate mining wastes. At the phylum level **(A)**, all sequences are presented, while at the genus level **(B)**, only the most abundant sequences are presented.

Exploration of taxonomic composition revealed that the 869 bacterial and archaeal OTUs were assigned to 19 phyla with only two Archaea (this kingdom represented only 0.11% of the total sequences, Figure 2A). Over 98% of the OTUs were assigned to a taxonomic phylum with 80% confidence. Across all 18 samples, *Proteobacteria* (51.3%), *Bacteroidetes* (22%), *Actinobacteria* (9.3%), and *Gemmatimonadetes* (7.9%) were the dominant phyla represented by more than 90% of all sequences. For Bacteria, at the genus level, 59.7% of sequences were attributed to known genera, 22.1% to unknown genera, and 18.2% were multi-affiliated (Supplementary Table 2). The majority of sequences were affiliated to the genera *Massilia* (9.4%), *Sphingomonas* (5.7%), *Adhaeribacter* (3.9%), and *Hymenobacter* (2.7%, Figure 2B and Supplementary Table 2). Only 18 genera had more than 1% of total sequences. For Archaea, up to 82.7% of sequences were affiliated to *Euryarchaeota* phylum and to unknown genera, while the remaining 17.3% were affiliated to *Thaumarchaeota* phylum, among which 70.8% were multi-affiliated to different genera of the *Nitrososphaeraceae* family and 29.2% were affiliated to the genus *Nitrososphaera*.

### Structure of Microbial Communities in Phosphate Mining Wastes and the Effect of Plants

**FIGURE 3:**
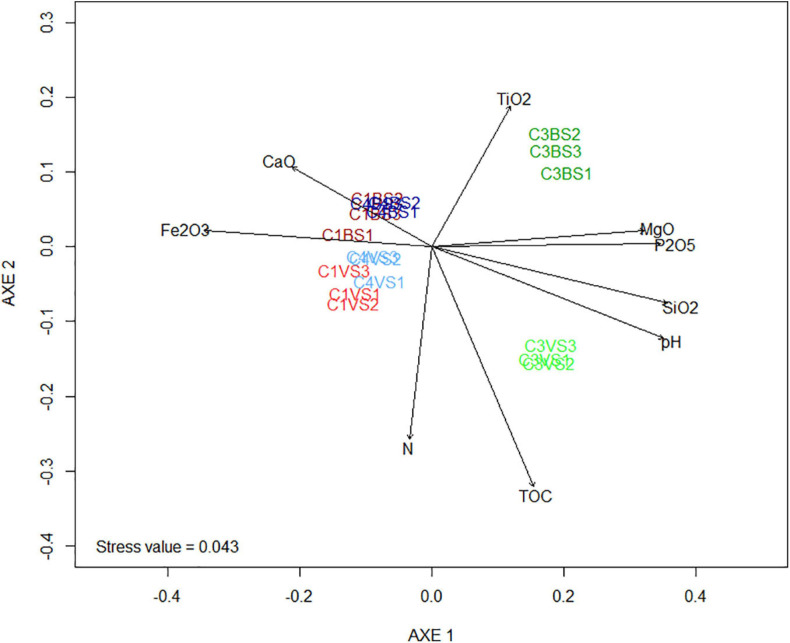
Non-metric multidimensional scaling (NMDS) presenting the differences of the microbial communities between the different replicates of phosphate mining waste samples. The stress values were < 0.05, indicating that these data were very well represented by the two-dimensional representation. Arrows are the projections of possible explanation variables obtained by vector fitting. Only correlations with a false discovery rate (fdr) corrected *p* < 0.05 were indicated. The angle and length of the vector indicate the direction and strength of the variable. The *r*^2^ correlation coefficient and the *p*-values are presented in Supplementary Table 3.

nMDS ordination and PERMANOVA was performed to explore the relationships between the structure of the microbial communities and the physicochemical characteristics of the phosphate mining wastes (Figure 3 and Supplementary Table 3). Phosphate sludge (six replicates of cell 3) and waste rocks (12 replicates of cell 1 and cell 4) were clearly separated along the first ordination axis. The second axis separated samples into two distinct groups, bare soils in all three cells and those of soils sampled near plants. This result was confirmed by statistical analysis using Jaccard dissimilarities (PERMANOVA, *p*-value = 0.002), indicating that community composition differed significantly between the two types of mine wastes and between soils near plants and bare soils (Supplementary Table 3). Vector fitting of environmental parameters on nMDS revealed that P_2_O_5_, pH, SiO_2_, and MgO were positively correlated with cell 3 and separated the two types of phosphate mining wastes (Figure 3 and Supplementary Table 4). Fe_2_O_3_ and, to a lesser degree, CaO, were negatively correlated with the first four parameters and were more abundant in cell 1 and cell 4. Vector fitting also revealed that TOC and, at a lesser degree, N, were positively correlated with soils near plants.

**FIGURE 4:**
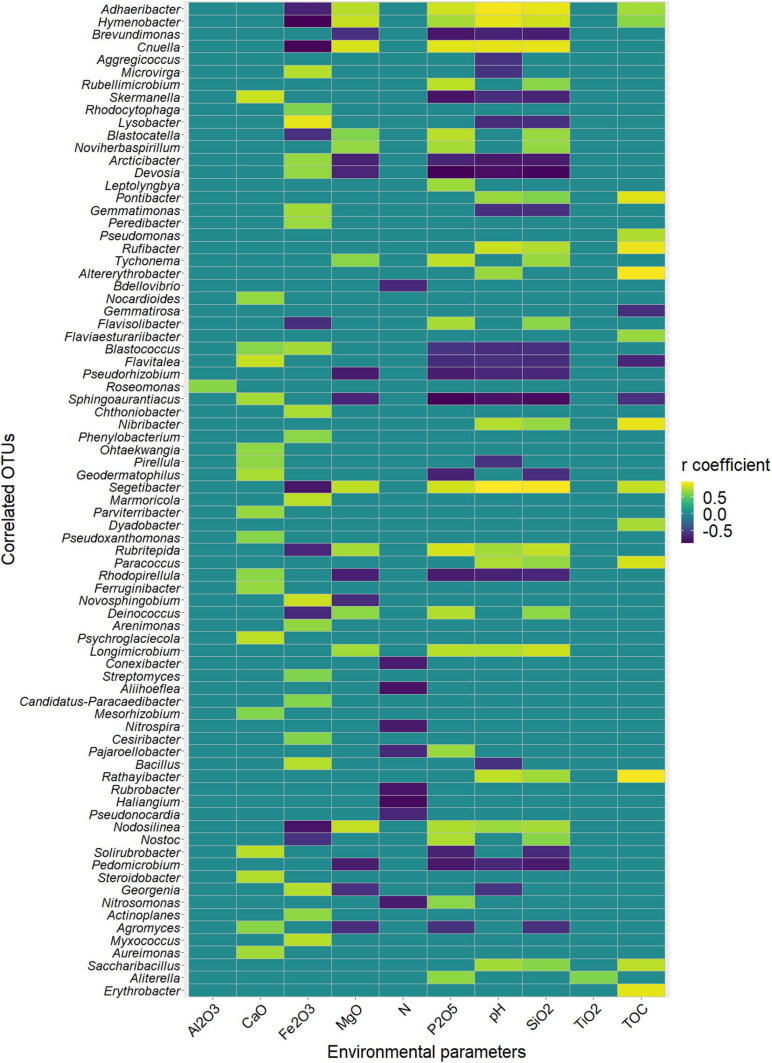
Heatmap of the statistically significant coefficients of Pearson’s correlations (false discovery rate (fdr) corrected; *p* < 0.05) between the relative abundance of taxa and concentrations of environmental parameters across all sites. Only threshold higher than 0.7 was plotted.

**FIGURE 5:**
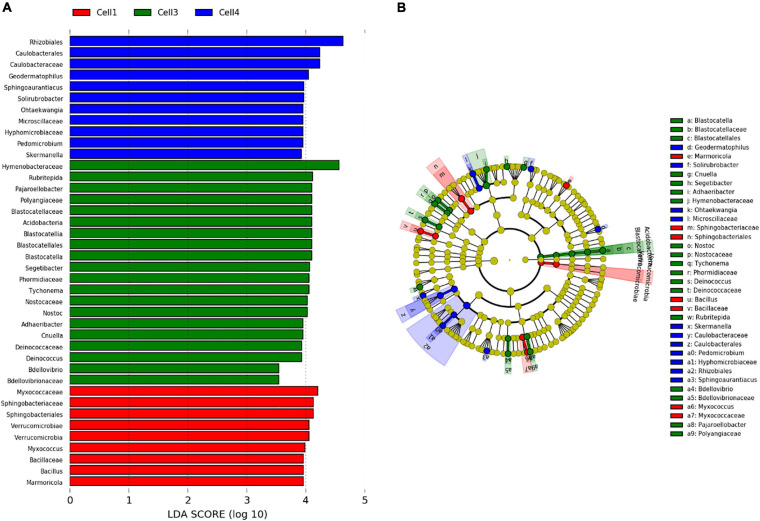
Linear discriminant analysis effect size (LEfSe), **(A)** chart and **(B)** cladogram, on microbial diversity in different store-and-release phosphate mine waste layer, overburden waste rock (cells 1 and 4), and phosphate mine wastes (cell 3) produced during the concentration processes.

**FIGURE 6:**
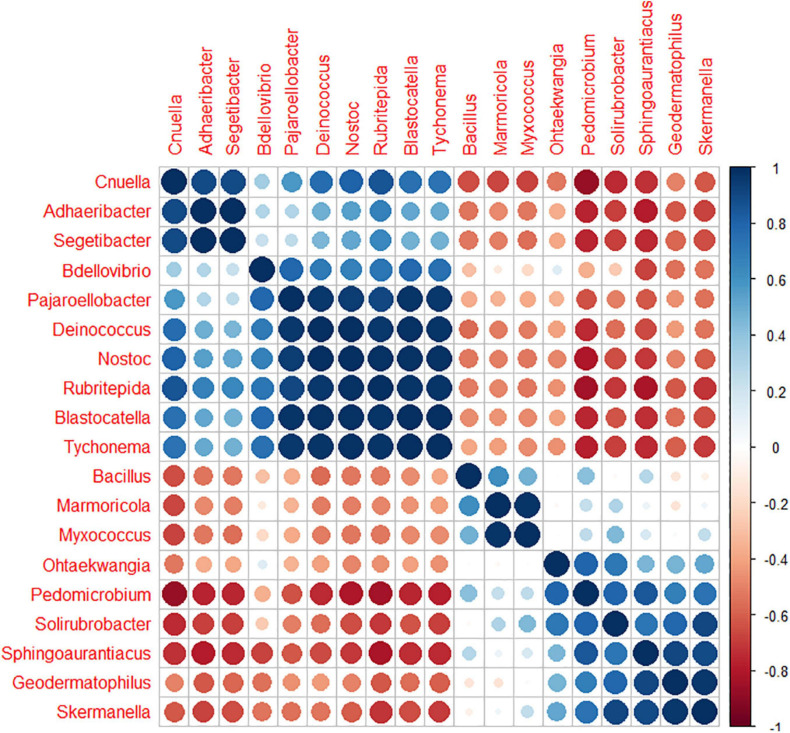
Correlogram representing the Pearson’s correlation coefficient *r*^2^ between the 19 key bacterial genera ordered by the “hclust” method.

Among the 130 identified genera, Pearson analysis indicated that 79 were significantly correlated with environmental variables (Figure 4). Many correlations were observed with SiO_2_ (35), P_2_O_5_ (31), pH (31), Fe_2_O_3_ (30), MgO (21), TOC (17), CaO (18), and N (9) but only one with Al_2_O_3_ and TiO_2_. For SiO_2_, P_2_O_5_, pH, Fe_2_O_3_, MgO, and TOC, 20, 18, 14, 20, 11, and 14 genera were, respectively, positively correlated, whereas 15, 13, 17, 10, 10, and 3 were negatively correlated. All 18 correlations found for CaO were positive, and all nine correlations found for N were negative. Very similar correlation indices were also observed between P_2_O_5_, pH, SiO_2_, and, to a lesser extent, MgO, with relatively dominant genera such as *Adhaeribacter*, *Hymenobacter*, and *Cnuella* positively affected, while *Brevundimonas*, *Arctibacter*, and *Devosia* were strongly negatively affected. The LEfSe analysis identified 9, 20, and 11 bacterial taxa, containing 3, 10, and 6 genera that statistically distinguished, respectively, cell 1, cell 3, and cell 4 (with LDA score > 2, Figure 5). The family *Hymenobacteraceae* was the most differentially abundant taxon with a LDA score > 4, reflecting higher abundance in cell 3 than in cells 1 and 4. At the genus level, *Adhaeribacter*, *Cnuella*, *Blastocatella*, *Tychonema*, *Bdellovibrio*, *Segetibacter*, *Rubritepedia*, *Deinococcus*, *Pajaroellobacter*, and *Nostoc* were overrepresented in cell 3. In cell 1, only *Marmoricola*, *Bacillus*, and *Myxococcus* were prevalent, while in cell 4, *Skermanella*, *Sphingoaurantiacus*, *Ohtaekwangia*, *Geodermatophilus*, *Solirubrobacter*, and *Pedomicrobium* were more abundant. These 19 genera were also highlighted by Pearson’s correlation. A correlogram of these core genera separated them into two distinct groups (Figure 6). The first group contained all genera found in the phosphate sludge (cell 3) identified by LEfSe and presented quite strong positive correlations among themselves and negative correlations among genera sampled in phosphate waste rocks (except for *Bdellovibrio*, which was positively correlated with *Ohtaekwangia*). The second group comprised genera retrieved in phosphate waste rocks (cells 1 and 4), and correlations between members of this group were generally positive, while correlations with phosphate sludge were negative.

Network analysis was also used to identify the relationships between genera found in bare soils and in soils sampled near annual plants (Supplementary Figure 4). Fifteen genera were found to be significantly associated with bare soils and 16 were shown to be more abundant in soils near plants.

### Potential Plant Growth Promotion Traits of Microbial Communities of Phosphate Wastes

**FIGURE 7:**
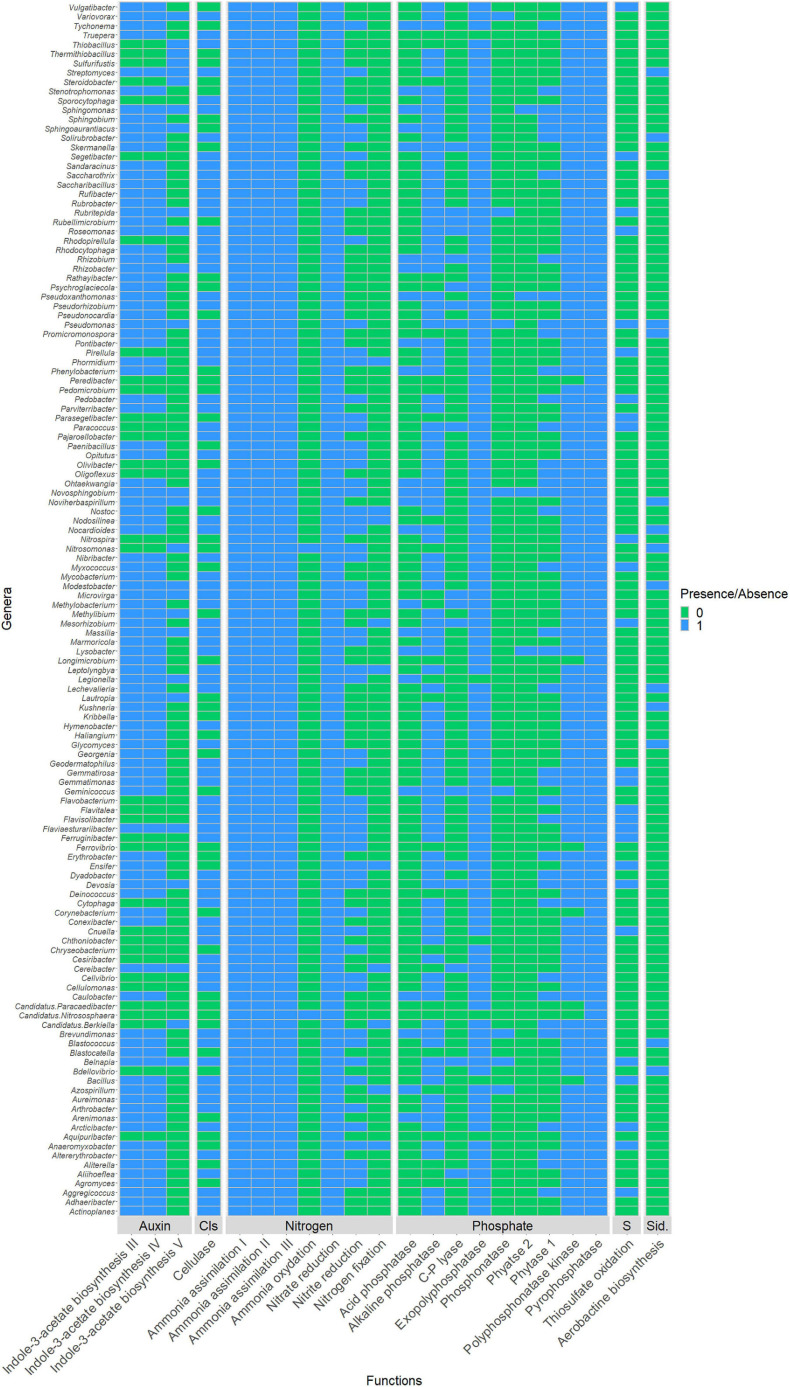
Binary heatmap highlighting presence (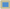) absence (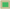) of the interested enzymes in identified genera. Enzymes implicated in: cellulose degradation by producing the cellulase enzyme (EC:3.2.1.4); sulfur metabolism by oxidizing thiosulfate (EC:1.8.2.2); siderophores production by catalyzing aerobactin biosynthesis pathway (EC:1.14.13.59, EC:2.3.1.102, EC:6.3.2.38 and EC:6.3.2.39); phosphate solubilization by producing either one or multiple of the enzymes C-P lyases (EC:4.7.1.1), phytase 1 (EC:3.1.3.8), phytase 2 (EC:3.1.3.26), alkaline phosphatase (EC:3.1.3.1), acid phosphatase (EC:3.1.3.2), and phosphonatase (EC:3.11.1.1); nitrogen metabolism by catalyzing at least one of the following pathways: nitrogen fixation I (ferredoxin) (EC:1.18.6.1), ammonia assimilation cycle I (EC:6.3.1.2 and EC:1.4.1.14); ammonia assimilation cycle II (EC:6.3.1.2 and EC:1.4.7.1), ammonia assimilation cycle III (EC:6.3.1.2 and EC:1.4.1.13), ammonia oxidation I and IV nitrite producing (EC:1.14.99.39 and EC:1.7.2.6), nitrate reduction VI L-glutamine forming (EC:1.7.7.2, EC:1.7.7.1, and EC:6.3.1.2), and nitrate reduction VI L-glutamate forming (EC:1.7.7.2, EC:1.7.7.1, and EC:1.4.1.4), and finally in auxin biosynthesis by producing enzymes implicated in indole-3-acetate biosynthesis III (EC:1.13.12.3 and EC:3.5.1.4, or in indole-3-acetate biosynthesis IV (EC:4.2.1.84 and EC:3.5.1.4) or in indole-3-acetate biosynthesis V (EC:3.5.5.1).

PICRUSt2 analysis was used to explore the possible metabolic pathways associated with microbial communities in phosphate wastes (Figure 7). The NSTI values < 0.06, confirmed the prediction accuracy (Supplementary Table 5). All 130 OTUs affiliated to known genera were predicted to possess at least one of the interesting enzymes involved in important traits related to plant development. For the phosphate solubilization, 102 genera were predicted to possess the alkaline phosphatase enzyme while only 39 were predicted to possess phytase 1, 23 acid phosphatase, 18 C-P lyase, 8 phosphonatase, and only 4 phytase 2. The OTUs assigned to *Novosphingobium* appeared to have all the phosphate-solubilizing enzymes except C-P lyase. For nitrogen metabolism, all genera were predicted to possess at least one of the seven enzymes involved in N assimilation and referenced in PICRUSt2 and also to produce nitrate-reducing enzymes. Only 2 genera were predicted to be able to oxidize ammonia (*Nitrosomonas* and the archaea *Nitrososphaera*), 59 were predicted to reduce nitrite, and 10 to fix nitrogen. For phytohormones production, 96 genera were predicted to possess at least one enzyme involved in different auxin biosynthesis pathways. Twenty-six genera were predicted to possess the thiosulfate oxidation enzyme and only 14 to biosynthesize aerobactin, an enzyme involved in siderophore production.

## Discussion

### Characteristics of Phosphate Mining Wastes and Development of Plants

Many mine tailings disposed in artificial dumps have not been subject to the long-term natural soil formation processes, representing challenging conditions for biota (Cross and Lambers, 2017). Mine wastes containing generally ultrafine particles, present also often dysfunctional and unstable physical and geochemical structures, altered or inadequate hydrological functioning, associated generally with extreme pH, limited concentrations of organic material and nutrients, and often presenting an accumulation of toxic or radioactive substances (Cross and Lambers, 2017; Cross et al., 2017). The phosphate mine wastes in the present study share many of these characteristics with highly alkaline soils (pH 8–9), very low levels of nutrients (TOC and N), very high concentrations of P_2_O_5_ (mass percent between 10% and 20%) and associated with high levels of hazardous contaminants (e.g., Sr, Cr, Cd, or F, Hakkou et al., 2016). Phosphate mine wastes mainly comprise ultrafine material with significant quantities of calcite and dolomite (Hakkou et al., 2009). In arid and semi-arid areas, soils present usually low moisture content and limited organic matter associated with extreme temperatures and irradiance (Padmavathiamma et al., 2014). They are also quite often saline, because of the high evaporation rate and the low water infiltration (Mendez and Maier, 2008). However, phosphate mine wastes in this study were not saline according to their electrical conductivity (Table 1). All these harsh properties could explain the almost absence of plants.

### Structure of Microbial Communities and Potential Physicochemical Drivers

Characterization of the microbial communities in phosphate wastes enabled most OTUs to be classified at the phylum level; whereas, only 55.3% of sequences were classified at the genus level. The high proportion of unclassified genera suggests the presence of possible novel microorganisms and is in agreement with the results of many studies conducted in mining environments (Bruneel et al., 2017; Liu et al., 2019). Around 90% of sequences belonged to only four phyla *Proteobacteria*, *Bacteroidetes*, *Actinobacteria*, and *Gemmatimonadetes*, and the dominant genera were identified as *Massilia*, *Sphingomonas*, *Adhaeribacter*, *Hymenobacter*, and *Brevundimonas*. Archaea formed a very small minority, and only one genus was identified, *Nitrososphaera*. Zouch et al. (2017) obtained similar results for phosphogypsum in Tunisia, where Archaea accounted for only 1.5% of the microbial community and no more than 3% in pyrrhotite mine tailings of Kettara (Bruneel et al., 2017). Among the 130 identified genera, only 18 had more than 1% of the total number of sequences. This is in agreement with the results obtained by Trifi et al. (2020), indicating that in Tunisia, phosphogypsum contained only five low abundance genera (between 1 and 5% of sequences) out of the 193 detected.

The composition of microbial communities differed significantly between the two types of mining wastes and was affected by soil chemical properties, particularly soil pH, P_2_O_5_, Fe_2_O_3_, MgO, CaO, and SiO_2_. Phosphorus and pH have been recognized as drivers of microbial communities, and pH is generally recognized to be the best predictor of microbial composition and diversity (Rousk et al., 2010; Wu et al., 2018; Ye et al., 2020).

Our investigation, combining Pearson correlations and LEfSe, also identified 19 genera divided into two groups, those found in phosphate sludge and those found in phosphate waste rocks. These core genera probably have significant functions in phosphate mining wastes, but, apart from *Bacillus*, *Nostoc*, and, to a lesser extent, *Myxococcus*, many of them are not known as PGP microorganisms such as *Adhaeribacter*, *Cnuella*, *Blastocatella*, *Tychonema*, *Sphingoaurantiacus*, *Segetibacter*, *Rubritepida*, *Deinococcus*, and *Pajaroellobacter* (Supplementary Table 6). Other genera possessed only few PGP traits: *Skermanella*, *Geodermatophilus*, and *Marmoricola* are involved in nitrogen cycling, *Ohtaekwangia* sp. displays antimicrobial activity, *Solirubrobacter* sp. is able to degrade cellulose, or *Bdellovibrio* and *Myxococcus*, which are known to be predators of other bacteria.

The composition of the microbial communities differed significantly between bare soils and soils sampled near plants. Richness and estimated OTUs were also higher in wastes associated with plants than in bare soils in all the three cells. Although our study did not analyze the rhizosphere, the proximity of plants appeared to have an impact on the structure of microbial communities, and organic carbon and N were identified as the main drivers. This is consistent with many studies reporting differences in the diversity and taxonomic composition of microbial communities in bulk soils and in the rhizosphere (Edwards et al., 2015; Luo et al., 2018; Orozco-Mosqueda et al., 2020; Ye et al., 2020). Plant species or even genotypes are also known to select divergent microbial communities (Bulgarelli et al., 2013; Edwards et al., 2015; Ye et al., 2020). This is usually explained by the presence of many elements exuded by roots including sugars, amino acids, and organic acids, which can account for one-third of the carbon fixed by plants (Bulgarelli et al., 2013; Edwards et al., 2015; Olanrewaju et al., 2017). Many significantly different genera were observed in soils near plants and in bare soils, but no clear differences were observed concerning their potential PGP traits as symbiotic N_2_ fixers, contrary with that reported by Luo et al. (2018). Although in agreement with Ye et al. (2020) in phosphogypsum, the relative abundance of three genera (*Blastocatella*, *Thermithiobacillus*, and *Sphingomonas*) was higher in soils associated with plants.

Other factors affect the abundance, structure, and activity of microbial communities, including the type, properties, and texture of the soil, as well as climatic conditions (Edwards et al., 2015; Luo et al., 2018; Ye et al., 2020). The structure of the microbial communities analyzed in this study differed from that identified in other studies conducted in phosphate mine wastes, such as in Ragot et al. (2013) using T-RFLP and clone libraries to study apatite, the principal phosphate mineral. Next-generation sequencing are generally used to investigate microbial communities in phosphate mining area in China (Ye et al., 2020) and in phosphogypsum in Portugal (Martins et al., 2016), in China (Wang et al., 2018), or in Tunisia (Zouch et al., 2017; ben Mefteh et al., 2019; Trifi et al., 2020). However, phosphogypsum, the main byproduct of phosphoric acid, obtained after sulfuric acid digestion, present clearly different physicochemical characteristics, compared with phosphate wastes in this study. Nevertheless, our microbial communities were quite similar to those associated with turfgrass seeds of three turf species (*Festuca rubra*, *Lolium arundinacea*, and *Lolium perenne*) in low moisture climates (annual precipitation < 750 mm), containing a majority of *Proteobacteria* (89%) followed by *Actinobacteria* (6%, Chen et al., 2020). At the genus level, Chen et al. (2020) found a high proportion of *Massilia* (25%), and *Pseudomonas* (29%), followed by *Sphingomonas* (6%), *Paenibacillus* (3%), *Pedobacter* (1%), *Flavobacterium*, and *Chryseobacterium* (< 1%). This structure is also quite similar to the core of phyllosphere communities composed of *Pseudomonas*, *Sphingomonas*, *Methylobacterium*, *Bacillus*, *Massilia*, *Arthrobacter*, and *Pantoea* (Bulgarelli et al., 2013).

### Potential Ecological Role in Plant Growth-Promoting Traits

Surprisingly, a huge number of microorganisms recognized as potential PGP genera, were found in alkaline phosphate mining wastes according to the PICRUSt2 predictions. Indeed, all 130 OTUs assigned to known genera were predicted to possess at least one enzyme involved in plant growth-promoting traits. These results are quite consistent with the literature and show that a very large proportion of our sequences (66.15%) resemble microorganisms that contain isolated strains known to present one plant growth-promoting trait (Supplementary Table 6). Many sequences were similar to very well-known PGP genera such as *Pseudomonas*, *Bacillus*, *Arthrobacter*, *Azospirillum*, *Herbaspirillum*, *Rhizobium*, *Flavobacterium*, *Variovorax*, *Streptomyces*, *Sphingomonas*, and *Paenibacillus* (Goswami et al., 2016, Supplementary Table 6). Some of these microorganisms are also currently sold in international markets as biofertilizers or biocontrol agents (Bio-Save^®^, Mycostop^®^, Galltrol-A, etc.) and are used by farmers in the field as inocula (Goswami et al., 2016; Pathak and Kumar, 2016; Kumari et al., 2019). Many of the most abundant sequences, representing more than 1% of the total sequences, also possessed many plant growth-promoting traits including *Massilia*, *Brevundimonas*, *Lysobacter*, or *Noviherbaspirillum* or genera belonging to *Hymenobacteraceae* family (identified by LEfSe as very abundant in phosphate sludge) comprising *Adhaeribacter*, *Hymenobacter*, *Pontibacter*, or *Rufibacter*.

According to PICRUSt2 prediction, all the microorganisms possessed at least one of the five enzymes involved in nitrogen cycling and more than 43 genera possessed isolated strains implicated in nitrogen cycling (Supplementary Table 6). Among them, *Rhizobium* is the main contributor to symbiotic nitrogen fixation in legume crops (Pathak and Kumar, 2016), but many free-living nitrogen fixers were also present, including *Azospirillum*, *Herbaspirillum*, *Bacillus*, or *Paenibacillus* (Goswami et al., 2016). *Azospirillum* is thought to fix from 5 to 20 kg/ha/year of N_2_, corresponding between 20 and 25% of total nitrogen required by maize or rice (Pathak and Kumar, 2016). For Archaea, *Nitrososphaera* is known to be an ammonia-oxidizing archaeon (Stieglmeier et al., 2014) and could thus contribute to the global nitrogen cycle in soils *via* nitrification (Hatzenpichler, 2012). According to metagenome functional prediction, 95% of the genera identified possess enzymes involved in phosphate solubilization and 44 of our sequences resembled genera containing isolated strains with phosphate solubilization capacity and comprising very well-known phosphate solubilizers (Supplementary Table 6). These results are in agreement with those of Khan et al. (2007) and Goswami et al. (2016), who showed that phosphate-solubilizing microorganisms could account for, respectively, from 1 to 50% and 20 to 40% of culturable populations in the soil. Among dominant genera, *Massilia* could also play a key role and a novel species, *M. phosphatilytica*, has been described with a phosphate-solubilizing potential of 40.32 μg in 48 h (Zheng et al., 2017). Potassium, calcium, or zinc solubilizers such as *Pseudomonas*, *Paenibacillus*, *Pontibacter*, *Variovorax*, or *Bacillus* improve plant nutrition and were also retrieved in our sequences (Supplementary Table 6). For iron, 14 genera could possess enzymes involved in this pathway, as predicted by PICRUSt2 and 36 sequences resembled genera containing isolated strains that produce siderophores (Supplementary Table 6). Sulfate is another important nutrient for plant development (Smith et al., 2000). PICRUSt2 prediction also identified genera increasing the availability of sulfate for plants, 26 genera potentially able to oxidize thiosulfate whereas two genera were found to contain isolated strains involved in sulfur metabolism such as *Thiobacillus* or *Stenotrophomonas*. These bacteria are also able to solubilize phosphate and K/Ca- or Na-bearing minerals due to the large quantity of sulfuric acid they produce (Ullah et al., 2013; Thabet et al., 2017). Microorganisms that produce cellulase which breaks cellulose down, also facilitate plant growth (El-Sayed et al., 2014). PICRUSt2 predicted that 83 genera possess enzymes involved in this pathway, but only eight genera possessed strains able to degrade cellulose. PGP microorganisms also improve soil formation, texture, and structure, by helping to aggregate soil particles through the production of exopolysaccharides that promote the formation of strong biofilms and hence the formation of soil crust (Kaushal and Wani, 2016; Thavamani et al., 2017). Microorganisms such as *Leptolyngbya*, *Pseudomonas*, or *Rhizobium* that are able to increase soil aggregation were retrieved in our phosphate mining wastes (Supplementary Table 6). For phytohormone, 96 genera should produce IAA according to PICRUSt2, while our literature survey indicated that 43 genera contain at least one isolated strain able to produce this hormone (Supplementary Table 6). PICRUSt2 prediction was in agreement with other studies showing that around 80% of microorganisms have been documented to produce IAA (Patten and Glick, 1996; Kochar et al., 2013). Strains producing other phytohormones were also retrieved including gibberellin (20 strains) and cytokinin (10 strains, Supplementary Table 6).

In alkaline phosphate mining wastes, more than 27 sequences resembled genera containing isolated strains that produce ACCD, able to inhibit the formation of ethylene, the stress hormone with *Sphingomonas*, *Brevundimonas*, *Arthrobacter*, *Devosia*, or *Pseudomonas* being the most abundant (Supplementary Table 6). Many microorganisms known to be capable of combat phytopathogens were also retrieved in the present study: *Pseudomonas*, *Bacillus*, *Streptomyces*, *Stenotrophomonas*, *Rhizobium*, or *Streptomyces*, as well as less well-known microorganisms, such as *Massilia* or *Sphingomonas* (Supplementary Table 6). *Pseudomonas* sp. is able to inhibit diseases caused by many pathogens and possesses antimicrobial, antifungal, and antiviral properties, in addition to providing protection against insects (Kumari et al., 2019; Pattnaik et al., 2019, Supplementary Table 6). These fast-growing genera induce systemic resistance and produce different compounds including antibiotics, polysaccharides, or siderophores (limiting iron for phytopathogens, Santoyo et al., 2012). *Bacillus* sp. is known to produce a wide range of antibacterial and antifungal antibiotics (Goswami et al., 2016). *Streptomyces* are also known to produce many antibiotics (Olanrewaju and Babalola, 2019). Different genera, able to decrease or even suppress development of nematodes, were also retrieved, including *Arthrobacter*, *Pseudomonas*, *Streptomyces*, *Bacillus*, *Rhizobium*, or *Variovorax* (Topalovic et al., 2020). *Sphingomonas* sp. can suppress disease symptoms and limit the growth of the foliar pathogen of tomato, *Pseudomonas syringae* (Bulgarelli et al., 2013). Strains such as *Paenibacillus*, *Streptomyces*, *Bacillus*, or *Pseudomonas* produce lytic enzymes.

Many microorganisms present in our phosphate mining wastes are also related to strains able to improve growth of plants under drought stress; *Pseudomonas*, *Azospirillum*, *Paenibacillus*, *Rhizobium*, *Bacillus*, or *Variovorax* are among these well-known microorganisms (Supplementary Table 6). Indeed, *Azospirillum* sp. is able to mediate drought tolerance in many plants, even in deserts, by modifying root development and increasing water contents, grain yield, and mineral quality (Mg, K, and Ca) by producing IAA, gibberellin, or abscisic acid (a stress phytohormone that controls stomatal closure and stress signal transduction, Vurukonda et al., 2016; Fukami et al., 2018). *Bacillus* sp. is also known to increase the growth of *Lavandula* under drought conditions by producing IAA, improving physiology, increasing metabolic activity, and enhancing plant nutrition (Armada et al., 2014). Saline stress is also currently considered to be one of the main constraints affecting both plants and microorganisms (Kaushal and Wani, 2016; Etesami and Beattie, 2018; Numan et al., 2018; Gupta and Pandey, 2019). Even if electrical conductivity was not high in our study, the high evaporation rate in the area could lead to high salinity in soil surface. Many microorganisms can improve the growth of plants in saline conditions, including strains of *Stenotrophomonas*, *Azospirillum*, *Bacillus*, and *Pseudomonas* (Supplementary Table 6). *Pseudomonas* sp. has been shown to improve the tolerance of wheat seedlings and sorghum to high temperatures by increasing levels of cellular metabolites (Ali et al., 2009; Vurukonda et al., 2016).

Diverse sequences related to resistant genera to different types of metals were identified, reflecting the stressors present in phosphate mining wastes in Morocco (Supplementary Table 6). This was particularly true for three metals that are present in large quantities, Cd, Cr, and Sr. Twenty-six genera contained strains resistant to Cd, but only six were resistant to Cr and four to Sr. High concentrations of Cd can reduce photosynthesis, nutrient, and water uptake, leading to growth inhibition, chlorosis, etc., and finally to death (Yadav et al., 2010). Chromium excess inhibits plant growth, causes nutrient imbalance, chlorosis, and root injury, and affects germination (Yadav et al., 2010), while excess Sr usually inhibits plant growth and weight (Burger and Lichtscheidl, 2019). *Sphingomonas* sp. was shown to actively increase the growth and stress tolerance of many plants in the presence of metals such as cadmium (Asaf et al., 2020, Supplementary Table 6). Long et al. (2017) also highlighted the capacity of *Bacillus* to immobilize Sr from aqueous solution by sorption. Fluorine, also present in quite large quantities in this study, can have also detrimental effects on the environment and human health (Fordyce, 2011; Wang et al., 2018) and can affect the growth of microorganisms (Li et al., 2014). In our phosphate wastes, various microorganisms were related to strains known to be tolerant to fluorine including *Bacillus*, *Actinobacter*, or *Pseudomonas* (Wang et al., 2018, Supplementary Table 6).

In light of these results, the microbial community present in the phosphate store-and-release cover seems not to be responsible for the almost absence of plants, explaining that their supplement in sufficient quantities may help in the phytostabilization of the phosphate cover. Further studies need to be done in order to address this conundrum.

## Conclusion

This study examined the composition and structuration of bacterial and archaeal communities in alkaline phosphate mining wastes in a Moroccan mine. The dominant sequences belonged to *Proteobacteria*, *Bacteroidetes*, *Actinobacteria*, and *Gemmatimonadetes* and to the genera *Massilia*, *Sphingomonas*, *Adhaeribacter*, *Hymenobacter*, and *Brevundimonas*. Significant differences in the composition of microbial communities were found between the two types of mining wastes studied (phosphate waste rocks and phosphate sludge), and differences were also found due to the presence of some indigenous annual plants. Soil pH, P_2_O_5_, Fe_2_O_3_, MgO, CaO, SiO_2_, TOC, and N were, respectively, identified as the main drivers influencing microbial community structure. This study improves our knowledge of the microbial communities present in alkaline phosphate mining wastes. This work also highlights the very high abundance of predicted plant growth-promoting microorganisms that can improve soil formation, nutrient uptake, and alleviating the biotic and abiotic stresses of this harsh environment in semi-arid areas. Furthermore, a great majority of our sequences resemble genera possessing more than one potential plant growth trait, which could be very useful in revegetation projects. In light of these results, the exploration of taxonomic composition of the phosphate store-and-release cover seems not to be able to explain the almost absence of plants, with a quite diversified communities, similar to other studies, without any particular structural issues and with a majority of microorganisms presenting plant growth-promoting factors. This is probably due to the harsh conditions of this damaged soil in semi-arid area that could prevent the plants from growing *in situ*. Future efforts should be oriented toward isolation of these interesting genera, using them as an inoculum to help the growth of local plants in such harsh environment. An in-depth understanding of the phosphate mine waste microbial communities and of their potential metabolic capabilities can provide useful information to prepare media dedicated to the isolation of the most interesting plant growth-promoting microorganisms identified in this study, a work currently in progress.

## Data Availability Statement

The datasets presented in this study can be found in online repositories. The names of the repository/repositories and accession number(s) can be found below: https://www.ebi.ac.uk/ena, PRJEB39890.

## Author Contributions

OB, LS, ME, and RH designed and contributed to the conception of the work. AO identified the plants and contributed to the sampling along with NM, OB, and RH. NM performed the experiments. NM and OB analyzed the data. RH, ME, AO, and LS contributed to the data interpretation. NM and OB wrote the first draft of the manuscript. All authors reviewed and approved the final version of this manuscript.

## Conflict of Interest

The authors declare that the research was conducted in the absence of any commercial or financial relationships that could be construed as a potential conflict of interest.

## Footnotes

1 http://minerals.usgs.gov/minerals/pubs/commodity/phosphate_rock/

2 www.qualtech-groupe.com, Montpellier, France

3 v3.6.0, http://www.r-project.org/

4 https://github.com/picrust/picrust2/wiki/Full-pipeline-script

5 https://biocyc.org/

## References

[B1] Ali S. Z. Sandhya V. Grover M. Kishore N. Rao L. V. Venkateswarlu B. (2009). *Pseudomonas* sp. strain AKM-P6 enhances tolerance of sorghum seedlings to elevated temperatures. *Biol. Fertil. Soils* 46 45–55. 10.1007/s00374-009-0404-9

[B2] Armada E. Portela G. Roldán A. Azcón R. (2014). Combined use of beneficial soil microorganism and agrowaste residue to cope with plant water limitation under semiarid conditions. *Geoderma* 232–234 640–648. 10.1016/j.geoderma.2014.06.025

[B3] Asaf S. Numan M. Khan A. L. Al-Harrasi A. (2020). *Sphingomonas*: from diversity and genomics to functional role in environmental remediation and plant growth. *Crit. Rev. Biotechnol.* 40 138–152. 10.1080/07388551.2019.1709793 31906737

[B4] Babi K. Asselin H. Benzaazoua M. (2016). Stakeholders’ perceptions of sustainable mining in Morocco: a case study of the abandoned Kettara mine. *Extr. Indust. Soc.* 3 185–192. 10.1016/j.exis.2015.11.007

[B5] Batushansky A. Toubiana D. Fait A. (2016). Correlation-based network generation, visualization, and analysis as a powerful tool in biological studies: a case study in cancer cell metabolism. *BioMed. Res. Int.* 2016:8313272. 10.1155/2016/8313272 27840831 PMC5090126

[B6] Baycu G. Tolunay D. Ozden H. Csatari I. Karadag S. Agba T. (2015). An abandoned copper mining site in cyprus and assessment of metal concentrations in plants and soil. *Int. J. Phytoremed.* 17 622–631. 10.1080/15226514.2014.922929 25976876

[B7] Bech J. Roca N. Tume P. (2017). “Hazardous element accumulation in soils and native plants in areas affected by mining activities in south america,” in *Assessment, Restoration and Reclamation of Mining Influenced Soils*, eds Bini C. Bech J. Pashkevich M. A. (Amsterdam: Elsevier Inc), 419–461. 10.1016/B978-0-12-809588-1.00016-5

[B8] ben Mefteh F. Bouket A. C. Daoud A. Luptakova L. Alenezi F. N. Gharsallah N. (2019). Metagenomic insights and genomic analysis of phosphogypsum and its associated plant endophytic microbiomes reveals valuable actors for waste bioremediation. *Microorganisms* 7:382. 10.3390/microorganisms7100382 31547633 PMC6843645

[B9] Billah M. Khan M. Bano A. Hassan T. U. Munir A. Gurmani A. R. (2019). Phosphorus and phosphate solubilizing bacteria: keys for sustainable agriculture. *Geomicrobiol. J.* 36 904–916. 10.1080/01490451.2019.1654043

[B10] Bokulich N. A. Subramanian S. Faith J. J. Gevers D. Gordon J. I. Knight R. (2013). Quality-filtering vastly improves diversity estimates from *Illumina amplicon* sequencing. *Nat. Methods* 10 57–59. 10.1038/nmeth.2276 23202435 PMC3531572

[B11] Bossé B. Bussière B. Hakkou R. Maqsoud A. Benzaazoua M. (2013). Bewertung von phosphat-kalkstein-abraum als bestandteil einer speicher-verdunstungs-abdeckung unter semiariden bedingungen. *Mine Water Environ.* 32 152–167. 10.1007/s10230-013-0225-9

[B12] Bossé B. Bussière B. Hakkou R. Maqsoud A. Benzaazoua M. (2015). Field experimental cells to assess hydrogeological behaviour of store-and-release covers made with phosphate mine waste. *Can. Geotech. J.* 52 1255–1269. 10.1139/cgj-2014-0263 33356898

[B13] Bremner J. M. (1960). Determination of nitrogen in soil by the Kjeldahl method. *J. Agric. Sci.* 55 11–33. 10.1017/s0021859600021572

[B14] Bruneel O. Mghazli N. Hakkou R. Dahmani I. Filali Maltouf A. Sbabou L. (2017). In-depth characterization of bacterial and archaeal communities present in the abandoned *Kettara pyrrhotite* mine tailings (Morocco). *Extremophiles* 21 671–685. 10.1007/s00792-017-0933-3 28447266

[B15] Bruneel O. Mghazli N. Sbabou L. Héry M. Casiot C. Filali-Maltouf A. (2019). “Role of microorganisms in rehabilitation of mining sites, focus on Sub Saharan African countries,” in *Journal of Geochemical Exploration*, Vol. 205 ed. Albanese S. (Amsterdam: Elsevier B.V), 10.1016/j.gexplo.2019.06.009

[B16] Bulgarelli D. Schlaeppi K. Spaepen S. van Themaat E. V. L. Schulze-Lefert P. (2013). Structure and functions of the bacterial microbiota of plants. *Annu. Rev. Plant Biol.* 64 807–838. 10.1146/annurev-arplant-050312-120106 23373698

[B17] Burger A. Lichtscheidl I. (2019). Strontium in the environment: review about reactions of plants towards stable and radioactive strontium isotopes. *Sci. Total Environ.* 653 1458–1512. 10.1016/j.scitotenv.2018.10.312 30759584

[B18] Chen Q. Meyer W. A. Zhang Q. White J. F. (2020). 16S rRNA metagenomic analysis of the bacterial community associated with turf grass seeds from low moisture and high moisture climates. *PeerJ* 2020:e8417. 10.7717/peerj.8417 31942261 PMC6956778

[B19] Cross A. T. Lambers H. (2017). Young calcareous soil chronosequences as a model for ecological restoration on alkaline mine tailings. *Sci. Total Environ.* 607–608 168–175. 10.1016/j.scitotenv.2017.07.005 28689121

[B20] Cross A. T. Stevens J. C. Dixon K. W. (2017). One giant leap for mankind: can ecopoiesis avert mine tailings disasters? *Plant Soil* 421 1–5. 10.1007/s11104-017-3410-y

[B21] Douglas G. M. Maffei V. J. Zaneveld J. R. Yurgel S. N. Brown J. R. Taylor C. M. (2020). PICRUSt2 for prediction of metagenome functions. *Nat. Biotechnol.* 38 685–688. 10.1038/s41587-020-0548-6 32483366 PMC7365738

[B22] Dresler S. Tyrka M. Szeliga M. Ciura J. Wielbo J. Wójcik M. (2015). Increased genetic diversity in the populations of *Echium vulgare* L. colonising Zn-Pb waste heaps. *Biochem. Syst. Ecol.* 60 28–36. 10.1016/j.bse.2015.03.003

[B23] Edixhoven J. D. Gupta J. Savenije H. H. G. (2013). Recent revisions of phosphate rock reserves and resources: reassuring or misleading? An in-depth literature review of global estimates of phosphate rock reserves and resources. *Earth Syst. Dyn. Discuss.* 4 1005–1034. 10.5194/esdd-4-1005-2013

[B24] Edwards J. Johnson C. Santos-Medellín C. Lurie E. Podishetty N. K. Bhatnagar S. (2015). Structure, variation, and assembly of the root-associated microbiomes of rice. *Proc. Natl. Acad. Sci. U.S.A.* 112 E911–E920. 10.1073/pnas.1414592112 25605935 PMC4345613

[B25] Elfadil S. Hamamouch N. Jaouad A. Mahrouz M. Bouchdoug M. (2020). The effect of phosphate flotation wastes and phosphogypsum on cattle manure compost quality and plant growth. *J. Mater. Cycles Waste Manag.* 22 996–1005. 10.1007/s10163-020-00997-5

[B26] El-Sayed W. S. Akhkha A. El-Naggar M. Y. Elbadry M. (2014). In vitro antagonistic activity, plant growth promoting traits and phylogenetic affiliation of rhizobacteria associated with wild plants grown in arid soil. *Front. Microbiol.* 5:651. 10.3389/fmicb.2014.00651 25538687 PMC4255609

[B27] Escudié F. Auer L. Bernard M. Mariadassou M. Cauquil L. Vidal K. (2018). FROGS: find, rapidly, OTUs with galaxy solution. *Bioinformatics* 34 1287–1294. 10.1093/bioinformatics/btx791 29228191

[B28] Etesami H. Beattie G. A. (2018). Mining halophytes for plant growth-promoting halotolerant bacteria to enhance the salinity tolerance of non-halophytic crops. *Front. Microbiol.* 9:148. 10.3389/fmicb.2018.00148 29472908 PMC5809494

[B29] Fahad S. Hussain S. Matloob A. Khan F. A. Khaliq A. Saud S. (2015). Phytohormones and plant responses to salinity stress: a review. *Plant Growth Regul.* 75 391–404. 10.1007/s10725-014-0013-y

[B30] Fordyce F. M. (2011). “Fluorine: human health risks,” in *Encyclopedia of Environmental Health*, Vol. 2 ed. Nriagu J. O. (Burlington: Elsevier), 776–785.

[B31] Fukami J. Cerezini P. Hungria M. (2018). *Azospirillum*: benefits that go far beyond biological nitrogen fixation. *AMB Express* 8:73. 10.1186/s13568-018-0608-1 29728787 PMC5935603

[B32] Garcia-Carmona M. Garcia-Robles H. Turpín Torrano C. Fernández Ondoño E. Lorite Moreno J. Sierra Aragón M. (2019). Residual pollution and vegetation distribution in amended soils 20 years after a pyrite mine tailings spill (Aznalcóllar, Spain). *Sci. Total Environ.* 650 933–940. 10.1016/j.scitotenv.2018.09.092 30308867

[B33] Goswami D. Thakker J. N. Dhandhukia P. C. (2016). Portraying mechanics of plant growth promoting rhizobacteria (PGPR): a review. *Cogent Food Agric.* 2:1127500. 10.1080/23311932.2015.1127500

[B34] Guo S. Feng B. Xiao C. Wang Q. Chi R. (2021). Phosphate-solubilizing microorganisms to enhance phytoremediation of excess phosphorus pollution in phosphate mining wasteland soil. *Bioremed. J.* 1–15. 10.1080/10889868.2021.1884528

[B35] Gupta S. Pandey S. (2019). ACC deaminase producing bacteria with multifarious plant growth promoting traits alleviates salinity stress in French Bean (*Phaseolus vulgaris*) plants. *Front. Microbiol.* 10:1506. 10.3389/fmicb.2019.01506 31338077 PMC6629829

[B36] Hakkou R. Benzaazoua M. Bussière B. (2008a). Acid mine drainage at the abandoned Kettara mine (Morocco): 1. environmental characterization. *Mine Water Environ.* 27 145–159. 10.1007/s10230-008-0036-6

[B37] Hakkou R. Benzaazoua M. Bussière B. (2008b). Acid mine drainage at the abandoned Kettara mine (Morocco): 2. mine waste geochemical behavior. *Mine Water Environ.* 27 160–170. 10.1007/s10230-008-0035-7

[B38] Hakkou R. Benzaazoua M. Bussière B. (2009). Laboratory evaluation of the use of alkaline phosphate wastes for the control of acidic mine drainage. *Mine Water Environ.* 28 206–218. 10.1007/s10230-009-0081-9

[B39] Hakkou R. Benzaazoua M. Bussière B. (2016). Valorization of phosphate waste rocks and sludge from the moroccan phosphate mines: challenges and perspectives. *Proc. Eng.* 138 110–118. 10.1016/j.proeng.2016.02.068

[B40] Hassani M. A. Durán P. Hacquard S. (2018). Microbial interactions within the plant holobiont. *Microbiome* 6:58. 10.1186/s40168-018-0445-0 29587885 PMC5870681

[B41] Hatzenpichler R. (2012). Diversity, physiology, and niche differentiation of ammonia-oxidizing archaea. *Appl. Environ. Microbiol.* 78 7501–7510. 10.1128/AEM.01960-12 22923400 PMC3485721

[B42] Hudson-Edwards K. (2016). Tackling mine wastes. *Science* 352 288–290. 10.1126/science.aaf3354 27081053

[B43] Jalali J. Gaudin P. Capiaux H. Ammar E. Lebeau T. (2019). Fate and transport of metal trace elements from phosphogypsum piles in Tunisia and their impact on soil bacteria and wild plants. *Ecotoxicol. Environ. Safety* 174 12–25. 10.1016/j.ecoenv.2019.02.051 30802673

[B44] Ju W. Jin X. Liu L. Shen G. Zhao W. Duan C. (2020). Rhizobacteria inoculation benefits nutrient availability for phytostabilization in copper contaminated soil: drivers from bacterial community structures in rhizosphere. *Appl. Soil Ecol.* 150:103450. 10.1016/j.apsoil.2019.103450

[B45] Kaushal M. Wani S. P. (2016). Rhizobacterial-plant interactions: strategies ensuring plant growth promotion under drought and salinity stress. *Agric. Ecosyst. Environ.* 231 68–78. 10.1016/j.agee.2016.06.031

[B46] Ke T. Guo G. Liu J. Zhang C. Tao Y. Wang P. (2021). Improvement of the Cu and Cd phytostabilization efficiency of perennial ryegrass through the inoculation of three metal-resistant PGPR strains. *Environ. Pollut.* 271:116314. 10.1016/j.envpol.2020.116314 33360656

[B47] Khalil A. Hanich L. Bannari A. Zouhri L. Pourret O. Hakkou R. (2013). Assessment of soil contamination around an abandoned mine in a semi-arid environment using geochemistry and geostatistics: pre-work of geochemical process modeling with numerical models. *J. Geochem. Explor.* 125 117–129. 10.1016/j.gexplo.2012.11.018

[B48] Khan M. S. Zaidi A. Wani P. A. (2007). Role of phosphate-solubilizing microorganisms in sustainable agriculture – a review. *Agron. Sustain. Dev.* 27 29–43. 10.1051/agro:2006011

[B49] Klindworth A. Pruesse E. Schweer T. Peplies J. Quast C. Horn M. (2013). Evaluation of general 16S ribosomal RNA gene PCR primers for classical and next-generation sequencing-based diversity studies. *Nucleic Acids Res.* 41:e1. 10.1093/nar/gks808 22933715 PMC3592464

[B50] Kochar M. Vaishnavi A. Upadhyay A. Srivastava S. (2013). “Bacterial biosynthesis of indole-3-acetic acid: signal messenger service,” in *Molecular Microbial Ecology of the Rhizosphere*, Vol. 1 ed. de Bruijn F. J. (Hoboken, NJ: John Wiley and Sons), 309–325. 10.1002/9781118297674.ch29

[B51] Kompala-Baba A. Bierza W. Błońska A. Sierka E. Magurno F. Chmura D. (2019). Vegetation diversity on coal mine spoil heaps – how important is the texture of the soil substrate? *Biologia* 74 419–436. 10.2478/s11756-019-00218-x

[B52] Kong Z. Glick B. R. (2017). The role of plant growth-promoting bacteria in metal phytoremediation. *Adv. Microb. Physiol.* 71 97–132. 10.1016/bs.ampbs.2017.04.001 28760324

[B53] Kumar A. Verma J. P. (2018). Does plant—Microbe interaction confer stress tolerance in plants: a review? *Microbiol. Res.* 207 41–52. 10.1016/j.micres.2017.11.004 29458867

[B54] Kumari B. Mallick M. A. Solanki M. K. Solanki A. C. Hora A. Guo W. (2019). “Plant growth promoting rhizobacteria (pgpr): modern prospects for sustainable agriculture,” in *Plant Health Under Biotic Stress*, eds Mahmood I. Ansari R. A. (Singapore: Springer), 109–127. 10.1007/978-981-13-6040-4_6

[B55] Langille M. G. I. Zaneveld J. Caporaso J. G. McDonald D. Knights D. Reyes J. A. (2013). Predictive functional profiling of microbial communities using 16S rRNA marker gene sequences. *Nat. Biotechnol.* 31 814–821. 10.1038/nbt.2676 23975157 PMC3819121

[B56] Lghoul M. Maqsoud A. Hakkou R. Kchikach A. (2014). Hydrogeochemical behavior around the abandoned Kettara mine site, Morocco. *J. Geochem. Explor.* 144 456–467. 10.1016/j.gexplo.2013.12.003

[B57] Li X. Huang L. Bond P. L. Lu Y. Vink S. (2014). Bacterial diversity in response to direct revegetation in the Pb-Zn-Cu tailings under subtropical and semi-arid conditions. *Ecol. Eng.* 68 233–240. 10.1016/j.ecoleng.2014.03.044

[B58] Liu J. L. Yao J. Wang F. Min N. Gu J. H. Li Z. F. (2019). Bacterial diversity in typical abandoned multi-contaminated nonferrous metal(loid) tailings during natural attenuation. *Environ. Pollut.* 247 98–107. 10.1016/j.envpol.2018.12.045 30669085

[B59] Long J. Li H. Jiang D. Luo D. Chen Y. Xia J. (2017). Biosorption of strontium (II) from aqueous solutions by *Bacillus cereus* isolated from strontium hyperaccumulator *Andropogon gayanus*. *Process Safety Environ. Prot.* 111 23–30. 10.1016/j.psep.2017.06.010

[B60] Luo Y. Wu Y. Wang H. Xing R. Zheng Z. Qiu J. (2018). Bacterial community structure and diversity responses to the direct revegetation of an artisanal zinc smelting slag after 5 years. *Environ. Sci. Pollut. Res.* 25 14773–14788. 10.1007/s11356-018-1573-6 29541981

[B61] Magoc T. Salzberg S. L. (2011). FLASH: fast length adjustment of short reads to improve genome assemblies. *Bioinformatics* 27 2957–2963. 10.1093/bioinformatics/btr507 21903629 PMC3198573

[B62] Mahé F. Rognes T. Quince C. de Vargas C. Dunthorn M. (2015). Swarmv2: highly-scalable and high-resolution amplicon clustering. *PeerJ.* 3:e1420. 10.7717/peerj.1420 26713226 PMC4690345

[B63] Martin M. (2011). Cutadapt removes adapter sequences from high-throughput sequencing reads. *EMBnet J.* 17:10. 10.14806/ej.17.1.200

[B64] Martins M. Assunção A. Neto A. Silva G. Sghaier H. Costa M. C. (2016). Performance and bacterial community shifts during phosphogypsum biotransformation. *Water Air Soil Pollut.* 227:437. 10.1007/s11270-016-3129-z

[B65] Mendez M. O. Maier R. M. (2008). Phytoremediation of mine tailings in temperate and arid environments. *Rev. Environ. Sci. Biotechnol.* 7 47–59. 10.1007/s11157-007-9125-4

[B66] Nirola R. Megharaj M. Beecham S. Aryal R. Thavamani P. Vankateswarlu K. (2016). Remediation of metalliferous mines, revegetation challenges and emerging prospects in semi-arid and arid conditions. *Environ. Sci. Pollut. Res.* 23 20131–20150. 10.1007/s11356-016-7372-z 27539471

[B67] Numan M. Bashir S. Khan Y. Mumtaz R. Shinwari Z. K. Khan A. L. (2018). Plant growth promoting bacteria as an alternative strategy for salt tolerance in plants: a review. *Microbiol. Res.* 209 21–32. 10.1016/j.micres.2018.02.003 29580619

[B68] Olanrewaju O. S. Babalola O. O. (2019). Streptomyces: implications and interactions in plant growth promotion. *Appl. Microbiol. Biotechnol.* 103 1179–1188. 10.1007/s00253-018-09577-y 30594952 PMC6394478

[B69] Olanrewaju O. S. Glick B. R. Babalola O. O. (2017). “Mechanisms of action of plant growth promoting bacteria,” in *World Journal of Microbiology and Biotechnology*, Vol. 33 ed. Large P. J. (Dordrecht: Springer), 10.1007/s11274-017-2364-9 PMC5686270 28986676

[B70] Orozco-Mosqueda M. del C. Glick B. R. Santoyo G. (2020). ACC deaminase in plant growth-promoting bacteria (PGPB): an efficient mechanism to counter salt stress in crops. *Microbiol. Res.* 235:126439. 10.1016/j.micres.2020.126439 32097862

[B71] Ouakibi O. Hakkou R. Benzaazoua M. (2014). Phosphate carbonated wastes used as drains for acidic mine drainage passive treatment. *Proc. Eng.* 83 407–414. 10.1016/j.proeng.2014.09.049

[B72] Ouakibi O. Loqman S. Hakkou R. Benzaazoua M. (2013). The potential use of phosphatic limestone wastes in the passive treatment of AMD: a laboratory study. *Mine Water Environ.* 32 266–277. 10.1007/s10230-013-0226-8

[B73] Padmavathiamma P. K. Ahmed M. Rahman H. A. (2014). Phytoremediation - A sustainable approach for contaminant remediation in arid and semi-arid regions -a review. *Emirates J. Food Agric.* 26 757–772. 10.9755/ejfa.v26i9.18202

[B74] Pathak D. V. Kumar M. (2016). “Microbial inoculants as biofertilizers and biopesticides,” in *Microbial Inoculants in Sustainable Agricultural Productivity: Vol. 1: Research Perspectives*, eds Singh D. P. Singh H. B. Prabha R. (Delhi: Springer), 197–209. 10.1007/978-81-322-2647-5_11

[B75] Patten C. L. Glick B. R. (1996). Bacterial biosynthesis of indole-3-acetic acid. *Can. J. Microbiol.* 42 207–220. 10.1139/m96-032 8868227

[B76] Pattnaik S. Mohapatra B. Kumar U. Pattnaik M. Samantaray D. (2019). “Microbe-mediated plant growth promotion: a mechanistic overview on cultivable plant growth-promoting members,” in *Biofertilizers for Sustainable Agriculture and Environment. Soil Biology*, Vol. 55 eds Giri B. Prasad R. Wu Q. S. Varma A. (Cham: Springer), 435–463.

[B77] Prabhu N. Borkar S. Garg S. (2019). “Phosphate solubilization by microorganisms: overview, mechanisms, applications and advances,” in *Advances in Biological Science Research: A Practical Approach*, eds Naik M. Meena S. N. (Amsterdam: Elsevier), 161–176. 10.1016/B978-0-12-817497-5.00011-2

[B78] Ragot S. Zeyer J. Zehnder L. Reusser E. Brandl H. Lazzaro A. (2013). Bacterial community structures of an alpine apatite deposit. *Geoderma* 202–203 30–37. 10.1016/j.geoderma.2013.03.006

[B79] Rognes T. Flouri T. Nichols B. Quince C. Mahé F. (2016). VSEARCH: a versatile open source tool for metagenomics. *PeerJ* 4:e2584. 10.7717/peerj.2584 27781170 PMC5075697

[B80] Rousk J. Bååth E. Brookes P. C. Lauber C. L. Lozupone C. Caporaso J. G. (2010). Soil bacterial and fungal communities across a pH gradient in an arable soil. *ISME J.* 4 1340–1351. 10.1038/ismej.2010.58 20445636

[B81] Santoyo G. del Orozco-Mosqueda M. C. Govindappa M. (2012). “Mechanisms of biocontrol and plant growth-promoting activity in soil bacterial species of *Bacillus* and *Pseudomonas*: a review,” in *Biocontrol Science and Technology*, Vol. 22 ed. M. S. Goettel (Abingdon: Taylor & Francis Ltd), 855–872. 10.1080/09583157.2012.694413.

[B82] Schulz S. Brankatschk R. Dümig A. Kögel-Knabner I. Schloter M. Zeyer J. (2013). The role of microorganisms at different stages of ecosystem development for soil formation. *Biogeosciences* 10 3983–3996. 10.3929/ethz-b-000070776

[B83] Segata N. Izard J. Waldron L. Gevers D. Miropolsky L. Garrett W. S. (2011). Metagenomic biomarker discovery and explanation. *Genome Biol.* 12:R60. 10.1186/gb-2011-12-6-r60 21702898 PMC3218848

[B84] Smith F. W. Rae A. L. Hawkesford M. J. (2000). Molecular mechanisms of phosphate and sulphate transport in plants. *Biochim. Biophys. Acta BBA Biomembr.* 1465 236–245. 10.1016/s0005-2736(00)00141-3 10748257

[B85] Stieglmeier M. Klingl A. Alves R. J. E. Rittmann S. K. M. R. Melcher M. Leisch N. (2014). Nitrososphaera viennensis gen. nov., sp. nov., an aerobic and mesophilic, ammonia-oxidizing archaeon from soil and a member of the archaeal phylum *Thaumarchaeota*. *Int. J. Syst. Evol. Microbiol.* 64(Pt 8) 2738–2752. 10.1099/ijs.0.063172-0 24907263 PMC4129164

[B86] Thabet O. B. D. Gtari M. Sghaier H. (2017). Microbial diversity in phosphate rock and phosphogypsum. *Waste Biomass Valorization* 8 2473–2483. 10.1007/s12649-016-9772-1

[B87] Thavamani P. Samkumar R. A. Satheesh V. Subashchandrabose S. R. Ramadass K. Naidu R. (2017). Microbes from mined sites: harnessing their potential for reclamation of derelict mine sites. *Environ. Pollut.* 230 495–505. 10.1016/j.envpol.2017.06.056 28688926

[B88] Topalovic O. Hussain M. Heuer H. (2020). Plants and associated soil microbiota cooperatively suppress plant-parasitic nematodes. *Front. Microbiol.* 11:313. 10.3389/fmicb.2020.00313 32184773 PMC7058703

[B89] Trifi H. Najjari A. Achouak W. Barakat M. Ghedira K. Mrad F. (2020). Metataxonomics of *Tunisian phosphogypsum* based on five bioinformatics pipelines: insights for bioremediation. *Genomics* 112 981–989. 10.1016/j.ygeno.2019.06.014 31220587

[B90] Ullah I. Jilani G. Khan K. S. Akhtar M. S. Rasheed M. (2013). Phosphorous solubilization from phosphate rock by the interactive effect of Thiobacilli and elemental sulfur. *J. Agric. Res.* 51:03681157.

[B91] van Bruggen A. H. C. Goss E. M. Havelaar A. van Diepeningen A. D. Finckh M. R. Morris J. G. (2019). One health – cycling of diverse microbial communities as a connecting force for soil, plant, animal, human and ecosystem health. *Sci. Total Environ.* 664 927–937. 10.1016/j.scitotenv.2019.02.091 30769316

[B92] Vurukonda S. S. K. P. Vardharajula S. Shrivastava M. SkZ A. (2016). Enhancement of drought stress tolerance in crops by plant growth promoting rhizobacteria. *Microbiol. Res.* 184 13–24. 10.1016/j.micres.2015.12.003 26856449

[B93] Wang M. Tang Y. Anderson C. W. Jeyakumar P. Yang J. (2018). Effect of simulated acid rain on fluorine mobility and the bacterial community of phosphogypsum. *Environ. Sci. Pollut. Res.* 25 15336–15348. 10.1007/s11356-018-1408-5 29564699

[B94] Wu Z. Yu F. Sun X. Wu S. Li X. Liu T. (2018). Long term effects of *Lespedeza bicolor* revegetation on soil bacterial communities in Dexing copper mine tailings in Jiangxi Province, China. *Appl. Soil Ecol.* 125 192–201. 10.1016/j.apsoil.2018.01.011

[B95] Yadav A. K. Kumar N. Sreekrishnan T. R. Satya S. Bishnoi N. R. (2010). Removal of chromium and nickel from aqueous solution in constructed wetland: mass balance, adsorption-desorption and FTIR study. *Chem. Eng. J.* 160 122–128. 10.1016/j.cej.2010.03.019

[B96] Ye D. Li T. Yu H. Zou L. Huang H. Zhang X. (2020). Characteristics of bacterial community in root-associated soils of the mining ecotype of *Polygonum hydropiper*, a P-accumulating herb. *Appl. Soil Ecol.* 150:103477. 10.1016/j.apsoil.2019.103477

[B97] Zheng B. X. Bi Q. F. Hao X. L. Zhou G. W. Yang X. R. (2017). *Massilia phosphatilytica* sp. nov., a phosphate solubilizing bacteria isolated from a long-term fertilized soil. *Int. J. Syst. Evol. Microbiol.* 67 2514–2519. 10.1099/ijsem.0.001916 28853679

[B98] Zouch H. Karray F. Armougom F. Chifflet S. Hirschler-Réa A. Kharrat H. (2017). Microbial diversity in sulfate-reducing marine sediment enrichment cultures associated with anaerobic biotransformation of coastal stockpiled phosphogypsum (Sfax, Tunisia). *Front. Microbiol.* 8:1583. 10.3389/fmicb.2017.01583 28871244 PMC5566975

