# Supplementary material for: Description of Microbial Communities of Phosphate Mine Wastes in Morocco, a Semi-Arid Climate, Using High-Throughput Sequencing and Functional Prediction
Source: Front Microbiol. 2021 Jul 8;12:666936. doi: 10.3389/fmicb.2021.666936 (PMC8297565; doi:10.3389/fmicb.2021.666936)
Supplement: Supplementary file 1 [file Data_Sheet_1.docx]

Supplementary Material

# Supplementary Figures and Tables

## Supplementary Tables

**Supplementary Table S1**

***Supplementary Table S1.****OTUs abundance of raw and normalized data of different samples of phosphate mining wastes. Cells C1 and C4 are composed of phosphate waste rock, overburden material, removed to reach the phosphate ore. Cell 3 is composed of phosphate sludge, produced during the concentration processes. “BS” correspond for bare soils and “VS” for soils with plants.*

**Supplementary Table S2**

***Supplementary Table S2.****Distribution of identified genera in different samples of phosphate mining wastesbefore and after normalization. Cells C1 and C4 are composed of phosphate waste rock, overburden material, removed to reach the phosphate ore.Cell 3 is composed of phosphate sludge, produced during the concentration processes. “BS” correspondsto bare soils and “VS” to soils with plants.*

**Supplementary Table S3**

**Supplementary TableS3.** *Permutational multivariate analysis of variance (PERMANOVA) of microbialcommunities (genera level) evaluating the effect of type of soil (phosphate wastes rock and phosphate sludge), the effect of plant (VSreplicates indicating presence of annual plants and BSreplicates from bare soil) and their interaction based on Jaccard dissimilarity.*

|  | df | SumOfSqs | F | r^2^ | p-value |
| --- | --- | --- | --- | --- | --- |
| Soil | 1 | 0.63179 | 9.6032 | 0.29784 | 0.001 *** |
| Plant | 1 | 0.31021 | 4.7152 | 0.14624 | 0.001 *** |
| Soil x Plant | 1 | 0.25820 | 3.9247 | 0.12172 | 0.002 ** |
| Residual | 14 | 0.92105 |  | 0.43420 |  |
| Total | 17 | 2.12125 |  | 1.00000 |  |

The column F corresponds to the Fischer coefficient, df to the degrees of freedom, Sums of Sqs to the sum of squares, p-values are based on 999 permutations and r^2^values indicate the proportion of variation explained by each variable.

*0.01 < p < 0.05; **0.001 < p < 0.01; ***p < 0.001

**Supplementary Table S4**

***Supplementary Table S4.*** *The r^2^ correlation coefficient and the corresponding p-values corrected by “fdr” method of studied physicochemical parameters indicating the goodness of fit and significance determined by vector fitting to the NMDS. Signif. codes: 0 ‘***’ 0.001 ‘**’ 0.01 ‘*’ 0.05 ‘.’*

|  | **r^2^** | **p-value** |  |
| --- | --- | --- | --- |
| **SiO_2_** | 0.927 | 0.002 | ** |
| **Al_2_O_3_** | 0.007 | 0.943 |  |
| **Fe_2_O_3_** | 0.830 | 0.002 | ** |
| **CaO** | 0.389 | 0.026 | * |
| **MgO** | 0.729 | 0.002 | ** |
| **K_2_O** | 0.052 | 0.747 |  |
| **TiO_2_** | 0.346 | 0.044 | * |
| **P_2_O_5_** | 0.837 | 0.002 | ** |
| **pH** | 0.961 | 0.002 | ** |
| **TOC** | 0.883 | 0.002 | ** |
| **N** | 0.468 | 0.011 | * |

**Supplementary TableS5**

*Supplementary Table S5.Nearest Sequences Taxon Indexrelated to PICRUSt2 analyses*

| Sites | mean NSTI |
| --- | --- |
| C1BS | 0.06180149 |
| C1VS | 0.03912036 |
| C3BS | 0.0537916 |
| C3VS | 0.03509808 |
| C4BS | 0.05058919 |
| C4VS | 0.06499133 |

**Supplementary Table S6**

***Supplementary Table S6.****Majorplant growth traits of genera close to sequences retrieved in alkaline phosphate mining wastes of Morocco, a semi-arid area.*

## Supplementary Figures

**Supplementary Figure S1**


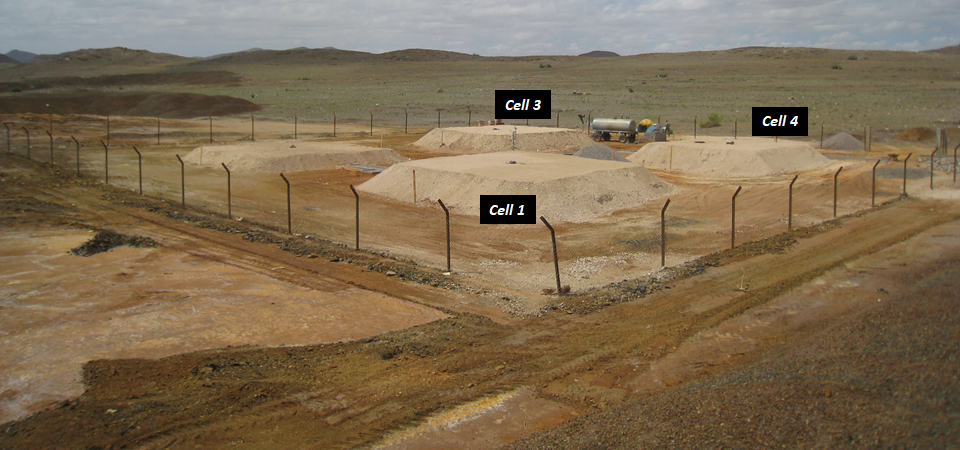


***Supplementary Figure S1.*** *View of the four cells at the end of the construction phase in 2011.*

**Supplementary Figure S2**


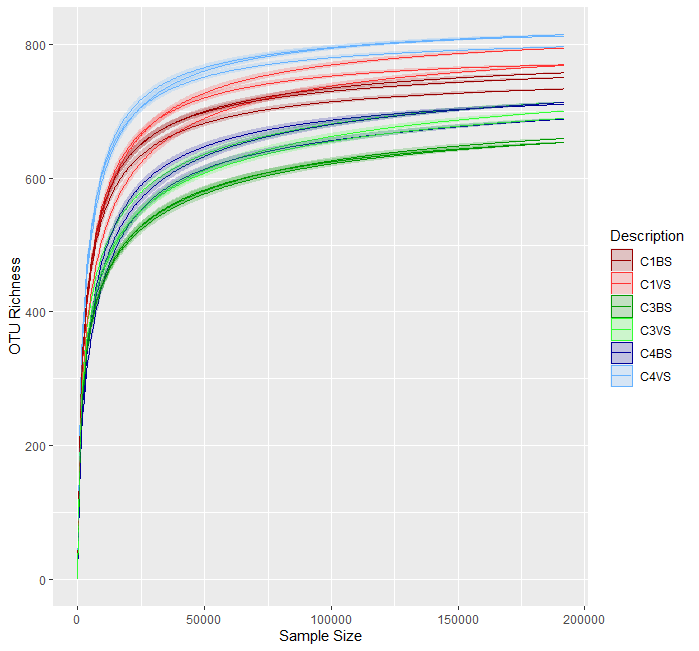


***Supplementary Figure S2.*** *Rarefaction curves of the microbial 16S rDNA sequences from phosphate mining wastes of the store-and-release cover of Kettara, based on OTUs calculated at 97% identity for the normalized data. The total number of sequences analyzed is plotted against the number of OTUs observed in the same library.*

**Supplementary Figure S3**


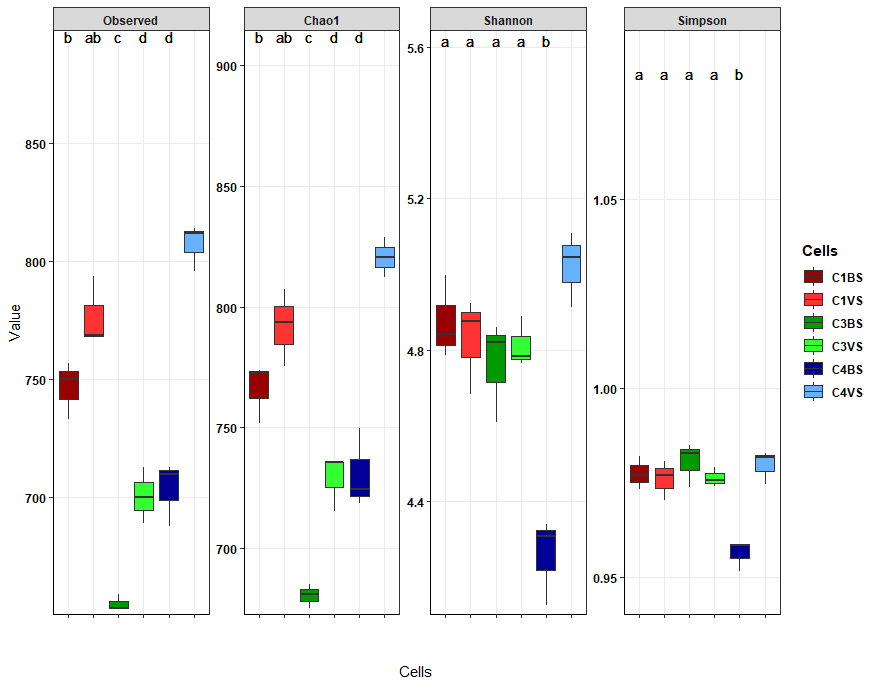


***Supplementary Figure S3.*** *Bacterial species richness in number of OTUs observed, Chao1, Shannon diversity index and Simpson diversity index in the alkaline phosphate mine wastes present in the experimental cells used as store-and-release cover of acidogenic pyrrhotite wastes. Error bars represent standard deviation of the mean value (analyses performed in triplicates).*

**Supplementary Figure S4**

******

***Supplementary Figure S4.*** *Network analysis illustrating the relationship between genera proven to be statistically different between bare soil (pink) and soil near plants roots (light green) based on ANOVA analysis. The edges represent the r^2^ correlation coefficient.*
