# Supplementary material for: Description of Microbial Communities of Phosphate Mine Wastes in Morocco, a Semi-Arid Climate, Using High-Throughput Sequencing and Functional Prediction
Source: Front Microbiol. 2021 Jul 8;12:666936. doi: 10.3389/fmicb.2021.666936 (PMC8297565; doi:10.3389/fmicb.2021.666936)
Supplement: Supplementary file 2 [file Data_Sheet_2.docx]

***Supplementary Table S6***

| **Genus** | **Activities** | | | | **Resistance/Tolerance/ Transformation of pollutants** | **Other** |
| --- | --- | --- | --- | --- | --- | --- |
|  | **IAA** | **Phosphate** | **Siderophores** | **N fixation** |  |  |
| ***Massilia*** | Becerra-Castro et al., 2012 ; Kuffner et al., 2010 ; Ofek et al., 2012 ; Zhu et al., 2014 | Zheng et al., 2017 ; Zheng et al., 2018 | Ofek et al., 2012 ; Singh et al., 2015 |  | Resistant to multiple metals like As, Cu, Sb, Zn, Ni, Pb, and Cd (Du et al., 2012 ; Feng et al., 2016)  As tolerant (Zhu et al., 2014) | Antagonistic activity towards *Phytophthora infestans* (Ofek et al., 2012; Weinert et al., 2010; Xiao et al., 2017)  Cellulose hydrolysis (Singh et al., 2015)  Chitin degradation (Adrangi et al., 2010; Chen et al., 2020 ; Faramarzi et al., 2009; Ofek et al., 2012) |
| ***Sphingomonas*** | Ali et al., 2018 ; Durand et al., 2018 ; He et al., 2010 ; Khan et al., 2014 ; Kuffner et al., 2010 ; Sun et al., 2010 ; Tsavkelova et al., 2007 ; Xu et al., 2018 ; Zhu et al., 2014 | Ali et al., 2018 ; Durand et al., 2018 ; He et al., 2010 ; Rincon-Molina et al., 2020 ; Zheng et al., 2018 | Durand et al., 2018 ; Sun et al., 2010 ; Xu et al., 2018 ; Zhu et al., 2014 | Ali et al., 2018 ; Etesami, 2019 ; Suleiman et al., 2019 ; Sun et al., 2014 ; Xu et al., 2018 | Resistant to Co, Zn, and Cd (Silver and Phung, 1996) and to Pb, Zn, Ni, and Cd (He et al., 2010)  High resistant to Zn, Cu, Pb and Ni (Sun et al., 2010; Xie et al., 2010)  Resistant to Cu (He et al., 2010), Cd (Luo et al., 2011), Sr (Ozer et al., 2013) and Hg (Durand et al., 2018) and to Al, Cu and Zn (Rincon-Molina et al., 2020)  Tolerant to Cd and Zn (Kuffner et al., 2010), As (Zhu et al., 2014) and Cr (Bilal et al., 2018) | Phytohormones production (gibberellins, salicylic acid, zeatin, abscisic acid, Khan et al., 2014 ; 2020)  ACC deaminase production (Asaf et al., 2020; Luo et al., 2011; Rincon-Molina et al., 2020)  Growth enhancement of tomato, bean and soybean (Khan et al., 2014; Rincon-Molina et al., 2020)  Corn root elongation (Ali et al., 2018)  Orchid seeds germination increasment (Tsavkelova et al., 2007  Zn solubilization (Ali et al., 2018)  Biocontrol activity and plant protection (antimicrobial and antifungal), symptoms suppression (Bulgarelli et al., 2013; Ali et al., 2018)  Cellulose, ligno and hemicellulose, xylene degradation (Asaf et al., 2020)  Cellulose degradation (Rincon-Molina et al., 2020)  Cd biosorption (Tangaromsuk et al., 2002)  Plant-growth improvement during stress conditions (drought, salinity and heavy metals, Asaf et al., 2020)  Growth improvment and stress tolerance of tomato under salinity and Cd stresses (Halo et al., 2015; Khan et al., 2014)  Exopolisacharides production (Rincon-Molina et al., 2020)  Degradation of organometallic compounds and Cd accumulation (Asaf et al., 2020) |
| ***Adhaeribacter*** |  |  |  |  |  | Cellulose degradation (Cotton et al., 2019)  Adhesiveness (McBride et al., 2014) and EPS production, organic carbon break-down and carbon dioxide production (Liu et al., 2014) |
| ***Hymenobacter*** |  | Dimitrijevic et al., 2018 ; Mihajlovski et al., 2020 |  | Dimitrijevic et al., 2018 |  | Ca solubilization (Dimitrijevic et al., 2018)  Cellulose hydrolysis (Mihajlovski et al., 2020) |
| ***Brevundimonas*** | Ali et al., 2018 ; Cavalca et al., 2010 ; Rana et al., 2011 ; Singh et al., 2016 ; Verma et al., 2015a ; Yu et al., 2014 | Ali et al., 2018 ; Rana et al., 2011 ; Singh et al., 2016 ; Verma et al., 2015a | Cavalca et al., 2010 ; Singh et al., 2016 | Ali et al., 2018 ; O’Donnell et al., 2019 | Resistant to As (Cavalca et al., 2010)  Tolerant to As (Singh et al., 2016 ; Zhu et al., 2014) | Gibberellins production (Verma et al., 2015a)  ACC deaminase production (Singh et al., 2016)  Corn root elongation (Ali et al., 2018)  Ammonia production (Rana et al., 2011 ; Verma et al., 2015a)  Biocontrol activity against *Fusarium* *graminearum*, *Rhizoctonia* *solani* and *Macrophomina* *phaseolina* (Verma et al., 2015a)  Cd adsorption (Robinson et al., 2001)  Hg accumulation in biofilm, decreasing Hg level in soil (Rijavec et al., 2019) |
| ***Cnuella*** |  |  |  |  | Tolerate up to 2% NaCl (Zhao et al., 2014) |  |
| ***Aggregicoccus*** |  |  |  |  |  | Aggregates formation (Sood et al., 2015) |
| ***Microvirga*** |  |  |  | Ardley et al 2012 ; Safronova et al., 2017 ; Veyisoglu et al., 2016 |  | Legumes nodulation such as *Lupinus* (Ardley et al 2012 ; Msaddak et al., 2017)  As oxidation (Tapase and Kodam 2018) |
| ***Rubellimicrobium*** |  |  |  |  |  | Amylase and protease production (Dastager et al., 2009) |
| ***Arthrobacter*** | Belimov et al., 2005 ; Dell’Amico et al., 2005 ; Durand et al., 2018 ; Fan et al., 2016 ; Xu et al., 2018 ; Gong et al., 2018 ; He et al., 2010 ; Kong and Glilck, 2017; Kuffner et al., 2010 ; Luo et al., 2011 ; Ma et al., 2011 ; Susilowati et al., 2015 ; Verma et al., 2014 ; Verma et al., 2015a ; Zhang et al., 2012 | Adnan et al., 2017 ; Çakmakçı, 2019 ; Fan et al., 2016 ; Hayat et al., 2010 ; Kong and Glilck, 2017;; Ma et al., 2011 ; Mitra et al., 2020 ; Pattnaik et al., 2019 ; Susilowati et al., 2015 ; Tchakounté et al., 2018 ; Verma et al., 2014 ; Verma et al., 2015a ; Xu et al., 2018 ; Zhang et al., 2012 ; Zheng et al., 2018 | Belimov et al., 2005 ; Gong et al., 2018 ; He et al., 2010 ; Kong and Glilck, 2017 ; Luo et al., 2011 ; Ma et al., 2011 ; Tchakounté et al., 2018 ; Verma et al., 2014 ; Verma et al., 2015a ; Zhang et al., 2012 | Ali et al., 2018 ; Çakmakçı, 2019 ; Fan et al., 2016 ; Sellstedt and Richau, 2013 ; Singh, 2018  Suleiman et al., 2019 ; Tchakounté et al., 2018 ; Verma et al., 2014 ; Verma et al., 2015a ; Xu et al., 2018 | Resistant to Ni, Co, Zn, Cd, Cu (Remenár et al., 2014)  Resistant to Hg (Durand et al., 2018), Cd (Luo et al., 2011; Xu et al., 2018), Cu (He et al., 2010), and Ni (Kong and Glick, 2017)  Tolerant to Pb, Cd, Zn and Ni (He et al., 2010)  Tolerant to Cd (Belimov et al., 2005), Zn and Ni resistant (Dell’Amico et al., 2005)  Tolerant to salt (Tchakounté et al., 2018) | Cytokinins and gibberellins production (Kong and Glick, 2017)  ACC deaminase production (Barnawal et al., 2014; Kuffner et al., 2010 ; Ma et al., 2011; Xu et al., 2018b)  Growth improvement of maize (Pereira and Castro 2014)  Root length promotion (Ali et al., 2018 ; He et al., 2010; Ma et al., 2011)  Rhizobial nodulation and mycorrhizal colonization enhancement (Barnawal et al., 2014; Del Carmen Orozco-Mosqueda et al., 2020)  Seed germination and seedling growth stimulation of millet (Goswami and Deka, 2020; Niu et al., 2018)  Zn solubilization (Verma et al., 2014 ; Verma et al., 2015a)  Ammonia production (Gong et al., 2018 ; Verma et al., 2014, 2015a)  Thiosulfate oxidation (Siddikee et al., 2010)  In vitro antagonistic activity (Weinert et al., 2010) and nematode biocontrol mecanisms (Topalovic et al., 2020)  Antibiotics production (Adegboye and Babalola, 2016)  Cellulose (Gong et al., 2018 ; Susilowati et al., 2015;Tang et al., 2012), hemicellulose and lignin degradation (Tang et al., 2012)  Salt stress alleviation in tomato (Fan et al., 2016) and growth promotion of *Triticum aestivum* under Cr and salt stress (Naseem et al 2016)  Plant seedling and root growth improvement of *Solanum* *nigrum* under Cd stress (Xu et al., 2018)  Oxidize As (Bachate et al., 2013 ; Prasad et al., 2009) |
| ***Skermanella*** |  |  |  | Luo et al., 2012a ; Navarro-Noya et al., 2010 ; Sly et al., 1999 | Highly Sb resistant (Luo et al., 2012a) | Biocontrol activity protecting rice implicating larvicidal potential (Panneerselvam et al., 2018)  Cellulose degradation (Weon et al., 2007) |
| ***Blastocatella*** |  |  |  |  | Highly tolerant to Cd, Zn, Pb and Hg (Guo et al., 2017) | Cellulose degradation (Foesel et al., 2013 ; Weon et al., 2007)  Chitin degradation (Foesel et al., 2013) |
| ***Lysobacter*** | Lasudee et al., 2017 | Çakmakçı, 2019; Lasudee et al., 2017 | Lasudee et al., 2017 | Çakmakçı, 2019 ; Iwata et al., 2010 ; Xu et al., 2018 | Resistant to Sb, Cu (Shi et al., 2013) and As (Luo et al., 2012b) | Growth promotion and seed germination of *Vigna sp.* (Lasudee et al., 2017)  Ammonia accumulation (Iwata et al., 2010)  Important biocontrol activities (Palumbo et al., 2005; Park et al., 2008; Zhou et al., 2016), and nematode biocontrol mechanisms (Hu et al., 2017)  In vitro antagonistic activity (Folman et al., 2003; Weinert et al., 2010)  Lyse a variety of bacteria, fungi, yeasts, algae and nematodes (Folman et al., 2003)  Antifungal activities (Kilic-Ekici et al., 2004) and suppression of damping-off disease in plants (Islam et al., 2005) |
| ***Noviherbaspirillum (****reclassification of Herbaspirillum soli, H. aurantiacum, H.canariense and H. psychrotolerans, Lin et al., 2013)* | *Herbaspirillum* : Bastián et al., 1998 ; Moreira et al 2016 ; Radwan et al., 2002 ; Sbabou et al., 2016 |  |  | *Herbaspirillum* : Baldani et al., 2000 ; Etesami, 2019 ; Neiverth et al., 2014 ; Park et al., 2005 ; Ramakrishna et al., 2019 | *Herbaspirillum* : resistant to As, Zn, Cu and Pb (Govarthanan et al., 2014)  High tolerace to Zn and Pb Sbabou et al., 2016) | *Herbaspirillum* : growth promotion of rice (Wang et al., 2014)  Zn toxicity symptoms suppression and plant growth enhancement of *Hedysarum* (Sbabou et al., 2016) |
| ***Arcticibacter*** |  |  |  |  |  | Cellulose degradation (Prasad et al., 2013) |
| ***Devosia*** | Rashid et al., 2012 | Nor et al., 2017 | Rashid et al., 2012 | Rivas et al., 2002 ; 2003 |  | ACC deaminase production (Rashid et al., 2012)  Root nodules formation in some legumes (Rivas et al., 2002)  Ammonnia production (Rashid et al., 2012)  Nematode biocontrol mecanisms (Topalovic et al., 2020) |
| ***Thermithiobacillus*** |  |  |  |  |  | Sulfur oxidizing bacterium (Watanabe et al., 2016) |
| ***Leptolyngbya*** |  |  |  |  |  | Complex EPS production and biocrust formation, aggregating sand, chelating nutrients and maintaining hydratation (Mugnai et al., 2018) |
| ***Pontibacter*** | Dastager et al., 2010 | Dastager et al., 2010 | Dastager et al., 2010 | Xu et al., 2014 |  | ACC deaminase production (Dastager et al., 2010)  Ca solubilizer (Dastager et al., 2010) |
| ***Pseudomonas*** | Aliyat et al., 2020 ; Ambrosini et al., 2012 ; Del Carmen Orozco-Mosqueda et al., 2020 ; Goswami et al., 2016 ; Dell'Amico et al., 2005 ; Durand et al., 2018 ; Gupta and Pandey, 2019; Hernández-León et al., 2015 ; Kaushal and Wani, 2016; Pattnaik et al., 2019 ; Rajkumar and Freitas, 2008; Sarma and Saikia, 2014 ; Tahir et al., 2019 ; Yahaghi et al., 2019 | Ambrosini et al., 2012 ; Das et al., 2014 ; Del Carmen Orozco-Mosqueda et al., 2020 Goswami et al., 2016 ; Durand et al., 2018 ; Egamberdiyeva, 2007 ; Kaushal and Wani, 2016; Mitra et al., 2020 ; Pattnaik et al., 2019 ; Rajkumar and Freitas, 2008; Ramakrishna et al., 2019 ; Sarma and Saikia, 2014; Sbabou et al., 2016 ; Zahir et al., 2009 ; Zheng et al., 2018 | Aliyat et al., 2020 ; Ambrosini et al., 2012 ; Del Carmen Orozco-Mosqueda et al., 2020 ; Goswami et al., 2016 ; Dell'Amico et al., 2005 ; Durand et al., 2018 ; Hernández-León et al., 2015 ; Kaushal and Wani, 2016 ; Pattnaik et al., 2019 ; Sarma and Saikia, 2014 ; Yahaghi et al., 2019 | Durand et al., 2018 ; Egamberdiyeva, 2007 ; Park et al., 2005 | Resistant to many metals: Sr (Ozer et al., 2013), Cd, Zn, Co and Ni (Dell'Amico et al., 2005), Hg (Durand et al., 2018), Cu (Ramakrishna et al., 2019), Ni, Cu and Zn (Rajkumar and Freitas, 2008), Pb (Kannojia et al., 2019 ; Sheng et al., 2008), Zn and Pb (Sbabou et al., 2016)  Very high As resistance (Das et al., 2014)  Tolerant to F (Grifoll et al., 1995, Wang et al., 2018a), Cd and Zn (Kuffner et al., 2010) | Phytohormone production: cytokinins (Goswami et al., 2016), and gibberellins (Kong and Glick, 2017)  ACC deaminase production (Chinnaswamy et al., 2018 ; Hernández-León et al., 2015; Kaushal and Wani, 2016; Saravanakumar and Samiyappan, 2007; Sarma and Saikia, 2014; Tahir et al., 2019)  Plant growh improvement (Del Carmen Orozco-Mosqueda et al., 2020; Goswami et al., 2016)  Chlorophyll content regulation (Han et al., 2014; Kaushal and Wani, 2016)  Nodulation increase (Chinnaswamy et al., 2018; Del Carmen Orozco-Mosqueda et al., 2020)  Ammonia production (Kaushal and Wani, 2016; Sbabou et al., 2016)  Potassium solubilization (Egamberdiyeva, 2007 ; Pattnaik et al., 2019)  Sulfur oxidation (Trifi et al., 2020)  Plant diseases reduction caused by many phytopathogens comprising bacteria and fungi (Berg et al., 2000; Pandey et al., 2006; Weinert et al., 2010) ; many in vitro antagonistic activity (Mazzola et al., 1995; 2007; Mitra et al., 2020 ; Schouten et al., 2004; Santoyo et al., 2012), systematic resistances induction against phytopathogenes (Goswami et al., 2016 ; Santoyo et al., 2012) and antibiotic production (Santoyo et al., 2012), hydrolytic enzymes excretion (Del Carmen Orozco-Mosqueda et al., 2020 ; Goswami et al., 2016; Pandey et al., 2006)  Nematode biocontrol mecanisms (Topalovic et al., 2020)  Drought resistance induction of many plants (Goswami et al., 2016; Kannojia et al., 2019; Sandhya et al., 2010) by EPS production, increasing soil aggregation and water uptake, Vardharajula et al. 2009), by upregulation of drought stress responsive-genes such as superoxide dismutase (Kaushal and Wani, 2016; Sarma and Saikia, 2014)  Soil aggregation improvement by Exopolysaccharides (EPS) production during drought stress (Sandhya et al. 2009)  Exopolysaccharides production under drought and salinity stresses (Kaushal and Wani, 2016; Sandhya et al., 2009; Tewari and Arora, 2014; Vardharajula et al. 2009)  Plant resistance induction to many environmental stresses such as drought and salinity or heat (Santoyo et al., 2012)  Saline resistance promotion of many plants (Kannojia et al., 2019; Saravanakumar and Samiyappan, 2007) by biofilm formation and tolerance to salt (Del Carmen Orozco-Mosqueda et al., 2020) or by nutrient uptake increasement under salinity stress (Han et al., 2014; Kaushal and Wani, 2016)  Heat resistance promotion (Ali et al., 2009 ; Kannojia et al., 2019)  Osmoprotectants production (Dell'Amico et al., 2005; Del Carmen Orozco-Mosqueda et al., 2020; Rajkumar and Freitas, 2008)  Zn toxicity symptoms suppression and plant growth enhancement of *Hedysarum* (Sbabou et al., 2016)  As oxidation (Biswas and Sakar 2018; Das et al., 2014) |
| ***Pedobacter*** | Ali et al., 2018; Pereira and Castro 2014 | Ali et al., 2018; Pereira and Castro 2014 | Ali et al., 2018; Pereira and Castro 2014 | Ali et al., 2018 ; Padda et al., 2018 |  | Corn root elongation (Ali et al., 2018)  Zn solubilization (Ali et al., 2018)  Antifungal antagonism activity against *F. oxysporum*, *P. ephenidermattum* and *V. dahliae* (Ali et al., 2018) |
| ***Gemmatimonas*** |  |  |  |  |  | Plant parasitic nematodes suppression (Elhady et al., 2017)  Polyphosphate accumulation (Zhang et al., 2003) |
| ***Peredibacter*** |  |  |  |  |  | Predatory bacteria grazing Gram negative bacteria (Davidov and Jurkevitch, 2004) |
| ***Rufibacter*** |  |  |  |  | Radiation resistance (Kim and Srinivasan, 2020; Zhang et al., 2015). | Nitrate reduction (Liu et al., 2016) |
| ***Tychonema*** |  |  |  |  |  | Strong inhibitory effect against the algae *Microcystis aeruginosa* (Shao et al 2013) |
| ***Altererythrobacter*** |  | Ali et al., 2018 |  | Ali et al., 2018 | Up to 6% NaCl resistant (Kwon et al., 2007) |  |
| ***Bdellovibrio*** |  |  |  |  |  | Biocontrol agent: predation of pathogen of cultured mushrooms *Pseudomonas tolaasii* (Saxon et al., 2014)  Predation of other Gram-negative preys (Davidov and Jurkevitch, 2004; Hassani et al., 2018) |
| ***Nocardioides*** | Gong et al., 2018 |  | Gong et al., 2018 |  | Resistant to dessication in arid area (Mohammadipanah and Wink, 2016) | ACC deaminase production (Gong et al., 2018)  In vitro antagonistic activity (Weinert et al., 2010)  Plants protection (Coombs et al., 2004)  Antibiotic production (Dellweg et al., 1988) and fungal pathogens control (Coombs et al., 2004; Sun et al., 2014)  Hydrolytic enzymes production (Dastager et al., 2009), such as cellulase, amylase, chitinase, xylanase and protease (Gong et al., 2018)  As transformation (Bagade et al., 2016)  Organotrophic genera (Li et al., 2015) |
| ***Blastococcus*** | Li et al., 2008 |  |  | Adegboye and Babalola, 2012; Cobo-Díaz et al., 2015 | Resistant to Pb, As (Gtari et al., 2012), and U (Rastogi et al., 2010)  Thermophilic bacteria (Sar et al., 2013) | Nitrate to nitrite reduction (Castro et al., 2018) |
| ***Roseomonas*** | Kalam et al., 2017 ; Zhu et al., 2014 | Çakmakçı, 2019 ; Tchakounté et al., 2018 | Kalam et al., 2017 ; Tchakounté et al., 2018 | Çakmakçı, 2019 | Tolerance to As (Zhu et al., 2014) | ACC deaminase production (Kalam et al., 2017)  Pronounced PGP effect on tomato and black gram (Kalam et al., 2017)  As transformation (Bagade et al., 2016) |
| ***Pseudorhizobium*** |  |  |  |  |  | Nitrate reduction (Kimes et al., 2015) |
| ***Sphingoaurantiacus*** |  |  |  |  |  | Nitrate reduction (Tan et al., 2016) |
| ***Flavobacterium*** | Ali et al., 2018 ; Verma et al., 2015a ; Youseif, 2018 ; Youseif, 2018 | Etesami, 2019 ; Mitra et al., 2020 ; Moharana et al., 2018 ; Verma et al., 2015a ; | Belimov et al., 2005 ; Hayat et al., 2010 ; Kuffner et al., 2010 ; Verma et al., 2015a | Akhtar and Siddiqui, 2009 ; Ali et al., 2018 ; Gholami et al., 2009 ; Giri and Pati, 2004 ; Kolton et al., 2016 ;  Mitra et al., 2020 ; Singh, 2018 ; Xu et al., 2018 ; Youseif, 2018 | Cd resistant (Luo et al., 2011)  Tolerant to Cd (Kuffner et al., 2008), Cd and Zn (Kuffner et al., 2010) | Gibberellins production (Kong and Glick, 2017; Soltani et al., 2010)  ACC deaminase production (Belimov et al., 2005; Kolton et al., 2016; Verma et al., 2015a)  Root elongation (Ali et al., 2018)  Shoot and root fresh and dry biomass promotion of maize plants (Youseif, 2018*)*  Zn solubilization (Ali et al., 2018 ; Verma et al., 2015a)  Ammonia production (Verma et al., 2015a ; Youseif, 2018)  In vitro antagonistic activity (Weinert et al., 2010)  Biocontrol activity against *Phytophthora* and the fungus *Verticillium* *dahlia* (Kolton et al., 2016; Soltani et al., 2010)  Zn and Cd immobilization (Ma et al., 2011) |
| ***Chthoniobacter*** |  |  |  | Sun et al., 2014 |  |  |
| ***Belnapia*** |  |  |  |  |  | Nitrate reduction (Reddy et al., 2006) |
| ***Truepera*** |  |  |  |  | Radio tolerant (Albuquerque et al., 2005; Ivanova et al., 2011 ; Li et al., 2014)  Highly Ni-resistant (Remenár et al., 2017) | Extremophiles microorganism (Li et al., 2015) |
| ***Cellvibrio*** | Abd El-Azeem et al., 2007 | Abd El-Azeem et al., 2007 | Abd El-Azeem et al., 2007 |  |  | Corn root elongation (Ali et al., 2018) |
| ***Phenylobacterium*** | Ali et al., 2018 |  |  | Ali et al., 2018 ; Sun et al., 2014 |  |  |
| ***Paenibacillus*** | Ali et al., 2018 ; Bal et al., 2013 ; Das et al., 2014 ; Del Carmen Orozco-Mosqueda et al., 2020 ; Gupta and Pandey, 2019 ; Herrera et al., 2020a ; Kaushal and Wani, 2016; Upadhyay et al., 2011 ; Verma et al., 2014 ; Yahaghi et al., 2019 ; Yu et al., 2014 | Çakmakçı, 2019; Del Carmen Orozco-Mosqueda et al., 2020 ; Durand et al., 2018; Gupta and Pandey, 2019; Hayat et al., 2010 ; Kaushal and Wani, 2016; Mitra et al., 2020 ; Tchakounté et al., 2018 ; Upadhyay et al., 2011 ; Verma et al., 2014 | Bal et al., 2013 ; Das et al., 2014 ; Del Carmen Orozco-Mosqueda et al., 2020; Gupta and Pandey, 2019; Tchakounté et al., 2018 ; Verma et al., 2014 ; Yahaghi et al., 2019 | Ali et al., 2018 ; Anand et al., 2013 ; Çakmakçı, 2019 ; Figueiredo et al., 2008 ; Goswami et al., 2016 ; Puri et al., 2016 ; Tchakounté et al., 2018 ; Verma et al., 2014 | Hg resistant (Durand et al., 2018)  Very high As resistance (Das et al., 2014) | Phytohormones production: cytokinins (Bhattacharyya and Jha, 2012 ; Pattnaik et al., 2019) and gibberellins (Gupta and Pandey, 2019; Pattnaik et al., 2019; Verma et al., 2014)  ACC deaminase production (Bal et al., 2013 ; Das et al., 2014; Gupta and Pandey, 2019 ; Verma et al., 2014)  Plant health promotion by activating antioxidant, defense related proteins and phytohormones production (Ramakrishna et al., 2019)  Corn root elongation (Ali et al., 2018)  Ammonia production (Bal et al., 2013 ; Verma et al., 2014)  Nitrate reduction (Bal et al., 2013)  Potassium solubilization (Pattnaik et al., 2019; Verma et al., 2014)  Zn solubilization (Yahaghi et al., 2019; Verma et al., 2014)  In vitro antagonistic activity (Weinert et al., 2010)  Antifungal and antimicrobial activity (Compant et al., 2005, Goswami et al., 2016; Herrera et al., 2020a; Shi et al., 2017; Verma et al., 2014)  Cellulose degradation (Delavat et al., 2012 ; Maki et al., 2009)  Drought stress alleviation of *Arabidopsis* (Kaushal and Wani, 2016; Timmusk and Wagner, 1999)  Salinity stress alleaviation (Tchakounté et al., 2018) by EPS production and proline production (Kaushal and Wani, 2016; Upadhyay et al., 2011) |
| ***Geodermatophilus*** |  |  |  | Adegboye and Babalola, 2012 | Resistant to desiccation, gamma- and UV- radiation (Mohammadipanah and Wink, 2016)  Pb resistant (Gtari et al., 2012) |  |
| ***Thiobacillus*** |  | Alori et al., 2017 ; Raj et al., 2014 ; Moharana et al., 2018 ; Postma et al., 2010 |  | Huang et al., 2016; Liu et al., 2019; Yamanaka, 1996 |  | Sulfur oxidation (Bosch et al., 2012 ; Huang et al., 2016; Liu et al., 2019 ; McNeil et al., 2020 ; Yamanaka, 1996)  Carbone fixation (Huang et al., 2016; Liu et al., 2019 ; Yamanaka, 1996)  Ferrous iron oxidation (Huang et al., 2016; Liu et al., 2019; Nicolle et al., 2009 ; Yamanaka, 1996)  Typical extremophiles and lithotrophs (Li et al., 2015) |
| ***Marmoricola*** |  |  |  |  |  | Amylase and protease production (Dastager et al., 2009)  Nitrate reduction (Dastager et al., 2008) |
| ***Dyadobacter*** |  | Zhang et al., 2012 |  | Kumar et al., 2018 |  | Plant growth promotion of different crops (millet, mung bean, pea, etc., Kumar et al., 2018) |
| ***Methylibium*** |  |  |  |  |  | ACC deaminase production (Santiago et al., 2017)  Promote growth of potato seedlings (Santiago et al., 2017)  Hydrolytic enzymes production: cellulase, glucanase, chitinase, or protease (Santiago et al., 2017) |
| ***Parviterribacter*** |  |  |  | Padda et al., 2018 |  |  |
| ***Paracoccus*** | Sahoo et al., 2019 | Çakmakçı, 2019 |  | Çakmakçı, 2019 ; Sahoo et al., 2019 |  |  |
| ***Pseudoxanthomonas*** | Castellano-Hinojosa et al., 2016 ; Youseif, 2018 | Castellano-Hinojosa et al., 2016 ; Youseif, 2018 | Castellano-Hinojosa et al., 2016 ; Youseif, 2018 | Castellano-Hinojosa et al., 2016 ; Youseif, 2018 |  | ACC deaminase production (Castellano-Hinojosa et al., 2016)  Shoot and root fresh and dry biomass promotion of maize plants (Youseif, 2018*)*  Ammonia production (Youseif, 2018*)*  Reduce nitrite to nitrous oxide (Finkmann et al., 2000) |
| ***Chryseobacterium*** | Ali et al., 2018 ; Ambrosini et al., 2012 ; Farina et al., 2012 ; Herrera et al., 2020a ; Herrera et al., 2020b ; Luo et al., 2011 ; Marques et al., 2010 ; Rincon-Molina et al., 2020 | Ali et al., 2018 ; Çakmakçı, 2019 ; Herrera et al., 2020a ; Herrera et al., 2020b ; Rincon-Molina et al., 2020 | Ali et al., 2018 ; Ambrosini et al., 2012 ; Herrera et al., 2020b ; Luo et al., 2011 ; Marques et al., 2010 ; Rincon-Molina et al., 2020 | Ali et al., 2018; Çakmakçı, 2019 ; Xu et al., 2018 | Cd, Zn, Pb and Cu tolerance (Luo et al., 2011) | ACC deaminase production (Marques et al., 2010  Plant growth promotion of many plants such as sunflower (Ambrosini et al., 2012); bean by helping N and P uptake (Rincon-Molina et al., 2020), the Cd-hyperaccumulator *Solanum nigrum* (Luo et al., 2011) or the horsegram by increasing P content, reducing nitrate and increasing the chlorophyll content (Singh et al., 2013)  Corn root elongation (Ali et al., 2018)  Ammonia production (Marques et al., 2010)  Zn solubilization (Ali et al., 2018)  Antifungal activity (Ali et al., 2018; Etesami, 2019; Herrera et al., 2020a, 2020b)  Exopolysaccharides production (Rincon-Molina et al., 2020) |
| ***Lechevalieria*** |  |  |  |  | Resistante to dessication and high salinity (Mohammadipanah and Wink, 2016) | Antioomycete and antifungal activity against phytopathogenic *Phytophthora* and *Botrytis* and the yeast *Saccharomyces* (Lee et al., 2004) |
| ***Allorhizobium-Neorhizobium-Pararhizobium-Rhizobium*** | *Rhizobium* : Ambrosini et al., 2012 ; Kong and Glilck, 2017; Kuffner et al., 2010 ; Ma et al., 2011 ; Pattnaik et al., 2019; Solanki et al., 2017 ; Tsavkelova et al., 2007 ; Yu et al., 2014 | *Rhizobium* : Ambrosini et al., 2012 ; Goswami et al., 2016; Kong and Glilck, 2017; Mitra et al., 2020 ; Moharana et al., 2018 ; Pattnaik et al., 2019 ; Ramakrishna et al., 2019 ; Sbabou et al., 2016 ; Solanki et al., 2017 ; Zheng et al., 2018 | *Rhizobium* : Ambrosini et al., 2012 ; Ma et al., 2011 ; Pattnaik et al., 2019 ; Solanki et al., 2017 ; Yu et al., 2014 | *Rhizobium* : de Lajudie et al., 1998 ; Goswami et al., 2016 ; Gray and Smith, 2005;  Ma et al., 2011; Pattnaik et al., 2019 ; Solanki et al., 2017 ; Sun et al., 2014 ; Xu et al., 2018  Allorhizobium : Mitra et al., 2020 | *Rhizobium* : Cd and Zn tolerant (Kuffner et al., 2010  Ni, Cd, Cr, Pb, Zn and Cu resistant (Kong and Glilck, 2017)  High tolerance to Zn and Pb (Sbabou et al., 2016) | *Rhizobium* : phytohormones production: cytokinins and gibberellins (Goswami et al., 2016; Kong and Glick, 2017)  ACC deaminase production (Del Carmen Orozco-Mosqueda et al., 2020 ; Solanki et al., 2017)  Corn root elongation (Ali et al., 2018)  Form root nodules with legumes (Rivas et al., 2002)  Ammonia production (Kong and Glilck, 2017; Sbabou et al., 2016)  Antifungal antagonism against *F. oxysporum*, *R. solani* and *V. dahlia* in corn plant (Ali et al., 2018)  Nematode biocontrol mecanisms (Topalovic et al., 2020)  Sunflower protection and soil aggregation improvement under drought stress by exopolysaccharides production (Alami et al., 2000; Kaushal and Wani, 2016)  Drought stress alleviation by helping N uptake by sunflower plants (Alami et al., 2000)  Decrease concentration and toxicity of Ni and Zn (Ma et al., 2011)  *Allorhizobium:* form root nodules with *Neptunia natans* (de Lajudie et al., 1998; Gray and Smith, 2005)) |
| ***Novosphingobium*** | Ambrosini et al., 2012 |  | Ambrosini et al., 2012 |  | Resistant to Ni (Remenár et al., 2017) | Biocontrol of fungus in pepper (Sang et al., 2013)  Salt stress alleviation in association with *Pseudomonas putida* and plant growth promotion of citrus by decrease of transpiration rate and stomatal conductance with significant drop in levels of abscisic acid and salicylic acid (Goswami and Deka, 2020; Vives-Peris et al., 2018) |
| ***Deinococcus*** | Guerrero-Zúñiga et al., 2020 |  |  |  | Radiation tolerant (Li et al., 2015)  Growth in presence of radiation and Hg, Cd and Hg volatilization (Brim et al., 2000)  Reduction of Hg, Fe, U, Cr, Tc (Brim et al., 2003; Thatoi et al., 2014)  As resistant (Suresh et al 2004) | Extremophiles (Li et al., 2015) |
| ***Conexibacter*** |  |  |  |  |  | Nitrate reduction (Monciardini et al., 2003) |
| ***Candidatus*** | Ali et al., 2018 |  |  |  |  | Antifungal activity againt *Fusarium oxusporom* on corn plant (Ali et al., 2018) |
| ***Azospirillum*** | Ambrosini et al., 2012 ; Bhattacharyya and Jha, 2012 ; Abd El-Azeem et al., 2007 ; Fukami et al., 2018 ; Hayat et al., 2010 ; Rivera et al., 2018 | Abd El-Azeem et al., 2007 ; Mitra et al., 2020 ; Ramakrishna et al., 2019 | Ambrosini et al., 2012 ; Abd El-Azeem et al., 2007 | Fukami et al., 2018 ; Hayat et al., 2010 ; Mirza et al., 2001 ; Mitra et al., 2020 ; Pattnaik et al., 2019 ; Ramakrishna et al., 2019 ; Rodrigues et al., 2008 |  | Phytohormone production: cytokinins and gibberellin (Goswami et al., 2016; Kong and Glick, 2017), ethylene (Pattnaik et al., 2019), abscisic acid, salicylic acid and auxin (Fukami et al., 2018)  Remarkable capacity to promote plant growth (Ambrosini et al., 2012; Fukami et al., 2018; Hungria et al., 2010; Rincon-Molina et al., 2020)  Marked effects on the seedlings of corn, wheat, sorghum, and other grasses by improving total nitrogen requirements (Pathak and Kumar, 2016)  N and P uptake induction (Rincon-Molina et al., 2020)  Systemic Acquired Resistance (Fukami et al., 2018)  Mechanisms of tolerance of biotic and abiotic stresses (including Induced Systemic Resistance and drought stress tolerance and resistance induction of different plants (Dimkpa et al., 2009; Kannojia et al., 2019) by abccissic acid increase in *A. thialana* retarding water loss (Cohen et al., 2015; Kaushal and Wani, 2016), by proline production involved in osmotic regulation (Cohen et al., 2015; Kaushal and Wani, 2016) or by apoplastic water fraction increase in wheat (Creus et al., 2004; Goswami and Deka, 2020)  Biofilm formation (Kasim et al., 2016)  Salinity stress alleviation by Na concentration increase and K^+^/Na^+^ ratio and calcium concentration decrease (Goswami and Deka, 2020; Hamdia et al., 2004) or root branching enhancement and secretion of flavonoids and lipochitooligosaccharides increase (Goswami and Deka, 2020) |
| ***Promicromonospora*** | Gong et al., 2018 | Kang et al., 2012 |  |  | NaCl tolerance (Gong et al., 2018) | Gibberellins production (Kang et al., 2012)  ACC deaminase production (Gong et al., 2018)  Increased shoot and root growth, chlorophyll content stomatal closure to minimize water loss (Kang et al., 2014)  Ammonia production (Gong et al., 2018)  In vitro antagonistic activity (Weinert et al., 2010)  Antimicrobial activities inhibiting mycogone (Zhao et al., 2018)  Antibiotics production (Adegboye and Babalola, 2016)  Down-regulation of abscisic acid, a plant stress hormone (Kang et al., 2012  Hydrolytic enzymes production (chitinase, amylase, xylanase production and protease, Gong et al., 2018)  Cellulase production (Gong et al., 2018)  Significantly plants growth improvment inhibiting by high NaCl (200 mM) stress (Orhan and Demirci, 2020) |
| ***Streptomyces*** | Abdallah et al., 2019 ; Anwar et al., 2016 ; Borah and Thakur, 2020; Del Carmen Orozco-Mosqueda et al., 2020; Gangwar et al., 2014; Gong et al., 2018 ; Hamdali et al., 2008 ; Hamedi et al., 2015; Nimnoi et al., 2010 ; Passari et al., 2015 ; Solanki et al., 2017 ; Suksaard et al., 2017 | Anwar et al., 2016 ; Borah and Thakur, 2020; Gangwar et al., 2014 ; Gong et al., 2018; Hamdali et al., 2008 ; Jog et al., 2012 ; Moharana et al., 2018 ; Olanrewaju and Babalola, 2019; Passari et al., 2015 ; Pirhadi et al., 2018 ; Purushotham et al., 2018 ; Suksaard et al., 2017; Tchakounté et al., 2018 ; Zheng et al., 2018 | Anwar et al., 2016 ; Borah and Thakur, 2020; de Dimkpa et al., 2009 ; Gangwar et al., 2014 ; Gong et al., 2018 ; Hamdali et al., 2008 ; Hastuti et al., 2012 ; Hayat et al., 2010 ; Jog et al., 2012 ; Kuffner et al., 2008 ; Olanrewaju and Babalola, 2019; Passari et al., 2015 ; Pattnaik et al., 2019; Purushotham et al., 2018; Rungin et al., 2012 ; Solanki et al., 2017 ; Suksaard et al., 2017 | Borah and Thakur, 2020; Li et al., 2012 ; Olanrewaju and Babalola, 2019; Park et al., 2005 ; Pirhadi et al., 2018; Sellstedt and Richau, 2013 ; Solanki et al., 2017 ; Susilowati et al., 2015 | High temperature, salinity, desiccation and pressure resistance (Mohammadipanah and Wink, 2016)  Resistant to Sr (Kamala et al., 2019), Ni, Co, Zn, Cd, Cu (Remenár et al., 2014), Zn, Cd and Pb (Kuffner et al., 2008), and Ni, Cd, Cu and Zn (Hamedi et al., 2015)  Cd and Zn tolerant (Kuffner et al., 2010)  Salt tolerance (Tchakounté et al., 2018) | Phytohormones production: auxins, cytokinins, gibberellins, brassinolide, salicylic acid, jasmonic acid, serotonin and abscisic acid (Hamedi et al., 2015; Olanrewaju and Babalola, 2019)  ACC deaminase production (Anwar et al., 2016 ; Del Carmen Orozco-Mosqueda et al., 2020 ; El-Tarabily et al., 2010 ; Gong et al., 2018 ; Kuffner et al., 2010 ; Suksaard et al., 2017)  Growth enhancement of many plants such as wheat (Hamdali et al., 2008), soybean (Wahyudi et al., 2019), chili (Nimnoi and Pornthip Ruanpanun, 2020) or rice (Suksaard et al., 2017)  Ammonia production (Anwar et al., 2016; Borah and Thakur, 2020; Passari et al., 2015)  Many antifungal activities against *Fusarium fujikuroi ; F. oxysporum ; Magnaporthe oryzae ; Pyricularia oryzae ; P. Grisea ; Rhizoctonia solani ; R. oryzae-sativae ; Bipolaris oryzae ; Curvularia oryzae ; Alternaria sp.*and *Sclerotium sp.* (Etesami, 2019; Weinert et al., 2010)  Reduce plant diseases of many pathogen (Berg et al., 2000; Borah and Thakur, 2020; Olanrewaju and Babalola, 2019; Weinert et al., 2010)  Nematode biocontrol mecanisms (Ryan et al., 2009; Topalovic et al., 2020)  High metabolites production acting as antibiotic, antibacterial, antimicrobial, antiparasitic, pesticide, antihelminthic, antifungal (Compant et al., 2005; Goswami et al., 2016; Hamdali et al., 2008; Hassani et al., 2018; Olanrewaju and Babalola, 2019)  Hydrolitic enzymes production: chitinases (Hastuti et al., 2012; Passari et al., 2015), proteases, lipases, nucleases, and elastases (Ryan et al., 2009)  Cellulose production (Susilowati et al., 2015)  Cell wall degrading enzymes (Minamiyama et al., 2003)  Rice growth promotion under salinity stress (Suksaard et al., 2017)  Biosorption of Cd, Cr, Pb, Cu, Ni and Zn and bioaccumulation of Cd, Cr, Cu and Zn (Alvarez et al., 2017)  Able to induce biosorption of Sr ion radionuclide in EPS (Kamala et al., 2020) |
| ***Anaeromyxobacter*** |  |  |  |  |  | Iron reduction (Wang et al., 2020) |
| ***Stenotrophomonas*** | Abdallah et al., 2019 ; Ali et al., 2018 ; Ngoma et al., 2013 ; Pattnaik et al., 2019 ; Susilowati et al., 2015 ; Verma et al., 2014 ; Verma et al., 2015a | Abdallah et al., 2019 ; Ali et al., 2018 ; Çakmakçı, 2019 ; Mitra et al., 2020 ; Ngoma et al., 2013 ; Verma et al., 2014 ;Verma et al., 2015a | Ali et al., 2018 ; Ambrosini et al., 2012 ; Belimov et al., 2005 ; Ghasemi et al., 2017 ; Verma et al., 2014 ; Verma et al., 2015a | Ali et al., 2018 ; Çakmakçı, 2019 ; Mitra et al., 2020 ; Park et al., 2005 ; Verma et al., 2014 ; Verma et al., 2015a ; Xu et al., 2018 | High resistance to metals (Ryan et al., 2009)  Resistant to radionucleide (Sarro et al., 2005)  Cd tolerant (Belimov et al., 2005)  High tolerance to Cd, Pb, Co, Zn, He or Ag (Ryan et al., 2009) | ACC deaminase production (Belimov et al., 2005; Pattnaik et al., 2019 ; Verma et al., 2014 ; Verma et al., 2015a)  Ammonia production (Abdallah et al., 2019 ; Ngoma et al., 2013; Verma et al., 2014)  Oxidation of elemental sulphur (Ryan et al., 2009)  Potassium solubilization (Verma et al., 2014, 2015a)  Zn solubilization (Ali et al., 2018)  Biocontrol of various phytopatogene including bacteria and fungus (Ryan et al., 2009; Verma et al., 2015a; Ali et al., 2018)  Nematode biocontrol mecanisms (Ryan et al., 2009; Topalovic et al., 2020)  Plant diseases reduction caused plant pathogens (Weinert et al., 2010)  Antibiotics production (Hashidoko et al., 1999; Ryan et al., 2009)  Hydrolytic enzymes production : proteases, lipases, nucleases, chitinases and elastases (Ryan et al., 2009)  Cellulose degradation (Susilowati et al., 2015)  Plant growth promotion for many plants, such as wheat, tomato, lettuce, sweet pepper, melon, celery and carrot in highly salinated soils of Uzbekistan (Ryan et al., 2009; Ali et al., 2018) |
| ***Lautropia*** |  |  |  |  |  | Hydrocarbon degradation (Khassali et al., 2020)  Nitrate and nitrite reduction (Gerner Smidt et al., 1994) |
| ***Leptolyngbya*** |  |  |  |  | Tsujimoto et al., 2016 | Complex EPS production able to form biocrust aggregating sand, chelating nutrients, and maintaining hydration (Chua et al., 2020; Mugnai et al., 2018)  Photosynthetic bacteria (Chua et al., 2020) |
| ***Nitrospira*** |  |  |  |  |  | Nitrite-oxidizing bacterium (Lebedeva et al., 2008)  Complete ammonia oxidation to nitrate by some species (Séneca et al., 2020) |
| ***Rathayibacter*** |  |  |  | Padda et al., 2018 |  |  |
| ***Saccharothrix*** |  |  |  |  | Resistant to Ni, Cd, Zn and Cu (Hamedi et al., 2015) | In vitro antagonistic activity (Weinert et al., 2010)  Antibiotics production (Adegboye and Babalola, 2016)  Protect plants against many phytopathogenes, bacteria and fungus (Muzammil et al., 2012) |
| ***Geminicoccus*** |  |  |  |  | Salt tolerance (Li et al., 2018) |  |
| ***Cellulomonas*** | Ali et al., 2018 ; Dell'Amico et al., 2005 ; Tsavkelova et al., 2005 ; Zhao et al., 2018 |  | Dell'Amico et al., 2005 | Khassali et al., 2020 ; Suleiman et al., 2019 | Resistant to Cd (Dell'Amico et al., 2005), Sb and Cu (Shi et al., 2013) | Corn root elongation (Ali et al., 2018)  Cellulose degradation lignin and hemicellulose degration (Amaya-Delgado et al., 2010 ; Khassali et al., 2020; Maki et al., 2009 ; Saratale et al., 2010 ; Tang et al., 2012)  Reduce Cr (Viamajala et al., 2007) and reduce/immobilize U (Sani et al., 2002) |
| ***Mesorhizobium*** | Ahemad and Khan, 2010; Ahemad and Khan, 2012 | Peix et al., 2001 | Ahemad and Khan, 2010; Ahemad and Khan, 2012 | Madhaiyan et al., 2015 ; Pattnaik et al., 2019 |  | ACC deaminase production (Del Carmen Orozco-Mosqueda et al., 2020 ; Nascimento et al., 2014;)  Promote growth of chickpea and barley plants (Peix et al., 2001)  Exopolysaccharide and ammonia production (Ahemad and Khan, 2010; Ahemad and Khan, 2012) |
| ***Bacillus*** | Ahmad et al., 2016 ; Bal et al., 2013 ; Das et al., 2014 ; Del Carmen Orozco-Mosqueda et al., 2020 ; Gupta and Pandey, 2019 ; Hernández-León et al., 2015 ; Kaushal and Wani, 2016; Rincon-Molina et al., 2020 ; Tahir et al., 2019; Upadhyay et al., 2011 ; Vardharajula et al., 2011 ; Verma et al., 2015b ; Yahaghi et al., 2019 ; Yu et al., 2014 | Das et al., 2014 ; Del Carmen Orozco-Mosqueda et al., 2020 ; Egamberdiyeva, 2007 ; Goswami et al., 2016 ; Gupta et al., 1994; Kaushal and Wani, 2016; Mitra et al., 2020 ; Nautiyal et al., 2013 ; Pattnaik et al., 2019 ; Ramakrishna et al., 2019 ; Rincon-Molina et al., 2020 ; Tahir et al., 2019 ; Upadhyay et al., 2011 ; Vardharajula et al., 2011 ; Verma et al., 2015b ; Zheng et al., 2018 | Das et al., 2014 ; Del Carmen Orozco-Mosqueda et al., 2020 ; Hernández-León et al., 2015 ; Pattnaik et al., 2019 ; Rincon-Molina et al., 2020 ; Vardharajula et al., 2011; Verma et al., 2015b ; Yahaghi et al., 2019 ; Yu et al., 2014 | Del Carmen Orozco-Mosqueda et al., 2020 ; Egamberdiyeva, 2007 ; Goswami et al., 2016 ; Park et al., 2005 ; Ramakrishna et al., 2019 ; Verma et al., 2015b ; Wu et al., 2006 | Resistant to Cd (Ahmad et al., 2016 ; Luo et al., 2011) and Hg (Durand et al., 2018)  Zn resistant (Verma et al., 2015b)  Very high As resistant (Das et al., 2014)  Specific to HgCl_2_-contaminated soils (Mera and Iwasaki 2007)  F-tolerant (Wang et al., 2018a)  Resistant to radionucleide (Sarro et al., 2005)  Multiple radionuclides removal (Sarró et al., 2005)  Cr reduction (Bruno et al., 2020) | Phytohormones production : cytokinins and gibberellins (Goswami et al., 2016; Kong and Glick, 2017; Numan et al., 2018 ; Vardharajula et al., 2011)  ACC deaminase production (Bal et al., 2013 ; Chinnaswamy et al., 2018 ; Das et al., 2014 ; Del Carmen Orozco-Mosqueda et al., 2020 ; Hernández-León et al., 2015 ; Rincon-Molina et al., 2020; Tahir et al., 2019)  Growth promotion of a wide number of plants (Alagawadi and Gaur, 1988; Gray and Smith, 2005; Goswami et al., 2016; Ortíz-Castro et al., 2008; Rincon-Molina et al., 2020; Turner and Blackman, 1991; Yan et al., 2003)  Nodulation increasement (Del Carmen Orozco-Mosqueda et al., 2020 ; Chinnaswamy et al., 2018)  Ammonia production (Bal et al., 2013 ; Vardharajula et al., 2011)  Potassium (Egamberdiyeva, 2007 ; Pattnaik et al., 2019 ; Verma et al., 2015b ; Wu et al., 2006) and Zn solubilization (Yahaghi et al., 2019)  Phosphorus and nitrogen uptake (Verma et al., 2015b)  Sulfur (Trifi et al., 2020) and thiosulfate oxidation (Siddikee et al., 2010)  Production of a wide variety of antibacterial and antifungal antibiotics (Goswami et al., 2016)  Many antagonistic activities agains plant pathogens (Compant et al., 2005 ; Kloepper et al., 2004; Mitra et al., 2020; Smith, et al., 1993; Weinert et al., 2010)  Induction of osmoprotectants production and interaction with other beneficial microorganisms (Del Carmen Orozco-Mosqueda et al., 2020; Nautiyal et al., 2013)  Nematode biocontrol mecanisms (Topalovic et al., 2020)  Cellulose degradation (Maki et al., 2009, Siddikee et al., 2010)  Hydrolytic enzymes production (Del Carmen Orozco-Mosqueda et al., 2020)  Protective effect on plant growth in abiotic stress (Tahir et al., 2019)  Reduce salt stress effect by different mecanisms such as root colonization, hydrogen cyanide, biofilm formation (Del Carmen Orozco-Mosqueda et al., 2020; Hernández-León et al., 2015) increasing activity of superoxide dismutase, catalase, phenols and proline, and K^+^ uptake (Damodaran et al., 2014; Kaushal and Wani, 2016) or regulating the ion transporter high-affinity K^+^ transporter that manage Na^+^ homeostasis (Kaushal and Wani, 2016; Zhang et al., 2008)  Reduce drought-stress effect by modifiing activity of antioxydant enzymes under drought stress (Kaushal and Wani, 2016; Timmusk et al., 2014; Vardharajula et al., 2011), by increasing chlorophyll content (Kaushal and Wani, 2016 ; Wang et al., 2012), increasing nutrients uptake, water content and decreasing stomatal conductance and electrolyte leakage (Kaushal and Wani, 2016; Ortiz et al., 2015) or modifying expression of genes ( Kaushal and Wani, 2016; Lim and Kim, 2013)  EPS production (Ahmad et al., 2016; Kaushal and Wani, 2016; Upadhyay et al., 2011 ; Vardharajula et al., 2011), proline production (Kaushal and Wani, 2016; Ortiz et al., 2015 ; Upadhyay et al., 2011; Vardharajula et al., 2011) and general amelioration of biochemical damages (Del Carmen Orozco-Mosqueda et al., 2020; Ferreira et al., 2018)  Cr reduction and As oxidation (Bachate et al., 2013)  Immobilisation of strontium from aqueous solution by sorption (Ghazvini et al., 2007) |
| ***Sphingobium*** | Ali et al., 2018 ; Pereira and Castro 2014 | Ali et al., 2018; Pereira and Castro 2014 | Ali et al., 2018 ; Pereira and Castro 2014 | Ali et al., 2018 | Cd tolerant (Zeng et al., 2020)  Hg resistant (Mahbub et al., 2016) | Corn root elongation Ali et al., 2018)  Zn solubilization (Ali et al., 2018)  Production of many antifungal antibiotics (Ali et al., 2018)  Potential candidate for remediating Hg by volatilization (Mahbub et al., 2016) |
| ***Rubrobacter*** |  |  |  |  | Radio Tolerant (Carreto et al., 1996; Chen et al., 2004; Li et al., 2014 ; Suzuki et al., 1988) | Typical extremophiles and lithotrophs (Li et al., 2015) |
| ***Caulobacter*** | Ali et al., 2018 ; Mehnaz et al., 2010 ; Pereira et al., 2016 | Ali et al., 2018 ; Mehnaz et al., 2010 | Ali et al., 2018 ; Pereira et al., 2016 | Ali et al., 2018 |  | ACC deaminse production (Mehnaz et al., 2010)  Corn root elongation (Ali et al., 2018)  Ammonia production (Pereira et al., 2016)  Production of multiple antifungal antibiotics (Ali et al., 2018)  Cellulase, pectinase, protease and lipase production (Pereira et al., 2016) |
| ***Phormidium*** | Boopathi et al., 2013 ; Hussain et al 2010 | Kishore et al., 2015 |  |  | Biosorption of Cu, Pb, and Cd (Kumar and Gaur, 2011) | Cytokinin production (Hussain et al 2010)  EPS production (Garcia-Meza et al., 2005) |
| ***Sulfurifustis*** |  |  |  |  |  | Sulfur oxidizer (Kojima et al., 2015 ; Liu et al., 2019) |
| ***Pseudonocardia*** | Borah and Thakur, 2020; Gangwar et al., 2014 ; Gong et al., 2018 ; Nimnoi et al., 2010; Suksaard et al., 2017 | Gangwar et al., 2014 ; Gong et al., 2018 ; Purushotham et al., 2018 ; Suksaard et al., 2017 | Borah and Thakur, 2020 ; Gangwar et al., 2014 ; Nimnoi et al., 2010 ; Suksaard et al., 2017 | Mahendra and Alvarez-Cohen, 2005 ; Sun et al., 2014 | Resistant to Ni, Co, Zn, Cd, Cu (Karelová et al., 2011; Remenár et al., 2014) | ACC deaminase production (Suksaard et al., 2017)  Rice growth promotion in normal consitions and under salinity stress (Suksaard et al., 2017)  Ammonia production (Borah and Thakur, 2020)  Produce antimicrobial compounds (Beemelmanns et al., 2016 ; Carr et al., 2012; Oh et al., 2009)  Antibacterial activities (Li et al., 2011)  Antibiotics production (Adegboye and Babalola, 2016)  Antibiotics production selectively inhibits the growth of the nest parasite *Escovopsis* but not the ants’ mutualistic fungus (Beemelmanns et al., 2016)  Cellulase, amylase and protease production (Gong et al., 2018)  Stimulate certain plant defense responses (Li et al., 2012)  Inhibition of mycelia growth in commercial tea cultivars (Borah and Thakur, 2020) |
| ***Nostoc*** | Hashtroudi et al., 2013 ; Kumar and Kaur, 2014; Osman et al., 2010 ; Singh, 2014 | Kishore et al., 2015; Kumar and Kaur, 2014; Moharana et al., 2018 |  | Meeks, 2005; Meeks, 2007; Pathak and Kumar, 2016 |  | Phytohormones production: cytokine and gibberelline (Osman et al., 2010, Singh, 2014)  Considerable growth-promoting effect on several vegetables and herbaceous plants (Hashtroudi et al., 2013)  Decrease the maximum soil temperature, retain soil moisture, increase soil organic matter, and reclaim degraded soil ecosystems (Obana et al., 2007)  Photosynthetic bacteria (Chua et al., 2020) |
| ***Legionella*** |  |  | Allard et al., 2006; Cianciotto, 2007 |  |  |  |
| ***Kushneria*** | Navarro-Torre et al., 2016 | Navarro-Torre et al., 2016; Navarro-Torre et al., 2018 ; Zhu et al., 2011 | Navarro-Torre et al., 2016 | Mapelli et al., 2013 ; Navarro-Torre et al., 2016 | Tolerate up to 25% NaCl (Navarro-Torre et al., 2018) | Ammonia production (Mapelli et al., 2013)  Nitrate reduction (Navarro-Torre et al., 2018)  Promote the growth of *Triticum aestivum* under chromium salt stress (Naseem et al 2016) |
| ***Solirubrobacter*** |  |  |  |  |  | Lignin compounds degradation (Kim et al., 2007)  Cellulose, hemicellulose and lignin degradation by secreting hydrolytic enzymes (Tang et al., 2012) |
| ***Kribbella*** | Borah and Thakur, 2020 | Borah and Thakur, 2020 |  | Borah and Thakur, 2020 |  | Ammonia production (Borah and Thakur, 2020)  Inhibition of mycelia growth in commercial tea cultivars (Borah and Thakur, 2020) |
| ***Pedomicrobium*** |  |  |  |  | Iron and manganese oxidizing (Braun et al., 2009) | Iron and manganese accumulation (Braun et al., 2009) |
| ***Methylobacterium*** | Kuffner et al., 2010 ; Ma et al., 2011 ; Madhaiyan et al., 2006 ; Rincon-Molina et al., 2020 ; Verma et al., 2013 ; Verma et al., 2014 , 2015a | Rincon-Molina et al., 2020 ; Verma et al., 2013 ; Verma et al., 2015a ; Verma et al., 2014 | Rincon-Molina et al., 2020 ; Verma et al., 2013 ; Verma et al., 2014 ; Verma et al., 2015a | Ali et al., 2018 ; Jourand et al., 2004 ; Madhaiyan et al., 2015 ; Verma et al., 2013 | Cd and Zn tolerant (Kuffner et al., 2010)  Ni, Cd biosorption (Ma et al., 2011)  Radio Resistant (Nogueira et al., 1998) | Phytohormones production : cytokinin (Madhaiyan et al., 2006) and gibberellins (Verma et al., 2015a)  ACC deaminase production (Kuffner et al., 2010 ; Ma et al., 2011 ; Madhaiyan et al., 2006 ; Rincon-Molina et al., 2020 ; Verma et al., 2015a)  Enhance growth of Phaseolus vulgaris and help N and P uptake (Rincon-Molina et al., 2020)  Corn root elongation (Ali et al., 2018)  Ammonia production (Verma et al., 2014, 2015a)  Antifungal antagonism against *P. ephenidermattum* on corn plant (Ali et al., 2018)  Cellulose production (Rincon-Molina et al., 2020)  EPS production (Rincon-Molina et al., 2020)  Ni and Cd toxicity reduction in tomato (Madhaiyan et al., 2007) |
| ***Georgenia*** |  |  | Cavalca et al., 2010 |  | As resistant (Cavalca et al., 2010) |  |
| ***Nitrosomonas*** |  | Moharana et al., 2018 |  |  |  |  |
| ***Myxococcus*** |  |  |  |  |  | Plant pathogen antagonist (Khassali et al., 2020)  Antibiotic production (Tyc et al., 2017 ; Xiao et al., 2011)  Insoluble organic compound degradation (Khassali et al., 2020)  Predatory soil bacterium (Morgan et al., 2010; Xiao et al., 2011) |
| ***Modestobacter*** |  |  |  | Adegboye and Babalola, 2012 | Resistant to desiccation, low nutrient, high radiation in arid area (Mohammadipanah and Wink, 2016)  Pb resistant (Gtari et al., 2012) and highly resistant to desiccation and to UV-radiation (Golinska et al., 2020) |  |
| ***Actinoplanes*** | El-Tarabily et al., 2009 ; El-Tarabily et al., 2010 ; Solans et al., 2011 |  |  |  |  | Phytohormones prodution : cytokinins and indole-3-pyruvic acid (IPYA) (El-Tarabily et al., 2009); gibberellin and zeatin (Solans et al., 2011)  ACC deaminase production (El-Tarabily et al., 2010)  Production of a wide variety of antibacterial and antifungal antibiotics (Enkh-Amgalan et al., 2012)  Reduction of root crown rots induced by *Pythium aphanidermatum* in cucumber (El-Tarabily et al., 2010) |
| ***Agromyces*** | Bal et al., 2013 ; Gong et al., 2018 | Kishore et al., 2015 |  |  | Ni, Cu, Zn, and Cd resistant (Chanda et al., 2017)  High Zn, Cd and Pb phytoextraction (Kuffner et al., 2008) | ACC deaminase production (Bal et al., 2013)  Zn and Cd extraction (Kuffner et al., 2008)  Biocontrol activity against nematode (Topalovic et al., 2020)  Cellulase, amylase, xylanase, and protease production (Gong et al., 2018)  Biochemical characters (Bal et al., 2013) |
| ***Aureimonas*** | Pirhadi et al., 2018 | Pirhadi et al., 2018 |  |  |  |  |
| ***Mycobacterium*** | Dell'Amico et al., 2008 ; Tsavkelova et al., 2005 ; Tsavkelova et al., 2007 | Ali et al., 2018 ; Adnan et al., 2017 ; Egamberdiyeva, 2007 ; Gong et al., 2018 | Palmer and Skaar, 2016; Tchakounté et al., 2018 | Ali et al., 2018 ; Egamberdiyeva, 2007 ; Sellstedt and Richau, 2013 | Cd phytoextraction (Dell'Amico et al., 2008  Zn, Co, and Ni resistant (Dell'Amico et al., 2005)  Intracellular accumulation of Cu (Mergeay, 1991)  Resistant to radionucleide (Sarro et al., 2005) | ACC deaminase production (Dell'Amico et al., 2005 ; 2008 ; Ma et al., 2011)  Plant root elongation promotion activity, shoot and root dry biomass (Dell'Amico et al., 2008; Ma et al., 2011)  Ammonia production (Gong et al., 2018)  Potassium uptake (Egamberdiyeva, 2007)  Cellulase production (Gong et al., 2018)  Aromatic compounds and hydrocarbons degradation (Child et al., 2007) and persistent organic pollutants (Tang et al., 2012)  Alkane degrading (Wallisch et al., 2014)  Salt tolerance (Tchakounté et al., 2018) |
| ***Erythrobacter*** |  | Tang et al., 2019 | Tang et al., 2019 |  |  | Enhance tomato shoot and root growth (Tang et al., 2019)  Antogonism activity against *Fusarium oxysporum* (Tang et al., 2019)  Volatile organic compound (Acetoin) production (Tang et al., 2019) |
| ***Corynebacterium*** | Ali et al., 2018 ; Bal et al., 2013 | Ali et al., 2018 ; Chinakwe et al., 2019 ; Pirhadi et al., 2018 | Verma et al., 2014 | Ali et al., 2018; Etesami, 2019 ; Giri and Pati, 2014; Sellstedt and Richau, 2013; Siddikee et al., 2010 |  | ACC deaminase production (Bal et al., 2013 ; Siddikee et al., 2010)  Corn root elongation (Ali et al., 2018)  Ammonia production (Bal et al., 2013 ; Chinakwe et al., 2019 ; Siddikee et al., 2010)  Thiosulfate oxidation (Siddikee et al., 2010)  Zn solubilization (Ali et al., 2018 ; Verma et al., 2014) |
| ***Glycomyces*** |  |  |  |  |  | Cellulose decomposition (Qian et al., 2020) |
| ***Ensifer*** | Cardinale et al., 2015 ; Oves et al., 2017 | Oves et al., 2017 | Cavalca et al., 2010 ; Oves et al., 2017 | Cardinale et al., 2015 ; Lamin et al., 2019 ; Xu et al., 2018 ; Zhou et al., 2013 | As resistant (Cavalca et al., 2010)  Cd, Cr, Cu, Zn and Ni tolerant (Oves et al., 2017)  Pb, Zn, Cu, Hg resistant (Lamin et al., 2019) | ACC deaminase production (Cavalca et al., 2010)  Ammonia production (Oves et al., 2017)  Calcium, phosphate and phytate mobilization under salt stress (Cardinale et al., 2015)  Ni and Pb bioaccumulation and biosorption (Oves et al., 2017) |
| ***Variovorax*** | Ali et al., 2018 ; Belimov et al., 2005; Kalam et al., 2017 ; Pereira and Castro 2014; Pereira et al., 2016 ; Solanki et al., 2017 | Ali et al., 2018 ; Pereira et al., 2016 ; Verma et al., 2013 ; Zheng et al., 2018 | Ali et al., 2018 ; Belimov et al., 2005 ; Kalam et al., 2017 ; Kuffner et al., 2010 ; Pereira and Castro 2014; Pereira et al., 2016 ; Verma et al., 2013 ; Zhang et al., 2012 | Ali et al., 2018 ; Solanki et al., 2017 | Hg-resistant (Durand et al., 2018)  Cd and Zn tolerant (Kuffner et al., 2010)  Cd tolerant (Belimov et al., 2005) | ACC deaminase production (Belimov et al., 2009 ; 2005 ; Jiang et al., 2012; Kaushal and Wani, 2016 ; Kuffner et al., 2010)  Corn root elongation (Ali et al., 2018)  Ammonia production (Pereira et al., 2016)  Potassium solubilization (Verma et al., 2013)  Zn solubilization (Ali et al., 2018 ; Kalam et al., 2017 ; Verma et al., 2013)  In vitro antagonistic activity (Weinert et al., 2010)  Nematode biocontrol mecanisms (Topalovic et al., 2020)  Antifungal antagonism against *V. dahliae* (Ali et al., 2018)  Biofilm Formation (Kalam et al., 2017)  Cellulase production (Pereira et al., 2016)  Increase uptake of N, P, K, Ca and Mg in *Pisum sativum* under drought stress (Kaushal and Wani, 2016 ; Jiang et al., 2012) |

**References**

Abd El-Azeem, S.A.M., Mehana, T.A. and Shabayek, A.A., 2007. Some plant growth promoting traits of rhizobacteria isolated from Suez Canal region, Egypt. In *African Crop Sci. Conf. Proceed* (Vol. 8, pp. 1517-1525).

Abdallah, R.A.B., Jabnoun-Khiareddine, H. and Daami-Remadi, M., 2019. In Secondary Metabolites of Plant Growth Promoting Rhizomicroorganisms (pp. 319-352). Springer, Singapore. <https://doi.org/10.1007/978-981-13-5862-3_16>

Adegboye, M.F. and Babalola, O.O., 2012. African Journal of Agricultural Research, 7(15), pp.2255-2261. <https://doi.org/10.5897/ajarx11.071>

Adegboye, M.F. and Babalola, O.O., 2016. Journal of Human Ecology, 56(1-2), pp.31-41. <https://doi.org/10.1080/09709274.2016.11907035>

Adnan, M., Shah, Z., Fahad, S., Arif, M., Alam, M., Khan, I.A., Mian, I.A., Basir, A., Ullah, H., Arshad, M. and Rahman, I.U., 2017. Scientific reports, 7(1), pp.1-13. <https://doi.org/10.1038/s41598-017-16537-5>

Adrangi, S., Faramarzi, M.A., Shahverdi, A.R. and Sepehrizadeh, Z., 2010. Carbohydrate research, 345(3), pp.402-407. <https://doi.org/10.1016/j.carres.2009.11.015>

Ahemad, M. and Khan, M.S., 2010. Pesticide biochemistry and physiology, 98(2), pp.183-190. <https://doi.org/10.1016/j.pestbp.2010.06.005>

Ahemad, M. and Khan, M.S., 2012. Journal of the Saudi Society of Agricultural Sciences, 11(1), pp.63-71. <https://doi.org/10.1016/j.jssas.2011.10.001>

Ahmad, I., Akhtar, M.J., Asghar, H.N., Ghafoor, U. and Shahid, M., 2016. Journal of plant growth regulation, 35(2), pp.303-315. <https://doi.org/10.1007/s00344-015-9534-5>

Akhtar, M.S. and Siddiqui, Z.A., 2009. Australasian Plant Pathology, 38(1), pp.44-50. <https://doi.org/10.1071/ap08075>

Alagawadi, A.R. and Gaur, A.C., 1988. Plant and Soil, 105(2), pp.241-246. <https://doi.org/10.1007/bf02376788>

Alami, Y., Achouak, W., Marol, C. and Heulin, T., 2000. Applied and environmental microbiology, 66(8), pp.3393-3398. <https://doi.org/10.1128/aem.66.8.3393-3398.2000>

Albuquerque, L., Simoes, C., Nobre, M.F., Pino, N.M., Battista, J.R., Silva, M.T., Rainey, F.A. and de Costa, M.S., 2005. FEMS microbiology letters, 247(2), pp.161-169. <https://doi.org/10.1016/j.femsle.2005.05.002>

Ali, S., Isaacson, J., Kroner, Y., Saldias, S., Kandasamy, S. and Lazarovits, G., 2018. Environmental Sustainability, 1(4), pp.341-355. <https://doi.org/10.1007/s42398-018-00030-4>

Ali, S.Z., Sandhya, V., Grover, M., Kishore, N., Rao, L.V. and Venkateswarlu, B., 2009. Biology and Fertility of Soils, 46(1), pp.45-55. <https://doi.org/10.1007/s00374-009-0404-9>

Aliyat, F.Z., Maldani, M., El Guilli, M., Nassiri, L. and Ibijbijen, J., 2020. Isolation and Characterization of Phosphate Solubilizing Bacteria from Phosphate Solid Sludge of the Moroccan Phosphate Mines. The Open Agriculture Journal, 14(1).

Allard, K.A., Viswanathan, V.K. and Cianciotto, N.P., 2006. Journal of bacteriology, 188(4), pp.1351-1363. <https://doi.org/10.1128/jb.188.4.1351-1363.2006>

Alvarez, A., Saez, J.M., Costa, J.S.D., Colin, V.L., Fuentes, M.S., Cuozzo, S.A., Benimeli, C.S., Polti, M.A. and Amoroso, M.J., 2017. Chemosphere, 166, pp.41-62. <https://doi.org/10.1016/j.chemosphere.2016.09.070>

Amaya-Delgado, L., Mejía-Castillo, T., Santiago-Hernández, A., Vega-Estrada, J., Amelia, F.G.S., Xoconostle-Cázares, B., Ruiz-Medrano, R., del Carmen Montes-Horcasitas, M. and Hidalgo-Lara, M.E., 2010. Bioresource technology, 101(14), pp.5539-5545. <https://doi.org/10.1016/j.biortech.2010.02.057>

Ambrosini, A., Beneduzi, A., Stefanski, T., Pinheiro, F.G., Vargas, L.K. and Passaglia, L.M., 2012. Plant and soil, 356(1-2), pp.245-264. <https://doi.org/10.1007/s11104-011-1079-1>

Anand, R., Grayston, S. and Chanway, C., 2013. Microbial Ecology, 66(2), pp.369-374. <https://doi.org/10.1007/s00248-013-0196-1>

Anwar, S., Ali, B. and Sajid, I., 2016. Frontiers in microbiology, 7, p.1334. <https://doi.org/10.3389/fmicb.2016.01334>

Ardley, J.K., Parker, M.A., De Meyer, S.E., Trengove, R.D., O’Hara, G.W., Reeve, W.G., Yates, R.J., Dilworth, M.J., Willems, A. and Howieson, J.G., 2012. International Journal of Systematic and Evolutionary Microbiology, 62(11), pp.2579-2588. <https://doi.org/10.1099/ijs.0.035097-0>

Asaf, S., Numan, M., Khan, A.L. and Al-Harrasi, A., 2020. Critical Reviews in Biotechnology, 40(2), pp.138-152. <https://doi.org/10.1080/07388551.2019.1709793>

Bachate, S.P., Nandre, V.S., Ghatpande, N.S. and Kodam, K.M., 2013. Chemosphere, 90(8), pp.2273-2278. <https://doi.org/10.1016/j.chemosphere.2012.10.081>

Bagade, A.V., Bachate, S.P., Dholakia, B.B., Giri, A.P. and Kodam, K.M., 2016. Journal of hazardous materials, 318, pp.742-750. <https://doi.org/10.1016/j.jhazmat.2016.07.062>

Bal, H.B., Das, S., Dangar, T.K. and Adhya, T.K., 2013. Journal of basic microbiology, 53(12), pp.972-984. <https://doi.org/10.1002/jobm.201200445>

Baldani, V.D., Baldani, J.I. and Döbereiner, J., 2000. Biology and Fertility of Soils, 30(5-6), pp.485-491. <https://doi.org/10.1007/s003740050027>

Barnawal, D., Bharti, N., Maji, D., Chanotiya, C.S. and Kalra, A., 2014. Journal of plant physiology, 171(11), pp.884-894. <https://doi.org/10.1016/j.jplph.2014.03.007>

Bastián, F., Cohen, A., Piccoli, P., Luna, V., Bottini, R. and Baraldi, R., 1998. Plant growth regulation, 24(1), pp.7-11. <https://doi.org/10.1023/a:1005964031159>

Becerra-Castro, C., Monterroso, C., Prieto-Fernández, A., Rodríguez-Lamas, L., Loureiro-Viñas, M., Acea, M.J. and Kidd, P.S., 2012. Journal of hazardous materials, 217, pp.350-359. <https://doi.org/10.1016/j.jhazmat.2012.03.039>

Beemelmanns, C., Guo, H., Rischer, M. and Poulsen, M., 2016. Beilstein journal of organic chemistry, 12(1), pp.314-327. <https://doi.org/10.3762/bjoc.12.34>

Belimov, A.A., Dodd, I.C., Hontzeas, N., Theobald, J.C., Safronova, V.I. and Davies, W.J., 2009. New Phytologist, 181(2), pp.413-423. <https://doi.org/10.1111/j.1469-8137.2008.02657.x>

Belimov, A.A., Hontzeas, N., Safronova, V.I., Demchinskaya, S.V., Piluzza, G., Bullitta, S. and Glick, B.R., 2005. Soil Biology and Biochemistry, 37(2), pp.241-250. <https://doi.org/10.1016/j.soilbio.2004.07.033>

Berg, G., Kurze, S., Buchner, A., Wellington, E.M. and Smalla, K. Canadian Journal of Microbiology, 46(12), pp.1128-1137. <https://doi.org/10.1139/w00-101>

Bhattacharyya, P.N. and Jha, D.K., 2012. World Journal of Microbiology and Biotechnology, 28(4), pp.1327-1350. <https://doi.org/10.1007/s11274-011-0979-9>

Bilal, S., Khan, A.L., Shahzad, R., Kim, Y.H., Imran, M., Khan, M.J., Al-Harrasi, A., Kim, T.H. and Lee, I.J., 2018. Ecotoxicology and environmental safety, 164, pp.648-658. <https://doi.org/10.1016/j.ecoenv.2018.08.043>

Biswas, R. and Sarkar, A., 2019. Preparative Biochemistry and Biotechnology, 49(1), pp.30-37. <https://doi.org/10.1080/10826068.2018.1476883>

Boopathi, T., Balamurugan, V., Gopinath, S. and Sundararaman, M., 2013. Journal of plant growth regulation, 32(4), pp.758-766. <https://doi.org/10.1007/s00344-013-9342-8>

Borah, A. and Thakur, D., 2020. Frontiers in Microbiology, 11, p.318. <https://doi.org/10.3389/fmicb.2020.00318>

Bosch, J., Lee, K.Y., Jordan, G., Kim, K.W. and Meckenstock, R.U., 2012. Environmental science & technology, 46(4), pp.2095-2101. <https://doi.org/10.1021/es2022329>

Braun, B., Richert, I. and Szewzyk, U., 2009. Journal of microbiological methods, 79(1), pp.37-43. <https://doi.org/10.1016/j.mimet.2009.07.014>

Brim, H., McFarlan, S.C., Fredrickson, J.K., Minton, K.W., Zhai, M., Wackett, L.P. and Daly, M.J., 2000. Nature biotechnology, 18(1), pp.85-90. <https://doi.org/10.1038/71986>

Brim, H., Venkateswaran, A., Kostandarithes, H.M., Fredrickson, J.K. and Daly, M.J., 2003. Applied and environmental microbiology, 69(8), pp.4575-4582. <https://doi.org/10.1128/AEM.69.8.4575-4582.2003>

Bruno, L.B., Karthik, C., Ma, Y., Kadirvelu, K., Freitas, H. and Rajkumar, M., 2020. Chemosphere, 244, p.125521. <https://doi.org/10.1016/j.chemosphere.2019.125521>

Bulgarelli, D., Schlaeppi, K., Spaepen, S., Van Themaat, E.V.L. and Schulze-Lefert, P., 2013. Annual review of plant biology, 64, pp.807-838. <https://doi.org/10.1146/annurev-arplant-050312-120106>

Çakmakçı, R., 2019. Alınteri Zirai Bilimler Dergisi, 34(2), pp.175-181. <https://doi.org/10.28955/alinterizbd.639020>

Cardinale, M., Ratering, S., Suarez, C., Montoya, A.M.Z., Geissler-Plaum, R. and Schnell, S., 2015. Microbiological research, 181, pp.22-32. <https://doi.org/10.1016/j.micres.2015.08.002>

Carr, G., Derbyshire, E.R., Caldera, E., Currie, C.R. and Clardy, J., 2012. Journal of natural products, 75(10), pp.1806-1809. <https://doi.org/10.1021/np300380t>

Carreto, L., Moore, E., Nobre, M.F., Wait, R., Riley, P.W., Sharp, R.J. and Da Costa, M.S., 1996. International Journal of Systematic and Evolutionary Microbiology, 46(2), pp.460-465. <https://doi.org/10.1099/00207713-46-2-460>

Castellano-Hinojosa, A., Correa‐Galeote, D., Palau, J. and Bedmar, E.J., 2016. Journal of basic microbiology, 56(1), pp.85-91. <https://doi.org/10.1002/jobm.201500247>

Castro, J.F., Nouioui, I., Sangal, V., Choi, S., Yang, S.J., Kim, B.Y., Trujillo, M.E., Riesco, R., Montero-Calasanz, M.D.C., Rahmani, T.P. and Bull, A.T., 2018. International journal of systematic and evolutionary microbiology, 68(9), pp.2712-2721. <https://doi.org/10.1099/ijsem.0.002828>

Cavalca, L., Zanchi, R., Corsini, A., Colombo, M., Romagnoli, C., Canzi, E. and Andreoni, V., 2010. Systematic and applied microbiology, 33(3), pp.154-164. <https://doi.org/10.1016/j.syapm.2010.02.004>

Chanda, D., Sharma, G.D., Jha, D.K. and Hijri, M., 2017. Recent advances in applied microbiology (pp. 165-193). Springer, Singapore. <https://doi.org/10.1007/978-981-10-5275-0_8>

Chen, M.Y., Wu, S.H., Lin, G.H., Lu, C.P., Lin, Y.T., Chang, W.C. and Tsay, S.S., 2004. International journal of systematic and evolutionary microbiology, 54(5), pp.1849-1855. <https://doi.org/10.1099/ijs.0.63109-0>

Child, R., Miller, C.D., Liang, Y., Sims, R.C. and Anderson, A.J., 2007. Journal of environmental quality, 36(5), pp.1260-1265. <https://doi.org/10.2134/jeq2007.0008>

Chinakwe, E.C., Ibekwe, V.I., Nwogwugwu, U.N., Ofoegbu, J., Mike-Anosike, E., Nwachukwu, I.N., Adeleye, S. and Chinakwe, P.O., 2019. Malaysian Journal of Sustainable Agriculture (MJSA), 3(1), pp.20-22. <https://doi.org/10.26480/mjsa.01.2019.20.22>

Chinnaswamy, A., Coba de la Peña, T., Stoll, A., de la Peña Rojo, D., Bravo, J., Rincón, A., Lucas, M.M. and Pueyo, J.J., 2018. Annals of Applied Biology, 172(3), pp.295-308. <https://doi.org/10.1111/aab.12420>

Chua, M., Erickson, T.E., Merritt, D.J., Chilton, A.M., Ooi, M.K. and Muñoz‐Rojas, M., 2020. Restoration Ecology, 28, pp.S168-S176. <https://doi.org/10.1111/rec.13040>

Cianciotto, N.P., 2007. Biometals, 20(3-4), pp.323-331. <https://doi.org/10.1007/s10534-006-9057-4>

Cobo-Díaz, J.F., Fernández-González, A.J., Villadas, P.J., Robles, A.B., Toro, N. and Fernández-López, M., 2015. Microbial ecology, 69(4), pp.895-904. <https://doi.org/10.1007/s00248-015-0586-7>

Cohen, A.C., Bottini, R., Pontin, M., Berli, F.J., Moreno, D., Boccanlandro, H., Travaglia, C.N. and Piccoli, P.N., 2015. Physiologia Plantarum, 153(1), pp.79-90. <https://doi.org/10.1111/ppl.12221>

Compant, S., Duffy, B., Nowak, J., Clément, C. and Barka, E.A., 2005. Applied and environmental microbiology, 71(9), pp.4951-4959. <https://doi.org/10.1128/aem.71.9.4951-4959.2005>

Coombs, J.T., Michelsen, P.P. and Franco, C.M., 2004. Biological Control, 29(3), pp.359-366. <https://doi.org/10.1016/j.biocontrol.2003.08.001>

Cotton, T.A., Pétriacq, P., Cameron, D.D., Al Meselmani, M., Schwarzenbacher, R., Rolfe, S.A. and Ton, J., 2019. The ISME journal, 13(7), pp.1647-1658. <https://doi.org/10.1038/s41396-019-0375-2>

Creus, C.M., Sueldo, R.J. and Barassi, C.A., 2004. Canadian Journal of Botany, 82(2), pp.273-281. <https://doi.org/10.1139/b03-119>

Damodaran, T., Rai, R.B., Jha, S.K., Kannan, R., Pandey, B.K., Sah, V., Mishra, V.K. and Sharma, D.K., 2014. Journal of plant interactions, 9(1), pp.577-584. <https://doi.org/10.1080/17429145.2013.873958>

Das, S., Jean, J.S., Kar, S., Chou, M.L. and Chen, C.Y., 2014. Journal of hazardous materials, 272, pp.112-120. <https://doi.org/10.1016/j.jhazmat.2014.03.012>

Dastager, S.G., Lee, J.C., Ju, Y.J., Park, D.J. and Kim, C.J., 2008. International journal of systematic and evolutionary microbiology, 58(5), pp.1060-1063. <https://doi.org/10.1099/ijs.0.65576-0>

Dastager, S.G., Pandey, A., Lee, J.C., Li, W.J. and Kim, C.J., 2009. Current microbiology, 59(1), pp.21-29. <https://doi.org/10.1007/s00284-009-9397-8>

Dastager, S.G., Raziuddin, Q.S., Deepa, C.K., Li, W.J. and Pandey, A., 2010. International journal of systematic and evolutionary microbiology, 60(12), pp.2867-2870. <https://doi.org/10.1099/ijs.0.019935-0>

Davidov, Y. and Jurkevitch, E., 2004. International journal of systematic and evolutionary microbiology, 54(5), pp.1439-1452. <https://doi.org/10.1099/ijs.0.02978-0>

de Lajudie, P., Laurent-Fulele, E., Willems, A., Torek, U., Coopman, R., Collins, M.D., Kersters, K., Dreyfus, B. and Gillis, M., 1998. International Journal of Systematic and Evolutionary Microbiology, 48(4), pp.1277-1290. <https://doi.org/10.1099/00207713-48-4-1277>

del Carmen Orozco-Mosqueda, M., Glick, B.R. and Santoyo, G., 2020. Microbiological Research, 235, p.126439. <https://doi.org/10.1016/j.micres.2020.126439>

Delavat, F., Lett, M.C. and Lièvremont, D., 2012. Biology Direct, 7(1), pp.1-14. <https://doi.org/10.1186/1745-6150-7-28>

Dell'Amico, E., Cavalca, L. and Andreoni, V., 2005. FEMS Microbiology Ecology, 52(2), pp.153-162. <https://doi.org/10.1016/j.femsec.2004.11.005>

Dell'Amico, E., Cavalca, L. and Andreoni, V., 2008. Soil Biology and Biochemistry, 40(1), pp.74-84. <https://doi.org/10.1016/j.soilbio.2007.06.024>

Dellweg, H., Kurz, J., Pflüger, W., Schedel, M., Vobis, G. and Wünsche, C., 1988. The Journal of antibiotics, 41(8), pp.1145-1147. <https://doi.org/10.7164/antibiotics.41.1145>

Dimitrijević, S., Pavlović, M., Maksimović, S., Ristić, M., Filipović, V., Antonović, D. and Dimitrijević‐Branković, S., 2018. Journal of the science of food and agriculture, 98(4), pp.1584-1590. <https://doi.org/10.1002/jsfa.8631>

Dimkpa, C.O., Merten, D., Svatoš, A., Büchel, G. and Kothe, E., 2009. Journal of Applied Microbiology, 107(5), pp.1687-1696. <https://doi.org/10.1111/j.1365-2672.2009.04355.x>

Du, Y., Yu, X. and Wang, G., 2012. International journal of systematic and evolutionary microbiology, 62(10), pp.2356-2362. <https://doi.org/10.1099/ijs.0.034306-0>

Durand, A., Maillard, F., Alvarez-Lopez, V., Guinchard, S., Bertheau, C., Valot, B., Blaudez, D. and Chalot, M., 2018. Science of The Total Environment, 622, pp.1165-1177. <https://doi.org/10.1016/j.scitotenv.2017.12.069>

Egamberdiyeva, D., 2007. Applied Soil Ecology, 36(2-3), pp.184-189. <https://doi.org/10.1016/j.apsoil.2007.02.005>

Elhady, A., Giné, A., Topalovic, O., Jacquiod, S., Sørensen, S.J., Sorribas, F.J. and Heuer, H., 2017. PloS one, 12(5), p.e0177145. https://doi.org/10.1371/journal.pone.0177145

El-Tarabily, K.A., Hardy, G.E.S.J. and Sivasithamparam, K., 2010. European Journal of Plant Pathology, 128(4), pp.527-539. <https://doi.org/10.1007/s10658-010-9689-7>

El‐Tarabily, K.A., Nassar, A.H., Hardy, G.S.J. and Sivasithamparam, K., 2009. Journal of Applied Microbiology, 106(1), pp.13-26. <https://doi.org/10.1111/j.1365-2672.2008.03926.x>

Enkh-Amgalan, J., Komaki, H., Daram, D., Ando, K. and Tsetseg, B., 2012. The Journal of antibiotics, 65(2), pp.103-108. <https://doi.org/10.1038/ja.2011.115>

Etesami, H., 2019. In Field Crops: Sustainable Management by PGPR (pp. 351-383). Springer, Cham. <https://doi.org/10.1007/978-3-030-30926-8_13>

Fan, P., Chen, D., He, Y., Zhou, Q., Tian, Y. and Gao, L., 2016. International journal of phytoremediation, 18(11), pp.1113-1121. <https://doi.org/10.1080/15226514.2016.1183583>

Faramarzi, M.A., Fazeli, M., Yazdi, M.T., Adrangi, S., Al-Ahmadi, K.J., Tasharrofi, N. and Mohseni, F.A., 2009. Biotechnology, 8(1), pp.93-99. <https://doi.org/10.3923/biotech.2009.93.99>

Farina, R., Beneduzi, A., Ambrosini, A., de Campos, S.B., Lisboa, B.B., Wendisch, V., Vargas, L.K. and Passaglia, L.M., 2012. Applied Soil Ecology, 55, pp.44-52. <https://doi.org/10.1016/j.apsoil.2011.12.011>

Feng, G.D., Yang, S.Z., Li, H.P. and Zhu, H.H., 2016. International Journal of Systematic and Evolutionary Microbiology, 66(1), pp.50-55. <https://doi.org/10.1099/ijsem.0.000670>

Ferreira, N.C., Mazzuchelli, R.D.C.L., Pacheco, A.C., Araujo, F.F.D., Antunes, J.E.L. and Araujo, A.S.F.D., 2018. Ciência Rural, 48(8). <https://doi.org/10.1590/0103-8478cr20170910>

Figueiredo, M.V.B., Martinez, C.R., Burity, H.A. and Chanway, C.P., 2008. World Journal of Microbiology and Biotechnology, 24(7), pp.1187-1193. <https://doi.org/10.1007/s11274-007-9591-4>

Finkmann, W., Altendorf, K., Stackebrandt, E. and Lipski, A., 2000. International journal of systematic and evolutionary microbiology, 50(1), pp.273-282. <https://doi.org/10.1099/00207713-50-1-273>

Foesel, B.U., Rohde, M. and Overmann, J., 2013. Systematic and applied microbiology, 36(2), pp.82-89. <https://doi.org/10.1016/j.syapm.2012.11.002>

Folman, L.B., Postma, J. and van Veen, J.A., 2003. Microbiological research, 158(2), pp.107-115. <https://doi.org/10.1078/0944-5013-00185>

Fukami, J., Cerezini, P. and Hungria, M., 2018. AMB Express, 8(1), p.73. <https://doi.org/10.1186/s13568-018-0608-1>

Gangwar, M., Khushboo, S.P. and Saini, P., 2014. J Biol Chem Sci, 1(1), pp.13-23.

García‐Meza, J.V., Barrangue, C. and Admiraal, W., 2005. Environmental Toxicology and Chemistry: An International Journal, 24(3), pp.573-581. <https://doi.org/10.1897/04-064r.1>

Gerner-Smidt, P., Keiser-Nielsen, H., Dorsch, M., Stackebrandt, E., Ursing, J., Blom, J., Christensen, A.C., Christensen, J.J., Frederiksen, W., Hoffmann, S. and Holten-Andersen, W., 1994. Microbiology, 140(7), pp.1787-1797. <https://doi.org/10.1099/13500872-140-7-1787>

Ghasemi, Z., Ghaderian, S.M., Rodríguez-Garrido, B., Prieto-Fernández, Á. and Kidd, P.S., 2018. Plant and Soil, 425(1-2), pp.265-285. <https://doi.org/10.1007/s11104-017-3553-x>

Ghazvini, P.T.M., Mashkani, S.G. and Ghafourian, H., 2007. Biosorption of Strontium from Aqueous Solution by New Strain.

Gholami, A., Shahsavani, S. and Nezarat, S., 2009. Int J Biol Life Sci, 1(1), pp.35-40. <https://doi.org/10.3923/pjbs.2009.26.32>

Giri, S. and Pati, B.R., 2004. Acta microbiologica et immunologica Hungarica, 51(1-2), pp.47-56. <https://doi.org/10.1556/AMicr.51.2004.1-2.3>

Golinska, P., del Carmen Montero-Calasanz, M., Świecimska, M., Yaramis, A., Igual, J.M., Bull, A.T. and Goodfellow, M., 2020. Atacama Desert soil. Systematic and Applied Microbiology, 43(1), p.126051. <https://doi.org/10.1016/j.syapm.2019.126051>

Gong, Y., Bai, J.L., Yang, H.T., Zhang, W.D., Xiong, Y.W., Ding, P. and Qin, S., 2018. Systematic and applied microbiology, 41(5), pp.516-527. <https://doi.org/10.1016/j.syapm.2018.06.003>

Goswami, D., Thakker, J.N. and Dhandhukia, P.C., 2016. Cogent Food & Agriculture, 2(1), p.1127500. <https://doi.org/10.1080/23311932.2015.1127500>

Goswami, M. and Suresh, D.E.K.A., 2020. Pedosphere, 30(1), pp.40-61. <https://doi.org/10.1016/S1002-0160(19)60839-8>

Govarthanan, M., Lee, G.W., Park, J.H., Kim, J.S., Lim, S.S., Seo, S.K., Cho, M., Myung, H., Kamala-Kannan, S. and Oh, B.T., 2014. Chemosphere, 109, pp.42-48. <https://doi.org/10.1016/j.chemosphere.2014.02.054>

Gray, E.J. and Smith, D.L., 2005. Soil biology and biochemistry, 37(3), pp.395-412. <https://doi.org/10.1016/j.soilbio.2004.08.030>

Grifoll, M., Selifonov, S.A., Gatlin, C.V. and Chapman, P.J., 1995. Applied and environmental microbiology, 61(10), pp.3711-3723. <https://doi.org/10.1128/AEM.61.10.3711-3723.1995>

Gtari, M., Essoussi, I., Maaoui, R., Sghaier, H., Boujmil, R., Gury, J., Pujic, P., Brusetti, L., Chouaia, B., Crotti, E. and Daffonchio, D., 2012. FEMS microbiology ecology, 80(3), pp.566-577. <https://doi.org/10.1111/j.1574-6941.2012.01320.x>

Guerrero-Zúñiga, A.L., López-López, E., Rodríguez-Tovar, A.V. and Rodríguez-Dorantes, A., 2020. Bioremediation of Industrial Waste for Environmental Safety (pp. 237-255). Springer, Singapore. <https://doi.org/10.1007/978-981-13-3426-9_10>

Guo, H., Nasir, M., Lv, J., Dai, Y. and Gao, J., 2017. Ecotoxicology and Environmental Safety, 144, pp.300-306. <https://doi.org/10.1016/j.ecoenv.2017.06.048>

Gupta, R., Singal, R., Shankar, A., Kuhad, R.C. and Saxena, R.K., 1994. The Journal of General and Applied Microbiology, 40(3), pp.255-260. <https://doi.org/10.2323/jgam.40.255>

Gupta, S., and Pandey, S., 2019. Frontiers in Microbiology, 10, p.1506. <https://doi.org/10.3389/fmicb.2019.01506>

Halo, B.A., Khan, A.L., Waqas, M., Al-Harrasi, A., Hussain, J., Ali, L., Adnan, M. and Lee, I.J., 2015. Journal of plant interactions, 10(1), pp.117-125. <https://doi.org/10.1080/17429145.2015.1033659>

Hamdali, H., Bouizgarne, B., Hafidi, M., Lebrihi, A., Virolle, M.J. and Ouhdouch, Y., 2008. Applied soil ecology, 38(1), pp.12-19. <https://doi.org/10.1016/j.apsoil.2007.08.007>

Hamdia, M.A.E.S., Shaddad, M.A.K. and Doaa, M.M., 2004. Plant Growth Regulation, 44(2), pp.165-174. <https://doi.org/10.1023/B:GROW.0000049414.03099.9b>

Hamedi, J., Dehhaghi, M. and Mohammdipanah, F., 2015. Isolation of Extremely Heavy Metal Resistant Strains of Rare Actinomycetes from High Metal Content Soils in Iran. International Journal of Environmental Research, 9(2).

Hamedi, J., Mohammadipanah, F. and Panahi, H.K.S., 2015. Halophiles (pp. 57-143). Springer, Cham. <https://doi.org/10.1007/978-3-319-14595-2_3>

Han, Q.Q., Lü, X.P., Bai, J.P., Qiao, Y., Paré, P.W., Wang, S.M., Zhang, J.L., Wu, Y.N., Pang, X.P., Xu, W.B. and Wang, Z.L., 2014. Frontiers in plant science, 5, p.525. <https://doi.org/10.3389/fpls.2014.00525>

Hashidoko, Y., Nakayama, T., Homma, Y. and Tahara, S., 1999. Tetrahedron letters, 40(15), pp.2957-2960. <https://doi.org/10.1016/S0040-4039(99)00336-6>

Hashtroudi, M.S., Ghassempour, A., Riahi, H., Shariatmadari, Z. and Khanjir, M., 2013. Journal of applied phycology, 25(2), pp.379-386. <https://doi.org/10.1007/s10811-012-9872-7>

Hassani, M.A., Durán, P. and Hacquard, S., 2018. Microbiome, 6(1), p.58. <https://doi.org/10.1186/s40168-018-0445-0>

Hastuti, R.D., Lestari, Y., Suwanto, A. and Saraswati, R., 2012. Journal of Biosciences, 19(4), p.155. <https://doi.org/10.4308/hjb.19.4.155>

Hayat, R., Ali, S., Amara, U., Khalid, R. and Ahmed, I., 2010. Annals of microbiology, 60(4), pp.579-598. <https://doi.org/10.1007/s13213-010-0117-1>

He, L.Y., Zhang, Y.F., Ma, H.Y., Chen, Z.J., Wang, Q.Y., Qian, M. and Sheng, X.F., 2010. Applied Soil Ecology, 44(1), pp.49-55. <https://doi.org/10.1016/j.apsoil.2009.09.004>

Hernández-León, R., Rojas-Solís, D., Contreras-Pérez, M., del Carmen Orozco-Mosqueda, M., Macías-Rodríguez, L.I., Reyes-de la Cruz, H., Valencia-Cantero, E. and Santoyo, G., 2015. Biological Control, 81, pp.83-92. <https://doi.org/10.1016/j.biocontrol.2014.11.011>

Herrera, H., Novotná, A., Ortiz, J., Soto, J. and Arriagada, C., 2020. Applied Soil Ecology, 149, p.103512. <https://doi.org/10.1016/j.apsoil.2020.103512>

Hu, W., Samac, D.A., Liu, X. and Chen, S., 2017. Applied Soil Ecology, 119, pp.396-406. <https://doi.org/10.1016/j.apsoil.2017.07.018>

Huang, L.N., Kuang, J.L. and Shu, W.S., 2016. Trends in microbiology, 24(7), pp.581-593. <https://doi.org/10.1016/j.tim.2016.03.004>

Hungria, M., 2011. A inoculação com estirpes selecionadas de *Azospirillum brasilense* e A. lipoferum melhora rendimentos de milho e trigo no Brasil. Embrapa Soja, Londrina, 331(1-2), pp.413-425.

Hussain, A., Krischke, M., Roitsch, T. and Hasnain, S., 2010. Current microbiology, 61(5), pp.361-369. <https://doi.org/10.1007/s00284-010-9620-7>

Islam, M.T., Hashidoko, Y., Deora, A., Ito, T. and Tahara, S., 2005. Applied and Environmental Microbiology, 71(7), pp.3786-3796. <https://doi.org/10.1128/AEM.71.7.3786-3796.2005>

Ivanova, J., Djingova, R., Korhammer, S. and Markert, B., 2001. Talanta, 54(4), pp.567-574. <https://doi.org/10.1016/S0039-9140(00)00640-8>

Iwata, K., Azlan, A., Yamakawa, H. and Omori, T., 2010. Journal of bioscience and bioengineering, 110(4), pp.415-418. <https://doi.org/10.1016/j.jbiosc.2010.05.006>

Jiang, F., Chen, L., Belimov, A.A., Shaposhnikov, A.I., Gong, F., Meng, X., Hartung, W., Jeschke, D.W., Davies, W.J. and Dodd, I.C., 2012. Journal of Experimental Botany, 63(18), pp.6421-6430. <https://doi.org/10.1093/jxb/ers301>

Jog, R., Nareshkumar, G. and Rajkumar, S., 2012. Journal of applied microbiology, 113(5), pp.1154-1164. <https://doi.org/10.1111/j.1365-2672.2012.05417.x>

Jourand, P., Giraud, E., Bena, G., Sy, A., Willems, A., Gillis, M., Dreyfus, B. and de Lajudie, P., 2004. International Journal of Systematic and Evolutionary Microbiology, 54(6), pp.2269-2273. <https://doi.org/10.1099/ijs.0.02902-0>

Kalam, S., Das, S.N., Basu, A. and Podile, A.R., 2017. Journal of basic microbiology, 57(5), pp.376-385. <https://doi.org/10.1002/jobm.201600588>

Kamala, K., Sivaperumal, P., Thilagaraj, R. and Natarajan, E., 2020. Bioremediation of Sr^2+^ ion radionuclide by using marine Streptomyces sp. CuOff24 extracellular polymeric substances. Journal of Chemical Technology & Biotechnology, 95(4), pp.893-903.

Kang, S.M., Khan, A.L., Hamayun, M., Hussain, J., Joo, G.J., You, Y.H., Kim, J.G. and Lee, I.J., 2012. Journal of microbiology, 50(6), pp.902-909. <https://doi.org/10.1007/s12275-012-2273-4>

Kang, S.M., Khan, A.L., Waqas, M., You, Y.H., Kim, J.H., Kim, J.G., Hamayun, M. and Lee, I.J., 2014. Journal of Plant Interactions, 9(1), pp.673-682. <https://doi.org/10.1080/17429145.2014.894587>

Kannojia, P., Sharma, P.K. and Sharma, K., 2019. Climate Change and Agricultural Ecosystems (pp. 43-64). Woodhead Publishing. <https://doi.org/10.1016/B978-0-12-816483-9.00003-7>

Karelová, E., Harichová, J., Stojnev, T., Pangallo, D. and Ferianc, P., 2011. Biologia, 66(1), pp.18-26. <https://doi.org/10.2478/s11756-010-0145-0>

Kasim, W.A., Gaafar, R.M., Abou-Ali, R.M., Omar, M.N. and Hewait, H.M., 2016. Annals of Agricultural Sciences, 61(2), pp.217-227. <https://doi.org/10.1016/j.aoas.2016.07.003>

Kaushal, M. and Wani, S.P., 2016. Agriculture, Ecosystems & Environment, 231, pp.68-78. <https://doi.org/10.1016/j.agee.2016.06.031>

Khan, A.L., Waqas, M., Kang, S.M., Al-Harrasi, A., Hussain, J., Al-Rawahi, A., Al-Khiziri, S., Ullah, I., Ali, L., Jung, H.Y. and Lee, I.J., 2014. Journal of Microbiology, 52(8), pp.689-695. <https://doi.org/10.1007/s12275-014-4002-7>

Khassali, H., Baumel, A., Mahé, F., Tournier, E., Tisseyre, P., Prin, Y., Ouahmane, L. and Sanguin, H., 2020. Ecological Indicators, 114, p.106341. <https://doi.org/10.1016/j.ecolind.2020.106341>

Kilic-Ekici, O. and Yuen, G.Y., 2004. Biological Control, 30(2), pp.446-455. <https://doi.org/10.1016/j.biocontrol.2004.01.014>

Kim, M.K. and Srinivasan, S., 2020. Archives of Microbiology, pp.1-7. <https://doi.org/10.1007/s00203-020-02033-4>

Kim, M.K., Na, J.R., Lee, T.H., Im, W.T., Soung, N.K. and Yang, D.C., 2007. International journal of systematic and evolutionary microbiology, 57(7), pp.1453-1455. <https://doi.org/10.1099/ijs.0.64715-0>

Kimes, N.E., López-Pérez, M., Flores-Félix, J.D., Ramírez-Bahena, M.H., Igual, J.M., Peix, A., Rodriguez-Valera, F. and Velázquez, E., 2015. Systematic and applied microbiology, 38(5), pp.293-299. <https://doi.org/10.1016/j.syapm.2015.05.003>

Kishore, N., Pindi, P.K. and Reddy, S.R., 2015. Plant biology and biotechnology (pp. 307-333). Springer, New Delhi. <https://doi.org/10.1007/978-81-322-2286-6_12>

Kloepper, J.W., Ryu, C.M. and Zhang, S., 2004. Phytopathology, 94(11), pp.1259-1266. <https://doi.org/10.1094/PHYTO.2004.94.11.1259>

Kojima, H., Shinohara, A. and Fukui, M., 2015. International journal of systematic and evolutionary microbiology, 65(10), pp.3709-3713. <https://doi.org/10.1099/ijsem.0.000479>

Kolton, M., Erlacher, A., Berg, G. and Cytryn, E., 2016. Microbial models: From environmental to industrial sustainability (pp. 189-207). Springer, Singapore. <https://doi.org/10.1007/978-981-10-2555-6_9>

Kong, Z. and Glick, B.R., 2017. Advances in microbial physiology (Vol. 71, pp. 97-132). Academic Press. <https://doi.org/10.1016/bs.ampbs.2017.04.001>

Kuffner, M., De Maria, S., Puschenreiter, M., Fallmann, K., Wieshammer, G., Gorfer, M., Strauss, J., Rivelli, A.R. and Sessitsch, A., 2010. Journal of applied microbiology, 108(4), pp.1471-1484. <https://doi.org/10.1111/j.1365-2672.2010.04670.x>

Kuffner, M., Puschenreiter, M., Wieshammer, G., Gorfer, M. and Sessitsch, A., 2008. Plant and Soil, 304(1-2), pp.35-44. <https://doi.org/10.1007/s11104-007-9517-9>

Kumar, A. and Kaur, R., 2014. Impact of cyanobacterial filtrate on seed germination behaviour of wheat. Int J Basic Appl Biol, 1(1), pp.11-15.

Kumar, D. and Gaur, J.P., 2011. Bioresource Technology, 102(2), pp.633-640. <https://doi.org/10.1016/j.biortech.2010.08.014>

Kumar, S., Suyal, D.C., Bhoriyal, M. and Goel, R., 2018. Journal of Plant Nutrition, 41(8), pp.1035-1046. <https://doi.org/10.1080/01904167.2018.1433211>

Kwon, K.K., Woo, J.H., Yang, S.H., Kang, J.H., Kang, S.G., Kim, S.J., Sato, T. and Kato, C., 2007. International journal of systematic and evolutionary microbiology, 57(10), pp.2207-2211. <https://doi.org/10.1099/ijs.0.64863-0>

Lamin, H., Missbah El Idrissi, M., Alami, S., Bouhnik, O., ElFaik, S., Abdelmoumen, H. and Bedmar, E.J., 2019. Frontiers in microbiology, 10, p.1456. <https://doi.org/10.3389/fmicb.2019.01456>

Lasudee, K., Tokuyama, S., Lumyong, S. and Pathom-Aree, W., 2017. International journal of systematic and evolutionary microbiology, 58(1), pp.242-250. <https://doi.org/10.1099/ijs.0.65379-0>

Lee, J.Y., Moon, S.S., Yun, B.S., Yoo, I.D. and Hwang, B.K., 2004. Journal of natural products, 67(12), pp.2076-2078. <https://doi.org/10.1021/np049786v>

Li, J., Zhao, G.Z., Varma, A., Qin, S., Xiong, Z., Huang, H.Y., Zhu, W.Y., Zhao, L.X., Xu, L.H., Zhang, S. and Li, W.J., 2012. PloS one, 7(12), p.e51410. <https://doi.org/10.1371/journal.pone.0051410>

Li, S., Tian, X., Niu, S., Zhang, W., Chen, Y., Zhang, H., Yang, X., Zhang, W., Li, W., Zhang, S. and Ju, J., 2011. Marine drugs, 9(8), pp.1428-1439. <https://doi.org/10.3390/md9081428>

Li, X., Bond, P.L., Van Nostrand, J.D., Zhou, J. and Huang, L., 2015. Scientific reports, 5, p.12978. <https://doi.org/10.1038/srep12978>

Li, X., Huang, L., Bond, P.L., Lu, Y. and Vink, S., 2014. Ecological engineering, 68, pp.233-240. <https://doi.org/10.1016/j.ecoleng.2014.03.044>

Lim, J.H. and Kim, S.D., 2013. The plant pathology journal, 29(2), p.201. <https://doi.org/10.5423/PPJ.SI.02.2013.0021>

Lin, S.Y., Hameed, A., Arun, A.B., Liu, Y.C., Hsu, Y.H., Lai, W.A., Rekha, P.D. and Young, C.C., 2013. International journal of systematic and evolutionary microbiology, 63(11), pp.4100-4107. <https://doi.org/10.1099/ijs.0.048231-0>

Liu, F., Huang, G., Fallowfield, H., Guan, H., Zhu, L. and Hu, H., 2013. Springer Science & Business Media. <https://doi.org/10.1007/978-3-642-38154-6>

Liu, J.L., Yao, J., Wang, F., Min, N., Gu, J.H., Li, Z.F., Sunahara, G., Duran, R., Solevic-Knudsen, T., Hudson-Edwards, K.A. and Alakangas, L., 2019. Environmental Pollution, 247, pp.98-107. <https://doi.org/10.1016/j.envpol.2018.12.045>

Liu, Q., Liu, H.C., Zhang, J.L., Zhou, Y.G. and Xin, Y.H., 2016. International Journal of Systematic and Evolutionary Microbiology, 66(1), pp.315-318. <https://doi.org/10.1099/ijsem.0.000717>

Liu, Y., Guo, J., Li, L., Asem, M.D., Zhang, Y., Mohamad, O.A., Salam, N. and Li, W., 2017. Journal of Arid Land, 9(3), pp.432-445. <https://doi.org/10.1007/s40333-017-0015-5>

Luo, G., Shi, Z. and Wang, G., 2012. International journal of systematic and evolutionary microbiology, 62(7), pp.1659-1665. <https://doi.org/10.1099/ijs.0.034405-0>

Luo, G., Shi, Z., Wang, H. and Wang, G., 2012. International journal of systematic and evolutionary microbiology, 62(6), pp.1271-1276. <https://doi.org/10.1099/ijs.0.033746-0>

Luo, S.L., Chen, L., Chen, J.L., Xiao, X., Xu, T.Y., Wan, Y., Rao, C., Liu, C.B., Liu, Y.T., Lai, C. and Zeng, G.M., 2011. Chemosphere, 85(7), pp.1130-1138. <https://doi.org/10.1016/j.chemosphere.2011.07.053>

Ma, Y., Prasad, M.N.V., Rajkumar, M. and Freitas, H., 2011. Biotechnology advances, 29(2), pp.248-258. <https://doi.org/10.1016/j.biotechadv.2010.12.001>

Mahbub, K.R., Krishnan, K., Megharaj, M. and Naidu, R., 2016. Chemosphere, 144, pp.330-337. <https://doi.org/10.1016/j.chemosphere.2015.08.061>

Madhaiyan, M., Alex, T.H.H., Te Ngoh, S., Prithiviraj, B. and Ji, L., 2015. Biotechnology for biofuels, 8(1), p.222. <https://doi.org/10.1186/s13068-015-0404-y>

Madhaiyan, M., Poonguzhali, S. and Sa, T., 2007. Chemosphere, 69(2), pp.220-228. <https://doi.org/10.1016/j.chemosphere.2007.04.017>

Madhaiyan, M., Poonguzhali, S., Ryu, J. and Sa, T., 2006. Planta, 224(2), pp.268-278. <https://doi.org/10.1007/s00425-005-0211-y>

Mahendra, S. and Alvarez-Cohen, L., 2005. International Journal of Systematic and Evolutionary Microbiology, 55(2), pp.593-598. <https://doi.org/10.1099/ijs.0.63085-0>

Maki, M., Leung, K.T. and Qin, W., 2009. International journal of biological sciences, 5(5), p.500. <https://doi.org/10.7150/ijbs.5.500>

Mapelli, F., Marasco, R., Rolli, E., Barbato, M., Cherif, H., Guesmi, A., Ouzari, I., Daffonchio, D. and Borin, S., 2013. BioMed research international, 2013. <https://doi.org/10.1155/2013/248078>

Marques, A.P., Pires, C., Moreira, H., Rangel, A.O. and Castro, P.M., 2010. Soil Biology and Biochemistry, 42(8), pp.1229-1235. <https://doi.org/10.1016/j.soilbio.2010.04.014>

Mazzola, M., Fujimoto, D.K., Thomashow, L.S. and Cook, R.J., 1995. Applied and Environmental Microbiology, 61(7), pp.2554-2559. <https://doi.org/10.1128/AEM.61.7.2554-2559.1995>

Mazzola, M., Funnell, D.L. and Raaijmakers, J.M., 2004. Microbial ecology, 48(3), pp.338-348. <https://doi.org/10.1007/s00248-003-1067-y>

McBride, M.J., Liu, W., Lu, X., Zhu, Y. and Zhang, W., 2014. The Prokaryotes, 4, pp.577-593. <https://doi.org/10.1007/978-3-642-38954-2_382>

McNeill, B.J., Pakostova, E., Bain, J.G., Gould, W.D., Amos, R.T., Wilson, G.W., Ptacek, C.J. and Blowes, D.W., 2020. Applied Geochemistry, 114, p.104531. <https://doi.org/10.1016/j.apgeochem.2020.104531>

Meeks, J.C., 2005. Molecular Basis of Symbiosis (pp. 165-196). Springer, Berlin, Heidelberg. <https://doi.org/10.1007/3-540-28221-1_9>

Meeks, J.C., 2007. Prokaryotic symbionts in plants (pp. 181-205). Springer, Berlin, Heidelberg. <https://doi.org/10.1007/7171_2007_101>

Mehnaz, S., 2011. Bacteria in Agrobiology: Crop Ecosystems (pp. 165-187). Springer, Berlin, Heidelberg. <https://doi.org/10.1007/978-3-642-18357-7_7>

Mera, N. and Iwasaki, K., 2007. Applied microbiology and biotechnology, 77(2), pp.437-445. <https://doi.org/10.1007/s00253-007-1152-0>

Mergeay, M., 1991. Trends in biotechnology, 9(1), pp.17-24. <https://doi.org/10.1016/0167-7799(91)90007-5>

Mihajlovski, K., Rajilić-Stojanović, M. and Dimitrijević-Branković, S., 2020. Renewable Energy, 152, pp.627-633. <https://doi.org/10.1016/j.renene.2020.01.101>

Minamiyama, H., Shimizu, M., Kunoh, H., Furumai, T., Igarashi, Y., Onaka, H. and Yoshida, R., 2003. Journal of general plant pathology, 69(1), pp.65-70. <https://doi.org/10.1007/s10327-002-0014-y>

Mirza, M.S., Ahmad, W., Latif, F., Haurat, J., Bally, R., Normand, P. and Malik, K.A., 2001. Plant and Soil, 237(1), pp.47-54. <https://doi.org/10.1023/A:1013388619231>

Mitra, D., Anđelković, S., Panneerselvam, P., Senapati, A., Vasić, T., Ganeshamurthy, A.N., Chauhan, M., Uniyal, N., Mahakur, B. and Radha, T.K., 2020. Communications in Soil Science and Plant Analysis, 51(5), pp.645-657. <https://doi.org/10.1080/00103624.2020.1729379>

Mohammadipanah, F. and Wink, J., 2016. Frontiers in Microbiology, 6, p.1541. <https://doi.org/10.3389/fmicb.2015.01541>

Moharana, P.C., Meena, M.D. and Biswas, D.R., 2018. Role of Rhizospheric Microbes in Soil (pp. 167-202). Springer, Singapore. <https://doi.org/10.1007/978-981-13-0044-8_6>

Monciardini, P., Cavaletti, L., Schumann, P., Rohde, M. and Donadio, S., 2003. International journal of systematic and evolutionary microbiology, 53(2), pp.569-576. <https://doi.org/10.1099/ijs.0.02400-0>

Moreira, F.D.S., Costa, P.B.D., Souza, R.D., Beneduzi, A., Lisboa, B.B., Vargas, L.K. and Passaglia, L.M., 2016. Genetics and molecular biology, 39(1), pp.111-121. <https://doi.org/10.1590/1678-4685-GMB-2015-0140>

Morgan, A.D., MacLean, R.C., Hillesland, K.L. and Velicer, G.J., 2010. Applied and environmental microbiology, 76(20), pp.6920-6927. <https://doi.org/10.1128/AEM.00414-10>

Msaddak, A., Durán, D., Rejili, M., Mars, M., Ruiz-Argüeso, T., Imperial, J., Palacios, J. and Rey, L., 2017. Applied and environmental microbiology, 83(6). <https://doi.org/10.1128/AEM.02820-16>

Mugnai, G., Rossi, F., Felde, V.J.M.N.L., Colesie, C., Büdel, B., Peth, S., Kaplan, A. and De Philippis, R., 2018. Soil Biology and Biochemistry, 127, pp.318-328. <https://doi.org/10.1016/j.soilbio.2018.08.007>

Muzammil, S., 2012. Saccharothrix algeriensis NRRL B-24137: biocontrol properties, colonization and induced systemic resistance towards *Botrytis cinerea* on grapevine and Arabidopsis thaliana (Doctoral dissertation).

Nascimento, F.X., Rossi, M.J., Soares, C.R., McConkey, B.J. and Glick, B.R., 2014. PLoS One, 9(6), p.e99168. <https://doi.org/10.1371/journal.pone.0099168>

Naseem, S., Yasin, M., Faisal, M. and Ahmed, A., 2016. Comparative Study of Plant Growth Promoting Bacteria in Minimizing Toxic Effects of Chromium on Growth and Metabolic Activities in Wheat (*Triticum aestivum*). Journal of the Chemical Society of Pakistan, 38(3).

Nautiyal, C.S., Srivastava, S., Chauhan, P.S., Seem, K., Mishra, A. and Sopory, S.K., 2013. Plant Physiology and Biochemistry, 66, pp.1-9. <https://doi.org/10.1016/j.plaphy.2013.01.020>

Navarro-Noya, Y.E., Jan-Roblero, J., del Carmen González-Chávez, M., Hernández-Gama, R. and Hernández-Rodríguez, C., 2010. Antonie Van Leeuwenhoek, 97(4), pp.335-349. <https://doi.org/10.1007/s10482-010-9413-9>

Navarro-Torre, S., Carro, L., Rodríguez-Llorente, I.D., Pajuelo, E., Caviedes, M.Á., Igual, J.M., Redondo-Gómez, S., Camacho, M., Klenk, H.P. and del Carmen Montero-Calasanz, M., 2018. International journal of systematic and evolutionary microbiology, 68(9), pp.2800-2806. <https://doi.org/10.1099/ijsem.0.002897>

Navarro-Torre, S., Mateos-Naranjo, E., Caviedes, M.A., Pajuelo, E. and Rodríguez-Llorente, I.D., 2016. Marine pollution bulletin, 110(1), pp.133-142. <https://doi.org/10.1016/j.marpolbul.2016.06.070>

Neiverth, A., Delai, S., Garcia, D.M., Saatkamp, K., de Souza, E.M., de Oliveira Pedrosa, F., Guimarães, V.F., dos Santos, M.F., Vendruscolo, E.C.G. and da Costa, A.C.T., 2014. European journal of soil biology, 64, pp.1-5. <https://doi.org/10.1016/j.ejsobi.2014.07.001>

Ngoma, L., Esau, B. and Babalola, O.O., 2013. African journal of Biotechnology, 12(26).

Nicolle, J.L.C., Simmons, S., Bathe, S. and Norris, P.R., 2009. Microbiology, 155(4), pp.1302-1309. <https://doi.org/10.1099/mic.0.023192-0>

Nimnoi, P. and Ruanpanun, P., 2020. Biological Control, p.104244. <https://doi.org/10.1016/j.biocontrol.2020.104244>

Nimnoi, P., Pongsilp, N. and Lumyong, S., 2010. World Journal of Microbiology and Biotechnology, 26(2), pp.193-203. <https://doi.org/10.1007/s11274-009-0159-3>

Niu, X., Song, L., Xiao, Y. and Ge, W., 2018. Frontiers in microbiology, 8, p.2580. <https://doi.org/10.3389/fmicb.2017.02580>

Nogueira, F., Botelho, M.L. and Tenreiro, R., 1998. Radiation Physics and Chemistry, 52(1-6), pp.15-19. <https://doi.org/10.1016/S0969-806X(98)00024-3>

Nor, M.N.M., Sabaratnam, V. and Tan, G.Y.A., 2017. International journal of systematic and evolutionary microbiology, 67(4), pp.851-855. <https://doi.org/10.1099/ijsem.0.001683>

Numan, M., Bashir, S., Khan, Y., Mumtaz, R., Shinwari, Z.K., Khan, A.L., Khan, A. and Ahmed, A.H., 2018. Microbiological research, 209, pp.21-32. <https://doi.org/10.1016/j.micres.2018.02.003>

Obana, S., Miyamoto, K., Morita, S., Ohmori, M. and Inubushi, K., 2007. Journal of applied phycology, 19(6), pp.641-646. <https://doi.org/10.1007/s10811-007-9193-4>

O'Donnell, S.T., Rittmann, B.E. and Kavazanjian Jr, E., 2019. Geomicrobiology Journal, 36(6), pp.543-558. <https://doi.org/10.1080/01490451.2019.1581858>

Ofek, M., Hadar, Y. and Minz, D., 2012. PloS one, 7(7), p.e40117. <https://doi.org/10.1371/journal.pone.0040117>

Oh, D.C., Poulsen, M., Currie, C.R. and Clardy, J., 2009. Nature Chemical Biology, 5(6), pp.391-393. <https://doi.org/10.1038/nchembio.159>

Olanrewaju, O.S. and Babalola, O.O., 2019. Applied microbiology and biotechnology, 103(3), pp.1179-1188. <https://doi.org/10.1007/s00253-018-09577-y>

Orhan, F. and Demirci, A., 2020. Geomicrobiology Journal, pp.1-7. <https://doi.org/10.1080/01490451.2020.1761911>

Ortiz, N., Armada, E., Duque, E., Roldán, A. and Azcón, R., 2015. Journal of plant physiology, 174, pp.87-96. <https://doi.org/10.1016/j.jplph.2014.08.019>

Ortíz-Castro, R., Valencia-Cantero, E. and López-Bucio, J., 2008. Plant signaling & behavior, 3(4), pp.263-265. <https://doi.org/10.4161/psb.3.4.5204>

Osman, M.E.H., El-Sheekh, M.M., El-Naggar, A.H. and Gheda, S.F., 2010. Biology and fertility of soils, 46(8), pp.861-875. <https://doi.org/10.1007/s00374-010-0491-7>

Oves, M., Khan, M.S. and Qari, H.A., 2017. Journal of the Taiwan Institute of Chemical Engineers, 80, pp.540-552. <https://doi.org/10.1016/j.jtice.2017.08.026>

Ozer, G., Ergene, A. and Icgen, B., 2013. Geomicrobiology Journal, 30(5), pp.381-390. <https://doi.org/10.1080/01490451.2012.694977>

Padda, K.P., Puri, A. and Chanway, C.P., 2018. Canadian Journal of Forest Research, 48(12), pp.1601-1606. <https://doi.org/10.1139/cjfr-2018-0347>

Palmer, L.D. and Skaar, E.P., 2016. Annual review of genetics, 50, pp.67-91. <https://doi.org/10.1146/annurev-genet-120215-035146>

Palumbo, J.D., Yuen, G.Y., Jochum, C.C., Tatum, K. and Kobayashi, D.Y., 2005. Phytopathology, 95(6), pp.701-707. <https://doi.org/10.1094/PHYTO-95-0701>

Pandey, A., Trivedi, P., Kumar, B. and Palni, L.M.S., 2006. Current microbiology, 53(2), pp.102-107. <https://doi.org/10.1007/s00284-006-4590-5>

Panneerselvam, P., Kumar, U., Sahu, S., Mohapatra, S.D., Dangar, T.K., Parameswaran, C., Jahan, A., Senapati, A. and Govindharaj, G.P.P., 2018. Journal of invertebrate pathology, 157, pp.74-79. <https://doi.org/10.1016/j.jip.2018.08.004>

Park, J.H., Kim, R., Aslam, Z., Jeon, C.O. and Chung, Y.R., 2008. Journal of Systematic and Evolutionary Microbiology, 58(2), pp.387-392. <https://doi.org/10.1099/ijs.0.65290-0>

Park, M., Kim, C., Yang, J., Lee, H., Shin, W., Kim, S. and Sa, T., 2005. Microbiological Research, 160(2), pp.127-133. <https://doi.org/10.1016/j.micres.2004.10.003>

Passari, A.K., Mishra, V.K., Saikia, R., Gupta, V.K. and Singh, B.P., 2015. Frontiers in microbiology, 6, p.273. <https://doi.org/10.3389/fmicb.2015.00273>

Pathak, D.V. and Kumar, M., 2016. Microbial inoculants in sustainable agricultural productivity (pp. 197-209). Springer, New Delhi. <https://doi.org/10.1007/978-81-322-2647-5_11>

Pattnaik, S., Mohapatra, B., Kumar, U., Pattnaik, M. and Samantaray, D., 2019. Biofertilizers for Sustainable Agriculture and Environment (pp. 435-463). Springer, Cham. <https://doi.org/10.1007/978-3-030-18933-4_20>

Peix, A., Rivas-Boyero, A.A., Mateos, P.F., Rodriguez-Barrueco, C., Martınez-Molina, E. and Velazquez, E., 2001. Soil Biology and Biochemistry, 33(1), pp.103-110. <https://doi.org/10.1016/S0038-0717(00)00120-6>

Pereira, S.I. and Castro, P.M., 2014. Ecological Engineering, 73, pp.526-535. <https://doi.org/10.1016/j.ecoleng.2014.09.060>

Pereira, S.I.A., Monteiro, C., Vega, A.L. and Castro, P.M., 2016. Ecological Engineering, 87, pp.91-97. <https://doi.org/10.1016/j.ecoleng.2015.11.033>

Pirhadi, M., Enayatizamir, N., Motamedi, H. and Sorkheh, K., 2018. Appl Ecol Environ, 16, pp.725-739. <https://doi.org/10.15666/aeer/1601_725739>

Postma, J., Nijhuis, E.H. and Someus, E., 2010. Applied soil ecology, 46(3), pp.464-469. <https://doi.org/10.1016/j.apsoil.2010.08.016>

Prasad, K.S., Subramanian, V. and Paul, J., 2009. Biometals, 22(5), p.711. <https://doi.org/10.1007/s10534-009-9215-6>

Prasad, S., Manasa, B.P., Buddhi, S., Pratibha, M.S., Begum, Z., Bandi, S., Tirunagari, P. and Shivaji, S., 2013. International journal of systematic and evolutionary microbiology, 63(5), pp.1627-1632. <https://doi.org/10.1099/ijs.0.044420-0>

Puri, A., Padda, K.P. and Chanway, C.P., 2016. Biology and fertility of soils, 52(1), pp.119-125. <https://doi.org/10.1007/s00374-015-1051-y>

Purushotham, N., Jones, E., Monk, J. and Ridgway, H., 2018. Microbial ecology, 76(3), pp.729-740. <https://doi.org/10.1007/s00248-018-1153-9>

Qian, L., Duan, L., Lin, J., Yang, Y., Song, J., Wang, X., Zhao, J. and Xiang, W., 2020. Journal of Systematic and Evolutionary Microbiology, 70(5), pp.3096-3104. <https://doi.org/10.1099/ijsem.0.004131>

Radwan, T.E.S.E.D., Mohamed, Z.K. and Reis, V., 2002. Production of indole-3-acetic acid by different strains of *Azospirillum* and *Herbaspirillum spp*. Symbiosis.

Raj, D.P.R.S., Linda, R. and Babyson, R.S., 2014. Molecular characterization of phosphate solubilizing bacteria (PSB) and plant growth promoting rhizobacteria (PGPR) from pristine soil. Int J Innov Sci Eng Technol, 1, pp.317-324.

Rajkumar, M. and Freitas, H., 2008. Chemosphere, 71(5), pp.834-842. <https://doi.org/10.1016/j.chemosphere.2007.11.038>

Ramakrishna, W., Yadav, R. and Li, K., 2019. Applied Soil Ecology, 138, pp.10-18. <https://doi.org/10.1016/j.apsoil.2019.02.019>

Rana, A., Saharan, B., Joshi, M., Prasanna, R., Kumar, K. and Nain, L., 2011. Annals of microbiology, 61(4), pp.893-900. <https://doi.org/10.1007/s13213-011-0211-z>

Rashid, S., Charles, T.C. and Glick, B.R., 2012. Applied soil ecology, 61, pp.217-224. <https://doi.org/10.1016/j.apsoil.2011.09.011>

Rastogi, G., Osman, S., Vaishampayan, P.A., Andersen, G.L., Stetler, L.D. and Sani, R.K., 2010. Microbial ecology, 59(1), pp.94-108. <https://doi.org/10.1007/s00248-009-9598-5>

Reddy, G.S., Nagy, M. and Garcia-Pichel, F., 2006. International journal of systematic and evolutionary microbiology, 56(1), pp.51-58. <https://doi.org/10.1099/ijs.0.63764-0>

Remenár, M., Harichová, J., Zámocký, M., Pangallo, D., Szemes, T., Budiš, J., Soltys, K. and Ferianc, P., 2017. Biologia, 72(9), pp.971-981. <https://doi.org/10.1515/biolog-2017-0117>

Remenár, M., Karelová, E., Harichová, J., Zámocký, M., Krčová, K. and Ferianc, P., 2014. Biologia, 69(11), pp.1453-1463. <https://doi.org/10.2478/s11756-014-0451-z>

Rijavec, T., Viršek, M.K. and Lapanje, A., 2019. Science of The Total Environment, 671, pp.1094-1100. <https://doi.org/10.1016/j.scitotenv.2019.03.423>

Rincón-Molina, C.I., Martínez-Romero, E., Ruiz-Valdiviezo, V.M., Velázquez, E., Ruiz-Lau, N., Rogel-Hernández, M.A., Villalobos-Maldonado, J.J. and Rincón-Rosales, R., 2020. Applied Soil Ecology, 146, p.103390. <https://doi.org/10.1016/j.apsoil.2019.103390>

Rivas, R., Velázquez, E., Willems, A., Vizcaíno, N., Subba-Rao, N.S., Mateos, P.F., Gillis, M., Dazzo, F.B. and Martínez-Molina, E., 2002. Applied and environmental microbiology, 68(11), pp.5217-5222. <https://doi.org/10.1128/AEM.68.11.5217-5222.2002>

Rivas, R., Willems, A., Subba-Rao, N.S., Mateos, P.F., Dazzo, F.B., Kroppenstedt, R.M., Martínez-Molina, E., Gillis, M. and Velázquez, E., 2003. Systematic and applied microbiology, 26(1), pp.47-53. <https://doi.org/10.1078/072320203322337308>

Rivera, D., Mora, V., Lopez, G., Rosas, S., Spaepen, S., Vanderleyden, J. and Cassan, F., 2018. Journal of Applied Microbiology, 125(6), pp.1774-1785. <https://doi.org/10.1111/jam.14080>

Robinson, B., Russell, C., Hedley, M. and Clothier, B., 2001. Agriculture, ecosystems & environment, 87(3), pp.315-321. <https://doi.org/10.1016/S0167-8809(01)00146-3>

Rodrigues, E.P., Rodrigues, L.S., de Oliveira, A.L.M., Baldani, V.L.D., dos Santos Teixeira, K.R., Urquiaga, S. and Reis, V.M., 2008. Plant and Soil, 302(1-2), pp.249-261. <https://doi.org/10.1007/s11104-007-9476-1>

Rungin, S., Indananda, C., Suttiviriya, P., Kruasuwan, W., Jaemsaeng, R. and Thamchaipenet, A., 2012. Antonie Van Leeuwenhoek, 102(3), pp.463-472. <https://doi.org/10.1007/s10482-012-9778-z>

Ryan, R.P., Monchy, S., Cardinale, M., Taghavi, S., Crossman, L., Avison, M.B., Berg, G., Van Der Lelie, D. and Dow, J.M., 2009. Nature Reviews Microbiology, 7(7), pp.514-525. <https://doi.org/10.1038/nrmicro2163>

Sahoo, B., Ningthoujam, R. and Chaudhuri, S., 2019. International Microbiology, 22(1), pp.155-167. <https://doi.org/10.1007/s10123-018-00037-1>

Sandhya, V.S.K.Z., Ali, S.Z., Grover, M., Reddy, G. and Venkateswarlu, B., 2010. Plant Growth Regulation, 62(1), pp.21-30. <https://doi.org/10.1007/s10725-010-9479-4>

Sandhya, V.Z.A.S., Grover, M., Reddy, G. and Venkateswarlu, B., 2009. Biology and fertility of soils, 46(1), pp.17-26. <https://doi.org/10.1007/s00374-009-0401-z>

Sang, M.K., Shrestha, A., Kim, D.Y., Park, K., Pak, C.H. and Kim, K.D., 2013. The plant pathology journal, 29(2), p.154. <https://doi.org/10.5423/PPJ.OA.07.2012.0104>

Sani, R., Peyton, B., Smith, W., Apel, W. and Petersen, J., 2002. Applied Microbiology and Biotechnology, 60(1-2), pp.192-199. <https://doi.org/10.1007/s00253-002-1069-6>

Santiago, C.D., Yagi, S., Ijima, M., Nashimoto, T., Sawada, M., Ikeda, S., Asano, K., Orikasa, Y. and Ohwada, T., 2017. Microbes and environments, p.ME16127. <https://doi.org/10.1264/jsme2.ME16127>

Santoyo, G., Orozco-Mosqueda, M.D.C. and Govindappa, M., 2012. Biocontrol Science and Technology, 22(8), pp.855-872. <https://doi.org/10.1080/09583157.2012.694413>

Sar, P., Kazy, S.K., Paul, D. and Sarkar, A., 2013. Thermophilic microbes in environmental and industrial biotechnology (pp. 171-201). Springer, Dordrecht. <https://doi.org/10.1007/978-94-007-5899-5_6>

Saratale, G.D., Saratale, R.G., Lo, Y.C. and Chang, J.S., 2010. Biotechnology progress, 26(2), pp.406-416. <https://doi.org/10.1002/btpr.342>

Saravanakumar, D. and Samiyappan, R., 2007. Journal of Applied Microbiology, 102(5), pp.1283-1292. <https://doi.org/10.1111/j.1365-2672.2006.03179.x>

Sarma, R.K. and Saikia, R., 2014. Plant and soil, 377(1-2), pp.111-126. <https://doi.org/10.1007/s11104-013-1981-9>

Sarró, M.I., García, A.M. and Moreno, D.A., 2005. Biofilm formation in spent nuclear fuel pools and bioremediation of radioactive water. International Microbiology, 8(3), pp.223-230.

Saxon, E.B., Jackson, R.W., Bhumbra, S., Smith, T. and Sockett, R.E., 2014. BMC microbiology, 14(1), p.163. <https://doi.org/10.1186/1471-2180-14-163>

Sbabou, L., Idir, Y., Bruneel, O., Le Quéré, A., Aurag, J., Béna, G. and Filali-Maltouf, A., 2016. <https://doi.org/10.15226/sojmid/4/3/00156>

Schouten, A., van den Berg, G., Edel-Hermann, V., Steinberg, C., Gautheron, N., Alabouvette, C., De Vos, C.H., Lemanceau, P. and Raaijmakers, J.M., 2004. Molecular Plant-Microbe Interactions, 17(11), pp.1201-1211. <https://doi.org/10.1094/MPMI.2004.17.11.1201>

Sellstedt, A. and Richau, K.H., 2013. FEMS Microbiology Letters, 342(2), pp.179-186. <https://doi.org/10.1111/1574-6968.12116>

Séneca, J., Pjevac, P., Canarini, A., Herbold, C.W., Zioutis, C., Dietrich, M., Simon, E., Prommer, J., Bahn, M., Pötsch, E.M. and Wagner, M., 2020. The ISME Journal, pp.1-16. <https://doi.org/10.1038/s41396-020-00735-7>

Shao, J., Peng, L., Luo, S., Yu, G., Gu, J.D., Lin, S. and Li, R., 2013. Journal of applied phycology, 25(5), pp.1567-1573. <https://doi.org/10.1007/s10811-012-9969-z>

Sheng, X.F. and Xia, J.J., 2006. Chemosphere, 64(6), pp.1036-1042. <https://doi.org/10.1016/j.chemosphere.2006.01.051>

Sheng, X.F., Xia, J.J., Jiang, C.Y., He, L.Y. and Qian, M., 2008. Environmental pollution, 156(3), pp.1164-1170. <https://doi.org/10.1016/j.envpol.2008.04.007>

Shi, L., Du, N., Shu, S., Sun, J., Li, S. and Guo, S., 2017. Scientific reports, 7, p.41234. <https://doi.org/10.1038/srep41234>

Shi, Z., Cao, Z., Qin, D., Zhu, W., Wang, Q., Li, M. and Wang, G., 2013. PLoS One, 8(10), p.e78533. <https://doi.org/10.1371/journal.pone.0078533>

Siddikee, M.A., Chauhan, P.S., Anandham, R., Han, G.H. and Sa, T., 2010. J Microbiol Biotechnol, 20(11), pp.1577-1584. <https://doi.org/10.4014/jmb.1007.07011>

Silver, S. and Phung, L.T., 1996. Annual review of microbiology, 50(1), pp.753-789. <https://doi.org/10.1146/annurev.micro.50.1.753>

Singh, A.V., Chandra, R. and Goel, R., 2013. Archives of Agronomy and Soil Science, 59(5), pp.641-651. <https://doi.org/10.1080/03650340.2012.664767>

Singh, H., Du, J., Won, K., Yang, J.E., Yin, C., Kook, M. and Yi, T.H., 2015. International Journal of Systematic and Evolutionary Microbiology, 65(10), pp.3690-3696. <https://doi.org/10.1099/ijsem.0.000477>

Singh, I., 2018. Plant Growth Promoting Rhizobacteria (PGPR) and their various mechanisms for plant growth enhancement in stressful conditions: a review. European Journal of Biological Research, 8(4), pp.191-213.

Singh, N., Marwa, N., Mishra, J., Verma, P.C., Rathaur, S. and Singh, N., 2016. Ecotoxicology and Environmental Safety, 125, pp.25-34. <https://doi.org/10.1016/j.ecoenv.2015.11.020>

Singh, S., 2014. Journal of applied microbiology, 117(5), pp.1221-1244. <https://doi.org/10.1111/jam.12612>

Sly, L.I. and Stackebrandt, E., 1999. International Journal of Systematic and Evolutionary Microbiology, 49(2), pp.541-544. <https://doi.org/10.1099/00207713-49-2-541>

Smith, K.P., Havey, M.J. and Handelsman, J., 1993. Plant Disease, 77(2), pp.139-142. <https://doi.org/10.1094/PD-77-0139>

Solanki, M.K., Wang, Z., Wang, F.Y., Li, C.N., Lan, T.J., Singh, R.K., Singh, P., Yang, L.T. and Li, Y.R., 2017. Sugar Tech, 19(2), pp.136-147. <https://doi.org/10.1007/s12355-016-0445-y>

Solans, M., Vobis, G., Cassán, F., Luna, V. and Wall, L.G., 2011. World Journal of Microbiology and Biotechnology, 27(9), pp.2195-2202. <https://doi.org/10.1007/s11274-011-0685-7>

Soltani, A.A., Khavazi, K., Asadi-Rahmani, H., Omidvari, M., Dahaji, P.A. and Mirhoseyni, H., 2010. Journal of Agricultural Science, 2(4), p.106. <https://doi.org/10.5539/jas.v2n4p106>

Sood, S., Awal, R.P., Wink, J., Mohr, K.I., Rohde, M., Stadler, M., Kämpfer, P., Glaeser, S.P., Schumann, P., Garcia, R. and Müller, R., 2015. International journal of systematic and evolutionary microbiology, 65(3), pp.745-753. <https://doi.org/10.1099/ijs.0.061176-0>

Suksaard, P., Pathom-aree, W. and Duangmal, K., 2017. Diversity and plant growth promoting activities of actinomycetes from mangroves. Chiang Mai J Sci, 44(4), pp.1210-1223

Suleiman, M.K., Quoreshi, A.M., Bhat, N.R., Manuvel, A.J. and Sivadasan, M.T., 2019. PloS one, 14(12), p.e0220679. <https://doi.org/10.1371/journal.pone.0220679>

Sun, J., Zhang, Q., Zhou, J. and Wei, Q., 2014. Applied Soil Ecology, 78, pp.28-36. <https://doi.org/10.1016/j.apsoil.2014.02.004>

Sun, L.N., Zhang, Y.F., He, L.Y., Chen, Z.J., Wang, Q.Y., Qian, M. and Sheng, X.F., 2010. Bioresource Technology, 101(2), pp.501-509. <https://doi.org/10.1016/j.biortech.2009.08.011>

Suresh, K., Prabagaran, S.R., Sengupta, S. and Shivaji, S., 2004. International journal of systematic and evolutionary microbiology, 54(4), pp.1369-1375. <https://doi.org/10.1099/ijs.0.03047-0>

Susilowati, D.N., Sudiana, I., Mubarik, N.R., Agatis, J. and Campus, D., 2015. <https://doi.org/10.21082/ijas.v16n1.2015.39-50>

Suzuki, K.I., Collins, M.D., Iijima, E. and Komagata, K., 1988. FEMS microbiology letters, 52(1-2), pp.33-39. <https://doi.org/10.1111/j.1574-6968.1988.tb02568.x>

Tahir, M., Ahmad, I., Shahid, M., Shah, G.M., Farooq, A.B.U., Akram, M., Tabassum, S.A., Naeem, M.A., Khalid, U., Ahmad, S. and Zakir, A., 2019. Ecotoxicology and environmental safety, 178, pp.33-42. <https://doi.org/10.1016/j.ecoenv.2019.04.027>

Tan, Y., Wang, Y., Wang, D., Wang, G. and Zheng, S., 2016. International journal of systematic and evolutionary microbiology, 66(12), pp.4930-4935. <https://doi.org/10.1099/ijsem.0.001447>

Tang, Y.Q., Ji, P., Lai, G.L., Chi, C.Q., Liu, Z.S. and Wu, X.L., 2012. International Journal of Coal Geology, 90, pp.21-33. <https://doi.org/10.1016/j.coal.2011.09.009>

Tang, T., Sun, X., Dong, Y. and Liu, Q., 2019. Biotech, 9(11), p.430. <https://doi.org/10.1007/s13205-019-1958-3>

Tangaromsuk, J., Pokethitiyook, P., Kruatrachue, M. and Upatham, E.S., 2002. Bioresource Technology, 85(1), pp.103-105. <https://doi.org/10.1016/S0960-8524(02)00066-4>

Tapase, S.R. and Kodam, K.M., 2018. Chemosphere, 195, pp.1-10. <https://doi.org/10.1016/j.chemosphere.2017.12.022>

Tchakounté, G.V.T., Berger, B., Patz, S., Fankem, H. and Ruppel, S., 2018. Microbiological research, 214, pp.47-59. <https://doi.org/10.1016/j.micres.2018.05.008>

Tewari, S. and Arora, N.K., 2014. Current microbiology, 69(4), pp.484-494. <https://doi.org/10.1007/s00284-014-0612-x>

Thatoi, H., Das, S., Mishra, J., Rath, B.P. and Das, N., 2014. Journal of Environmental Management, 146, pp.383-399. <https://doi.org/10.1016/j.jenvman.2014.07.014>

Timmusk, S. and Wagner, E.G.H., 1999. Molecular plant-microbe interactions, 12(11), pp.951-959. <https://doi.org/10.1094/MPMI.1999.12.11.951>

Timmusk, S., Abd El-Daim, I.A., Copolovici, L., Tanilas, T., Kännaste, A., Behers, L., Nevo, E., Seisenbaeva, G., Stenström, E. and Niinemets, Ü., 2014. PloS one, 9(5), p.e96086. <https://doi.org/10.1371/journal.pone.0096086>

Topalović, O., Hussain, M. and Heuer, H., 2020. Frontiers in Microbiology, 11, p.313. <https://doi.org/10.3389/fmicb.2020.00313>

Trifi, H., Najjari, A., Achouak, W., Barakat, M., Ghedira, K., Mrad, F., Saidi, M. and Sghaier, H., 2020. Genomics, 112(1), pp.981-989. <https://doi.org/10.1016/j.ygeno.2019.06.014>

Tsavkelova, E.A., Cherdyntseva, T.A. and Netrusov, A.I., 2005. Microbiology, 74(1), pp.46-53. <https://doi.org/10.1007/s11021-005-0027-6>

Tsavkelova, E.A., Cherdyntseva, T.A., Klimova, S.Y., Shestakov, A.I., Botina, S.G. and Netrusov, A.I., 2007. Archives of Microbiology, 188(6), pp.655-664. <https://doi.org/10.1007/s00203-007-0286-x>

Turner, J.T. and Backman, P.A., 1991. Plant disease, 75(4), pp.347-353. <https://doi.org/10.1094/PD-75-0347>

Tyc, O., Song, C., Dickschat, J.S., Vos, M. and Garbeva, P., 2017. Trends in microbiology, 25(4), pp.280-292. <https://doi.org/10.1016/j.tim.2016.12.002>

Upadhyay, S.K., Singh, J.S. and Singh, D.P., 2011. Pedosphere, 21(2), pp.214-222. <https://doi.org/10.1016/S1002-0160(11)60120-3>

Vardharajula, S., Zulfikar Ali, S., Grover, M., Reddy, G. and Bandi, V., 2011. Journal of Plant Interactions, 6(1), pp.1-14. <https://doi.org/10.1080/17429145.2010.535178>

Verma, P., Yadav, A.N., Kazy, S.K., Saxena, A.K. and Suman, A., 2013. Elucidating the diversity and plant growth promoting attributes of wheat (*Triticum aestivum*) associated acidotolerant bacteria from southern hills zone of India. Natl J Life Sci, 10(2), pp.219-226.

Verma, P., Yadav, A.N., Kazy, S.K., Saxena, A.K. and Suman, A., 2014. Evaluating the diversity and phylogeny of plant growth promoting bacteria associated with wheat (*Triticum aestivum*) growing in central zone of India. Int J Curr Microbiol Appl Sci, 3(5), pp.432-447.

Verma, P., Yadav, A.N., Khannam, K.S., Panjiar, N., Kumar, S., Saxena, A.K. and Suman, A., 2015a. Annals of microbiology, 65(4), pp.1885-1899. <https://doi.org/10.1007/s13213-014-1027-4>

Verma, P., Yadav, A.N., Shukla, L., Saxena, A.K. and Suman, A., 2015b. Alleviation of cold stress in wheat seedlings by *Bacillus amyloliquefaciens*. IARI-HHS2-30, an endophytic psychrotolerant K-solubilizing bacterium from NW Indian Himalayas. Natl J Life Sci 12: 105-110.

Veyisoglu, A., Tatar, D., Saygin, H., Inan, K., Cetin, D., Guven, K., Tuncer, M. and Sahin, N., 2016. Antonie Van Leeuwenhoek, 109(2), pp.287-296. <https://doi.org/10.1007/s10482-015-0631-z>

Viamajala, S., Smith, W.A., Sani, R.K., Apel, W.A., Petersen, J.N., Neal, A.L., Roberto, F.F., Newby, D.T. and Peyton, B.M., 2007. Bioresource Technology, 98(3), pp.612-622. <https://doi.org/10.1016/j.biortech.2006.02.023>

Vives-Peris, V., Gómez-Cadenas, A. and Pérez-Clemente, R.M., 2018. Plant cell reports, 37(11), pp.1557-1569. <https://doi.org/10.1007/s00299-018-2328-z>

Wahyudi, A.T., Priyanto, J.A., Afrista, R., Kurniati, D., Astuti, R.I. and Akhdiya, A., 2019. Online J. Biol. Sci, 19, pp.1-8. <https://doi.org/10.3844/ojbsci.2019.1.8>

Wallisch, S. Gril, T., Dong, X., Welzl, G., Bruns, C., Heath, E., Engel, M., Suhadolc, M., Schloter, M., 2014. Frontiers in Microbiology, 5 <https://doi.org/10.3389/fmicb.2014.00096>

Wang, C.J., Yang, W., Wang, C., Gu, C., Niu, D.D., Liu, H.X., Wang, Y.P. and Guo, J.H., 2012. PLoS One, 7(12), p.e52565. <https://doi.org/10.1371/journal.pone.0052565>

Wang, K., Jia, R., Li, L., Jiang, R. and Qu, D., 2020. Journal of Soils and Sediments, 20(3), pp.1621-1631. <https://doi.org/10.1007/s11368-019-02529-7>

Wang, M., Tang, Y., Anderson, C.W., Jeyakumar, P. and Yang, J., 2018a. Environmental Science and Pollution Research, 25(16), pp.15336-15348. <https://doi.org/10.1007/s11356-018-1408-5>

Watanabe, T., Miura, A., Shinohara, A., Kojima, H. and Fukui, M., 2016. International Journal of Systematic and Evolutionary Microbiology, 66(5), pp.1986-1989. <https://doi.org/10.1099/ijsem.0.000972>

Weinert, N., Meincke, R., Gottwald, C., Radl, V., Dong, X., Schloter, M., Berg, G. and Smalla, K., 2010. Plant and Soil, 326(1-2), pp.437-452. <https://doi.org/10.1007/s11104-009-0024-z>

Weon, H.Y., Kim, B.Y., Hong, S.B., Joa, J.H., Nam, S.S., Lee, K.H. and Kwon, S.W., 2007. International journal of systematic and evolutionary microbiology, 57(7), pp.1539-1542. <https://doi.org/10.1099/ijs.0.64676-0>

Wu Sc, C.K., Luo, Y. and Wong, M., 2006. Environ. Pollut, 140(1), pp.124-135. <https://doi.org/10.1016/j.envpol.2005.06.023>

Xiao, X., Fan, M., Wang, E., Chen, W. and Wei, G., 2017. Applied microbiology and biotechnology, 101(23-24), pp.8485-8497. <https://doi.org/10.1007/s00253-017-8550-8>

Xiao, Y., Wei, X., Ebright, R. and Wall, D., 2011. Journal of bacteriology, 193(18), pp.4626-4633. <https://doi.org/10.1128/JB.05052-11>

Xie, X., Fu, J., Wang, H. and Liu, J., 2010. African Journal of Biotechnology, 9(26), pp.4056-4066.

Xu, J., Kloepper, J.W., Huang, P., McInroy, J.A. and Hu, C.H., 2018. Journal of basic microbiology, 58(5), pp.459-471. <https://doi.org/10.1002/jobm.201700535>

Xu, Y., Sun, G.D., Jin, J.H., Liu, Y., Luo, M., Zhong, Z.P. and Liu, Z.P., 2014. Journal of Hazardous Materials, 264, pp.430-438. <https://doi.org/10.1016/j.jhazmat.2013.10.071>

Yahaghi, Z., Shirvani, M., Nourbakhsh, F. and Pueyo, J.J., 2019. African Journal of Botany, 124, pp.573-582. <https://doi.org/10.1016/j.sajb.2019.01.006>

Yamanaka, T., 1996. Plant and cell physiology, 37(5), pp.569-574. <https://doi.org/10.1093/oxfordjournals.pcp.a028982>

Yan, Z., Reddy, M.S. and Kloepper, J.W., 2003. Canadian journal of microbiology, 49(6), pp.383-389. <https://doi.org/10.1139/w03-051>

Youseif, S.H., 2018. Annals of Agricultural Sciences, 63(1), pp.25-35. <https://doi.org/10.1016/j.aoas.2018.04.002>

Yu, X., Li, Y., Zhang, C., Liu, H., Liu, J., Zheng, W., Kang, X., Leng, X., Zhao, K., Gu, Y. and Zhang, X., 2014. PLoS One, 9(9), p.e106618. <https://doi.org/10.1371/journal.pone.0106618>

Zahir, Z.A., Ghani, U., Naveed, M., Nadeem, S.M. and Asghar, H.N., 2009. Archives of microbiology, 191(5), pp.415-424. <https://doi.org/10.1007/s00203-009-0466-y>

Zeng, X., Pang, L., Chen, Y., Kong, X., Chen, J. and Tian, X., 2020. Environmental Science and Pollution Research, pp.1-10. <https://doi.org/10.1007/s11356-020-08474-0>

Zhang, H., Kim, M.S., Sun, Y., Dowd, S.E., Shi, H. and Paré, P.W., 2008. Molecular Plant-Microbe Interactions, 21(6), pp.737-744. <https://doi.org/10.1094/MPMI-21-6-0737>

Zhang, H., Sekiguchi, Y., Hanada, S., Hugenholtz, P., Kim, H., Kamagata, Y. and Nakamura, K., 2003. Journal of Medical Microbiology, 53(4), pp.1155-1163. <https://doi.org/10.1099/ijs.0.02520-0>

Zhang, J., Liu, J., Meng, L., Ma, Z., Tang, X., Cao, Y. and Sun, L., 2012. The Journal of Microbiology, 50(2), pp.191-198. <https://doi.org/10.1007/s12275-012-1472-3>

Zhang, Z.D., Gu, M.Y., Zhu, J., Li, S.H., Zhang, L.J., Xie, Y.Q., Shi, Y.H., Wang, W. and Li, W.J., 2015. International journal of systematic and evolutionary microbiology, 65(5), pp.1572-1577. <https://doi.org/10.1099/ijs.0.000137>

Zhao, K., Li, J., Zhang, X., Chen, Q., Liu, M., Ao, X., Gu, Y., Liao, D., Xu, K., Ma, M. and Yu, X., 2018. Scientific reports, 8(1), pp.1-13. <https://doi.org/10.1038/s41598-018-32097-8>

Zhao, R., Chen, X.Y., Li, X.D., Tian, Y., Kong, B.H., Chen, Z.L. and Li, Y.H., 2014. International journal of systematic and evolutionary microbiology, 64(2), pp.607-612. <https://doi.org/10.1099/ijs.0.055749-0>

Zheng, B.X., Bi, Q.F., Hao, X.L., Zhou, G.W. and Yang, X.R., 2017. International journal of systematic and evolutionary microbiology, 67(8), pp.2514-2519. <https://doi.org/10.1099/ijsem.0.001916>

Zheng, B.X., Ibrahim, M., Zhang, D.P., Bi, Q.F., Li, H.Z., Zhou, G.W., Ding, K., Peñuelas, J., Zhu, Y.G. and Yang, X.R., 2018. AMB express, 8(1), p.47. <https://doi.org/10.1186/s13568-018-0575-6>

Zhou, G.C., Wang, Y., Zhai, S., Ge, F., Liu, Z.H., Dai, Y.J., Yuan, S. and Hou, J.Y., 2013. Applied microbiology and biotechnology, 97(9), pp.4065-4074. <https://doi.org/10.1007/s00253-012-4638-3>

Zhou, L., Yuen, G., Wang, Y., Wei, L. and Ji, G., 2016. Crop Protection, 84, pp.8-13. <https://doi.org/10.1016/j.cropro.2015.12.009>

Zhu, F., Qu, L., Hong, X. and Sun, X., 2011. Evidence-Based Complementary and Alternative Medicine, 2011. <https://doi.org/10.1155/2011/615032>

Zhu, L.J., Guan, D.X., Luo, J., Rathinasabapathi, B. and Ma, L.Q., 2014. Chemosphere, 113, pp.9-16. <https://doi.org/10.1016/j.chemosphere.2014.03.081>
